# Visualization-assisted binning of metagenome assemblies reveals potential new pathogenic profiles in idiopathic travelers’ diarrhea

**DOI:** 10.1186/s40168-018-0579-0

**Published:** 2018-11-08

**Authors:** Qiyun Zhu, Christopher L. Dupont, Marcus B. Jones, Kevin M. Pham, Zhi-Dong Jiang, Herbert L. DuPont, Sarah K. Highlander

**Affiliations:** 1 J. Craig Venter Institute, 4120 Capricorn Lane, La Jolla, CA 92037 USA; 2 Human Longevity, Inc., 4570 Executive Drive, La Jolla, CA 92121 USA; 3 University of Texas School of Public Health, 7000 Fannin St., Houston, TX 77030 USA; 4 Pathogen and Microbiome Division, Translational Genomics Research Institute, 3051 W. Shamrell Blvd., Suite 106, Flagstaff, AZ 86005 USA; 5 Department of Pediatrics, University of California San Diego, 9500 Gillman Drive #0763, La Jolla, CA 92093 USA; 6 Regeneron Pharmaceuticals, Inc., 777 Old Saw Mill River Road, Tarrytown, NY 10591 USA; 7 2132 Calaveras Ave, Davis, CA 95616 USA

**Keywords:** Travelers’ diarrhea, Virulence factor, *Escherichia coli*, TM7, crAssphage, Strain-level, Dark matter

## Abstract

**Background:**

Travelers’ diarrhea (TD) is often caused by enterotoxigenic *Escherichia coli*, enteroaggregative *E*. *coli*, other bacterial pathogens, Norovirus, and occasionally parasites. Nevertheless, standard diagnostic methods fail to identify pathogens in more than 40% of TD patients. It is predicted that new pathogens may be causative agents of the disease.

**Results:**

We performed a comprehensive amplicon and whole genome shotgun (WGS) metagenomic study of the fecal microbiomes from 23 TD patients and seven healthy travelers, all of which were negative for the known etiologic agents of TD based on standard microbiological and immunological assays. Abnormal and diverse taxonomic profiles in TD samples were revealed. WGS reads were assembled and the resulting contigs were visualized using multiple query types. A semi-manual workflow was applied to isolate independent genomes from metagenomic pools. A total of 565 genome bins were extracted, 320 of which were complete enough to be characterized as cellular genomes; 160 were viral genomes. We made predictions of the etiology of disease for many of the individual subjects based on the properties and features of the recovered genomes. Multiple patients with low-diversity metagenomes were predominated by one to several *E*. *coli* strains. Functional annotation allowed prediction of pathogenic type in many cases. Five patients were co-infected with *E*. *coli* and other members of Enterobacteriaceae, including *Enterobacter*, *Klebsiella*, and *Citrobacter*; these may represent blooms of organisms that appear following secretory diarrhea. New “dark matter” microbes were observed in multiple samples. In one, we identified a novel TM7 genome that phylogenetically clustered with a sludge isolate; it carries genes encoding potential virulence factors. In multiple samples, we observed high proportions of putative novel viral genomes, some of which form clusters with the ubiquitous gut virus, crAssphage. The total relative abundance of viruses was significantly higher in healthy travelers versus TD patients.

**Conclusion:**

Our study highlights the strength of assembly-based metagenomics, especially the manually curated, visualization-assisted binning of contigs, in resolving unusual and under-characterized pathogenic profiles of human-associated microbiomes. Results show that TD may be polymicrobial, with multiple novel cellular and viral strains as potential players in the diarrheal disease.

**Electronic supplementary material:**

The online version of this article (10.1186/s40168-018-0579-0) contains supplementary material, which is available to authorized users.

## Background

Travelers’ diarrhea (TD) is a major health concern for international visitors, especially for those who travel from industrial countries to developing regions such as Latin America, Africa, and South Asia. About one third of tourists develop diarrheal symptoms within two weeks after arrival. Despite advances in medical science and improvements in hygiene in developing countries, the rate of TD remains high [1]. TD patients suffer from frequent bowel movements, vomiting, nausea, and bowel pain. Although TD is usually self-limiting, in some cases it can lead to more severe complications such as irritable bowel syndrome, reactive arthritis, and Guillain-Barré syndrome [2, 3].

Multiple enteropathogens have been detected in TD stool samples [4, 5]. Enterotoxigenic *Escherichia coli* (ETEC) is the most common causative agent [6], being responsible for more than 30% of recorded cases [4], followed by enteroaggregative *E*. *coli* (EAEC) [5]. ETEC produces the plasmid-encoded heat-labile (LT) and heat-stable (ST) enterotoxins. Norovirus (NoV) causes approximately 5–8% of TD [7]. Infection with NoV has been associated with disruption of gut microbiota [8]. Other pathogens frequently reported in TD cases include *Shigella*, *Salmonella*, *Campylobacter*, non-cholera vibrios, and giardia [5]. Rare pathogens, such as *Arcobacter butzleri* and enterotoxigenic *Bacteroides fragilis* (ETBF), have also been associated with the disease [9].

Conventional lab techniques to assay for the presence of pathogens in stool specimens of TD patients include cultivation, polymerase chain reaction (PCR), and immunoassays [10]. Modern tools, such as the BioFire FilmArray Gastrointestinal Panel [11], are now utilized for pathogen identification. Nevertheless, a considerable proportion (up to 40%) of subjects test pathogen-negative [4, 12, 13]. This also holds true in other types of diarrheal [14] and gastrointestinal diseases that are believed to be caused by infectious agents. Studies showed that antibiotic treatment could effectively cure most cases of TD, both with known or unidentified pathogens [15]. Thus, it is reasonable to hypothesize that there are unknown bacterial pathogens responsible for some TD cases.

Metagenomic sequencing has emerged as a new approach to the characterization of the microbiome and the discovery of known and novel pathogens in the human gastrointestinal tract [16–19]. While cost-efficient 16S rRNA gene sequencing has proved its validity in identifying taxa, whole genome shotgun (WGS) sequencing provides more insight into the characterization of a microbial community in terms of predicted function and the identity of individual genomes. In particular, genomes with high divergence from any known organisms, or with gene-level differences compared to documented reference strains, can be identified by WGS sequencing [20].

WGS sequencing has successfully detected novel viral pathogens in human diseases [21–23]. It has been a less common choice for bacterial pathogen discovery, and as such, studies have typically been directed toward specific pathogens (e.g., *Clostridiodes difficile* [17] and *E*. *coli* O104:H4 [18]). Researchers have also scanned for novel organisms in metagenomic data [24]. However, most of these studies were based on queries of marker genes rather than whole genomes, so they failed to identify structural and functional changes (e.g., acquisition of antimicrobial genes).

We hypothesized that new individual pathogens, or combinations of organisms, might be responsible for the diarrheal symptoms in TD patients of unknown etiology. These putative pathogens could be: (1) known organisms with a previously unidentified etiology in TD; (2) known organisms carrying previously undocumented, or newly acquired pathogenicity factor genes; and (3) unknown or under-characterized organisms that could be potentially relevant to TD. This third class might include bacterial strains of unknown phylogeny, or microbial “dark matter” [25]. To examine these possibilities, we conducted a retrospective metagenomic survey of the gut microbiomes of TD patients and healthy traveler controls, using a combination of 16S rRNA gene and WGS sequencing. The goal was metagenomic discovery of new potential enteropathogenic candidates in TD patients where pathogens were not identified by traditional pathogen screens.

## Results and discussion

### 16S rRNA gene sequencing reveals diverse and abnormal gut flora composition

The stool samples used were described in a previous study and include 23 travelers’ diarrhea samples (TD) that tested negative for known diarrheal pathogens in routine clinical microbiological tests, plus seven healthy traveler controls (HT) (Additional file 1: Table S1) [12]. In the previous study, we tested each sample for the presence of the ETEC heat-labile and heat-stable toxin genes by a quantitation real-time PCR method [10] and all samples were negative. In this study, we also tested each sample for the presence of the *B*. *fragilis* toxin gene *bft* by PCR using a primer set that detects all three alleles of the gene. All samples were *bft* negative but were positive for amplification of the 16S rRNA gene.

**Fig. 1:**
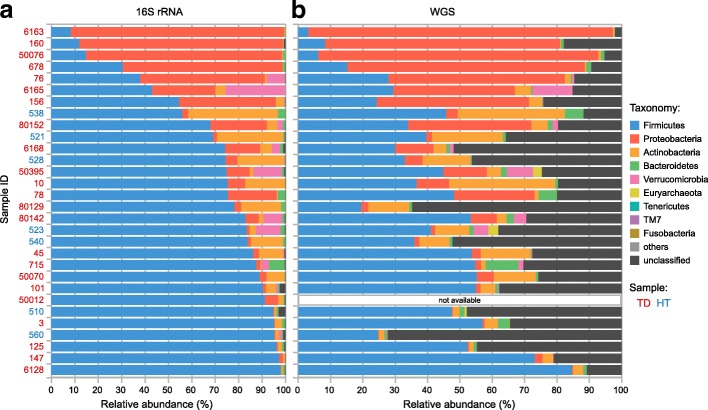
Phylum-level taxonomic profiles. Bar lengths represent relative abundances of sequences classified in taxonomic groups. **a** 16S rRNA gene-based profile, in which the baseline is the pool of all classified 16S rRNA sequences. Phyla with less than ten sequences in total are not displayed. “Unclassified” represents sequences marked as “unclassified Bacteria” by mothur. **b** WGS-based profile. Phyla with an average relative abundance lower than 0.001% are not displayed. “Unclassified” represents sequences not mapped to any of the reference sequences in the database. Samples are sorted by the 16S rRNA gene-based relative abundance of Firmicutes from low to high

We assessed the microbial community composition in the stool samples using two high-throughput sequencing strategies: amplicon sequencing targeting the 16S rRNA gene V4 region and WGS sequencing to recover the entire metagenome. 16S rRNA gene sequencing revealed a high abundance (≥ 15%) of Proteobacteria in 30% of the samples, with the remainder dominated by Firmicutes (Fig. 1a). The abundance of Bacteroidetes was low (< 10%) in all TD and HT samples, unlike the typical high abundance (ca. 30–90%) in nearly all human gut microbiomes of healthy adults sampled in the Human Microbiome Project (HMP) [26], as we previously reported [12].

Alpha diversity analysis of the 16S rRNA gene data revealed a mixed distribution of operational taxonomic unit (OTU) diversity, richness, and evenness across samples (Additional file 2: Figure S1). TD samples 6163, 160, 50076, and 678 were dominated by Proteobacteria and had low OTU richness and diversity, while samples 147 and 6128 also had low OTU richness and diversity and, conversely, were predominated by Firmicutes. No overall significant difference was observed between TD and HT groups in all indices.

**Fig. 2:**
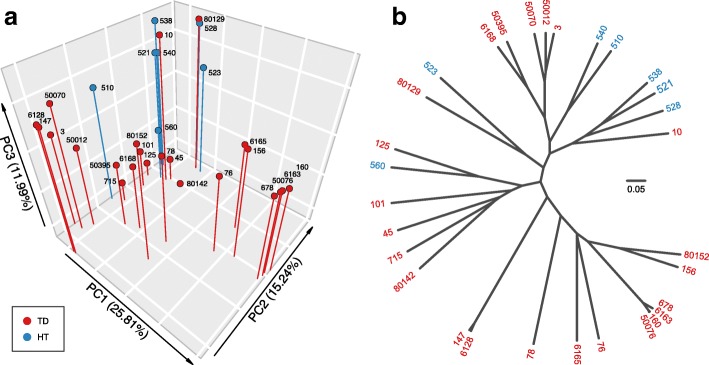
16S rRNA gene-based beta diversity of samples. **a** Scatter plot of the top three axes by principal coordinates analysis (PCoA). The four highly Proteobacteria-dominant samples, 160, 678, 6163 and 50076, formed a distinct cluster on the PC1 axis (vs. other TDs, AMOVA *p* value < 0.001). Three Proteobacteria-rich samples (76, 156, and 6165) also mapped near this cluster. The two Firmicutes-predominant samples, 147 and 6128, formed a small cluster (vs. other TDs AMOVA *p* value = 0.012). **b** Dendrogram reconstructed using the UPGMA algorithm based on the average Yue & Clayton measure of dissimilarity between pairs of samples

Principal coordinates analysis (PCoA) revealed clustering patterns of samples based on differential OTU composition and relative abundance (beta diversity) (Fig. 2a). The top three dimensions had a total loading of 53%, and showed that all seven even HT samples clustered, while the distribution of the 23 TD samples was diverse (TD vs. HT AMOVA *p* value = 0.003, HOMOVA *p* value = 0.031). A dendrogram further illustrated the similarity between samples (Fig. 2b). Proteobacteria-dominant and Firmicutes-dominant samples formed distinct clades and five of the seven HT samples clustered.

### Resolving microbiome composition and recovering individual genomes with WGS sequencing

Based on 16S rRNA gene profiles and differences in diversity metrics, we originally selected to split the samples into two tiers of sequencing depth based on diversity and phylum distribution. Samples with low alpha diversity were sequenced to relatively low depth (≥ 3 Gb) and those with high diversity were sequenced at greater depth (≥ 10 Gb). Samples with genomes of particular interest (e.g., 6128, 6163, and 50076) were then sequenced to greater depth following preliminary analysis. One sample, 50012, was not carried through to WGS. Sequencing statistics are shown in Additional file 1: Table S2.

To maximize the taxonomic classification of known organisms, we mapped WGS data against all available NCBI RefSeq genomes [27] (see Additional file 3: Supplemental Text). This increased the classification ratio compared to typical protocols, resulting in an average of 73.2% per sample (Fig. 1b, and Additional file 1: Table S2). Thirty-eight prokaryotic genera and 91 species were detected at a relative abundance ≥ 0.1% (Additional file 1: Tables S3 and S4). In several samples (such as 510, 528, 540, 560, and 80129, the first four of which are HT samples), a large proportion (max. 72.3%) of reads could not be classified, indicating the enrichment of the so-called dark matter. While this strategy was computationally challenging, it provided useful results. For example, the common human gut species *Faecalibacterium prausnitzii* [28] was detected at high relative abundance in this study (Additional file 1: Table S4), but it is missed using typical databases that contain only complete genomes (such as the standard databases of Kraken [29] and Centrifuge [30]) because its genome is still in draft status (GenBank: NZ_ACOP00000000).

*Escherichia* was the most frequently identified genus (Additional file 2: Figure S2) and was significantly more abundant in TD samples than in controls (one-tailed *t* test *p* value = 0.001). It was the highest in sample 6163, where 91.4% of the entire metagenome reads mapped to *Escherichia*. Other high-abundance genera were *Ruminococcus*, *Blautia*, and *Eubacterium*, all members of the order Clostridiales and common members of a healthy gut microbiome. The abundance of *Shigella* was directly proportional to that of *Escherichia* (*R*^2^ = 0.944). These two lineages are phylogenetically indistinguishable so it is most likely that the reads are of *Escherichia* not *Shigella* since it is not a usual cause of TD [31]. Three additional Enterobacteriaceae genera, *Enterobacter*, *Klebsiella*, and *Citrobacter*, were enriched in TD samples 10, 76, 78, and 80152 (sum of the three genera vs. other TD samples, one-tailed *t* test *p* value = 0.097). We also searched the reads against reference genomes of known DNA viruses other than bacteriophage (note that RNA viruses could not be detected by WGS sequencing and these could be potential causes of disease), but observed very few mappable reads. The top hits were to a polydnavirus that infects wasps, *glypta fumiferanae ichnovirus*, and to human retrovirus K; low proportions of reads mapped to additional reference eukaryotic viral genomes as shown in Additional file 2: Figure S3. These were equally distributed between diarrheal samples and healthy controls and none mapped to viruses known to cause diarrhea.

### Metagenome assembly and functional profiles

We first performed de novo metagenome assembly using IDBA-UD [32] of the 29 samples in an attempt to identify potential pathogenic species. An average of 3.52 k contigs were ≥ 1 kb per Gb of reads. Notably, 0.28% of all contigs could be circularized based on their overlapping ends (Additional file 1: Table S2). Open reading frames (ORFs) were predicted from the contigs, and were annotated using a variety of general and specific databases. Functional profiles were built to summarize the overall density of functional features in each metagenome (Additional file 1: Table S6 and Additional file 2: Figure S4). The majority of diarrheal samples, especially those with a high concentration of *Escherichia*, clustered to the left side of the plot, while the majority of healthy controls were located at the right. The diarrheal samples were enriched in carbohydrate, energy, nucleotide, and amino acid metabolism and some had higher proportions of membrane transport systems. They also had more predicted virulence factor, antibiotic resistance, and plasmid genes, while healthy samples had higher relative abundances of DNA viral genes.

### Isolating genomes from metagenomes by binning

**Fig. 3:**
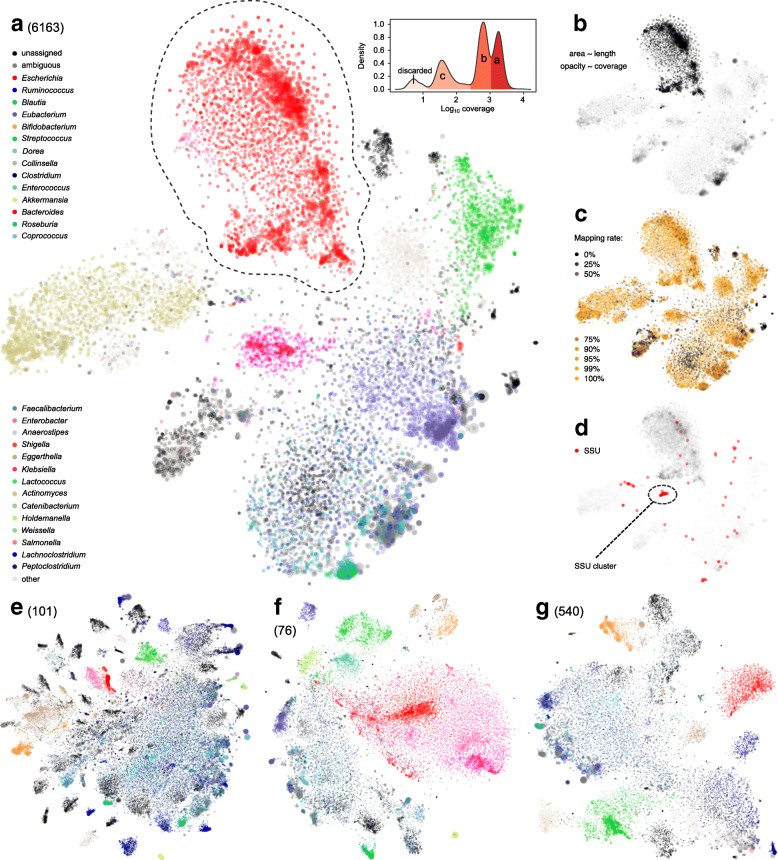
Illustration of metagenomic contig clustering pattern and binning process. **a**–**d** VizBin-computed, *k*-mer signature-based scatter plots of contigs ≥ 1 kb of the low-diversity sample 6163, in which *E*. *coli* was the dominant species (91.3%, by WGS reads, same below) and multiple *E*. *coli* genomes were detected and separated. The area of each dot is proportional to the contig size. **a** Taxonomic assignments of contigs. Genera with relative abundance ≥ 0.2% are colored. A contig is colored if ≥ 75% of reads mapped to it were mapped to a single genus. The dashed area shows a manually selected cluster of mostly *Escherichia* contigs. The kernel density function of the *Escherichia* contigs is plotted aside, with peaks manually divided to represent genomes of multiple *E*. *coli* strains. **b** Contig coverage indicated by opacity. **c** Taxonomic assignment rate (proportion of reads mapped to the reference genome database) indicated by color depth. **d** Contigs with SSU(s) are highlighted. **e** High-diversity sample 101 from which multiple known and “dark matter” genomes were isolated. **f** Sample 76 featured by the presence of multiple Enterobacteriaceae genera. **g** Sample 540, a healthy traveler control with moderate diversity

As noted here (Additional file 2: Figure S5), and by others, current binning tools usually fail to produce congruent results [33, 34]. Therefore, we decided to use VizBin to visualize assembled contigs as a two-dimensional scatter plot based on their *k*-mer signatures [35]. A bioinformatics pipeline was designed and utilized, which involved intensive manual observation and curation, with assistance from multiple programs and in-house scripts to identify, extract, reassemble, analyze, classify, and ultimately re-annotate individual genomes from each metagenomic sample (Fig. 3).

Based on the visual information, spatially clustered contigs with distinct coverage, taxonomic assignment, or other features that might represent individual genomes were manually selected and extracted (Fig. 3a). To further separate closely related genomes (those having similar *k*-mer signatures), the density of coverage for each genome was plotted. Distinct peaks that could represent different genomes were manually isolated (inset of Fig. 3a). The quality of each isolated genome (a.k.a. bin) was evaluated using CheckM [36] and then manually examined and compared to taxonomically related reference genomes to assess their biological properties. These data were utilized to guide the further purification of each bin and were applied iteratively until the binning quality was improved to a maximum level. A flowchart illustrating the binning and assembly method is shown in Additional file 2: Figure S6.

We extracted putative genomes, based on the following criteria: (1) clusters of contigs that were spatially isolated in the plot (thus easily separable); (2) standalone large contigs with notably high coverage compared to the background (candidates for plasmids or phages); (3) clustered contigs mapped to taxonomic groups that might contain known pathogens; (4) clustered contigs were enriched for signals for virulence genes, antimicrobial resistance genes, plasmid, and/or virus; and (5) contigs that shared sequence similarity with particular bins in other samples.

There were limitations. Genomes that were highly fragmented, those with low coverage, or those that were closely related to other genomes in the same sample without low level taxonomic resolution were difficult to isolate from the background. Genomic islands (which frequently carry pathogenicity genes), such as integrated plasmids and bacteriophages, often have distinct *k*-mer signatures from their host genomes, making it challenging to infer the correct host associations (examples are described below).

We observed a variety of clustering patterns (Fig. 3 and Additional file 4: File S4). Generally, the number of distinct contig clusters was positively correlated with the alpha diversity of the sample (Additional file 2: Figure S1, Fig. 3a, e–g; number of bins vs. inverse Simpson index: *r* = 0.693, *p* value = 3.09 × 10^−5^). The spatial separation of a genome from all other contigs was positively correlated with uniqueness of its taxonomy in the community. For example, multiple members of the Firmicutes (e.g., *Blautia*, *Dorea*, and *Enterococcus*) mapped in regions with undistinguishable contigs (Fig. 3a, e–g), whereas taxonomic groups without many relatives in the human gut, such as *Akkermansia* (Verrucomicrobia) and *Bifidobacterium* (Actinobacteria), formed distinct clusters (Fig. 3e–g).

**Fig. 4:**
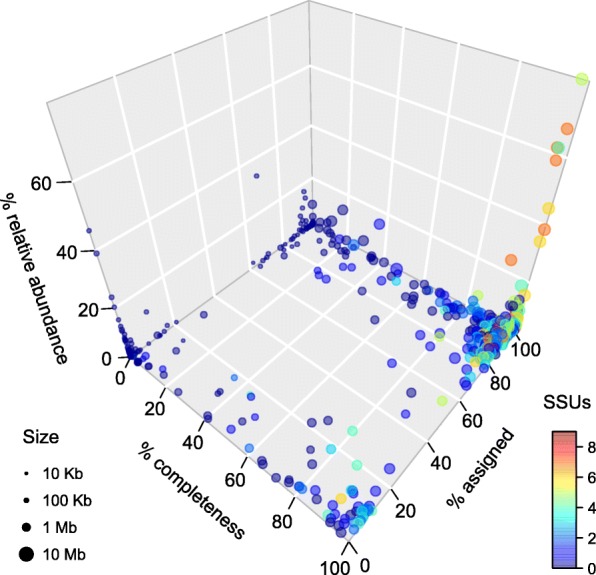
Basic statistics of the 565 genome bins extracted from 29 metagenomes. The three axes indicate relative abundance (calculated as sum of length × coverage of member contigs, normalized by the whole assembly), CheckM-computed completeness, and taxonomic assignment rate (proportion of classifiable reads mapped to member contigs), respectively. Dot area is proportional to the total length of contigs of each bin. Color scale indicates the number of SSUs identified in each bin

A total of 565 genome bins ranging from 6.28 kb to 6.70 Mb in length were isolated from the 29 metagenomes (Fig. 4 and Additional file 1: Table S7). The highest number of bins (*n* = 69) was extracted from sample 101 (the most diverse), followed by samples 715 (*n* = 44) and 3 (*n* = 32). Sample 147 and 80152 had the lowest number of bins (*n* = 9). On average, 56.1% of WGS reads per sample were mapped to contigs included in bins. SSU rRNA genes were identified in 266 bins. A total of 118 bins were composed of single contigs with an average length of 94.2 kb, and none of them contained SSU rRNA genes. Taxonomic assignment rates revealed a highly bimodal distribution (Fig. 4): of the 565 bins, 83 had an assignment rate above 99%, 60 between 95 and 99%, while 6 bins contained no assigned reads, and 89 had an assignment rate below 0.1%.

### *Escherichia coli* strains: assembly and “genetic pathotyping”

**Fig. 5:**
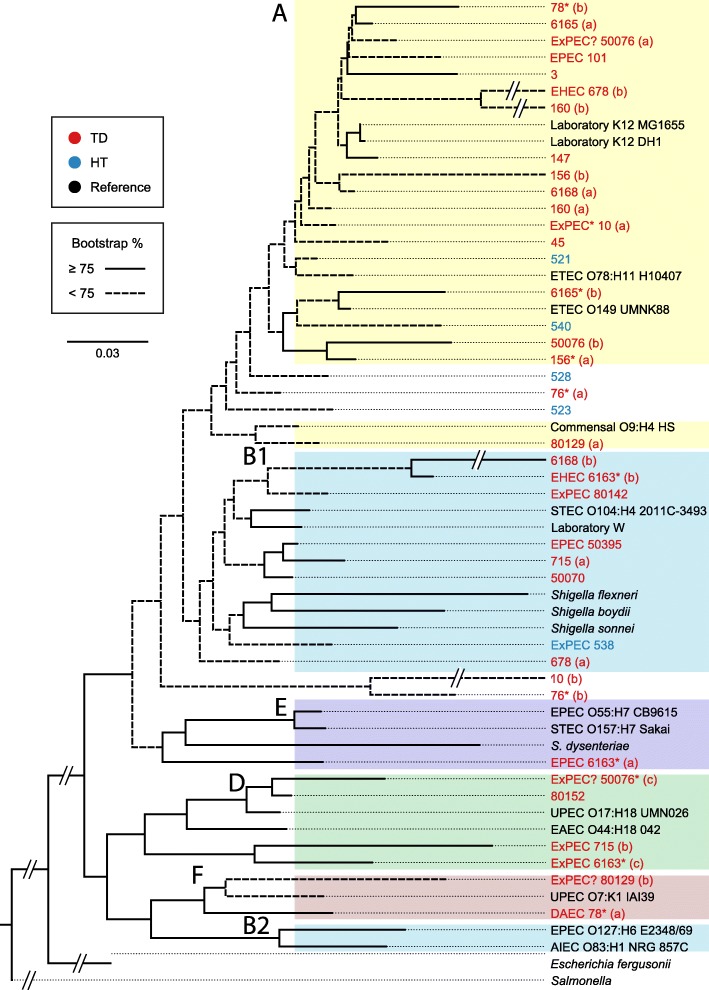
Phylogenetic tree of identified *E*. *coli* genomes. The tree was reconstructed using the maximum likelihood method using a conserved set of protein sequences. Multiple reference *E*. *coli* genomes were included to indicate the phylogenetic positions of the identified *E*. *coli* strains. Only near-complete (completeness ≥ 80%) genomes were included in the analysis. The tree is rooted with *Salmonella* as an outgroup. Nodal labels represent bootstrap support values (out of 100 replicates). Strains marked with an asterisk were those that were part of a polymicrobial sample. Group A is shaded yellow, B1 and B2 blue, D is green, E is violet and F is peach

We detected *E*. *coli* as the predominant species in nine TD samples: 76, 78, 156, 160, 678, 6163, 6165, 50076, and 80152; its relative abundance was more than five-fold higher than the next most abundant species. Based on the presence of predicted virulence genes (Table 1 and Additional file 1: Table S8), we postulate that some strains could be the cause of diarrhea. Using the binning pipeline, we were able to separate multiple *E*. *coli* genomes co-infecting eight TD samples. In six samples, two *E*. *coli* genomes were isolated. In samples 6163 and 50076, where *E*. *coli* had the highest relative abundance, three *E*. *coli* genomes were isolated (Additional file 1: Tables S7 and S8). We used suffixes (a), (b), and (c) to describe the primary, secondary, and tertiary *E*. *coli* genomes per sample. Typically, when multiple *E*. *coli* genomes were present, the highest-coverage genome was recovered with completeness close to 100%, while the others were partial (completeness 4.2% to 36.4%). Nevertheless, unique features were obtained in these partial genomes. We isolated 24 near-complete *E*. *coli* genomes from the assembled samples (Additional file 1: Table S7). Nine of these could be aligned to known complete genomes (Additional file 2: Figure S7). A phylogenetic tree of these near-complete and partial *E*. *coli* genomes, together with *E*. *coli* reference genomes, was constructed based on shared marker genes from the whole genomes (Fig. 5). The tree shows the established clustering of *E*. *coli* phylogenetic groups, A, B1, B2, E, D, and F [31, 37]. We did not observe any C phylogroup members in our samples. Human commensals are mostly found in phylogroup A [38, 39] but some are also found in B1. Several of our predicted pathogenic strains (see the following paragraph) clustered in phylotype A but the bootstrap scores were very low. Note too that the two ETEC reference strains also clustered with phylogroup A; this is reasonable since the ETEC toxins are plasmid-borne. However, most animal-associated *E*. *coli* strains are also members of phylogroups B1. B2 and D are the predominant phylotypes in wastewater [40, 41] and many extraintestinal pathogens fall within this group [42]. A more recent typing scheme broke out additional groups C, E, and F and these also contain pathogenic *E*. *coli* pathotypes [37].

**Table 1:** Features of predicted pathogenic *E*. *coli* strains by sample. Relative abundance, predicted serotype, predicted MLST type, and predicted pathogenic type are reported. Extended detail is provided in Additional file 1: Tables S8-S10

*E. coli* bin	Relative abundance (%)	Predicted serotype	Predicted MLST type	Predicted pathogenic type
10 (a)	6.28	H4	ST-10	ExPEC
10 island	0.06	NT	NT	TSS genes
78 (a)	17.63	O1:H7	Unknown	DAEC
78 island 2	0.06	NT	NT	EHEC gene
101	1.05	O162:H33	ST-378	EPEC
101 island	0.01	NT	NT	EHEC genes
538	3.37	O89:H33	NT	ExPEC
678 (b)	13.12	O69:H5	NT	EHEC
678 island	3.52	NT	NT	ExPEC genes
715 (b)	0.12	H15	NT	ExPEC genes
715 island	0.02	NT	NT	EHEC genes
6163 (a)	78.90	O145	ST-10	EPEC
6163 (b)	14.70	O111:H8	NT	EHEC
6163 (c)	0.73	O166:H15	NT	ExPEC
6165 island	0.66	NT	NT	EPEC/EAEC genes
6168 (b)	0.78	O111	NT	EPEC?
50076 (a)	67.00	H2	ST-10	ExPEC?
50076 (c)	4.54	O99:H33	NT	ExPEC?
50076 island 1	8.97	NT	NT	ExPEC genes
50076 island 3	0.26	NT	NT	TTSS genes
50395	15.00	H8	ST-590	EPEC
80129 (b)	0.15	H34	NT	ExPEC/NMEC
80142	8.70	H8	Unknown	ExPEC
80142 island 1	0.14	NT	NT	EPEC genes

Defining features and definitions:

Enterotoxigenic *E*. *coli* (ETEC): heat labile toxin, heat stable toxin

Enteroaggregative *E*. *coli* (EAEC): Aaf fimbriae, dispersin

Enteropathogenic *E*. *coli* (EPEC): LEE, STX-, bundle-forming pilus

Enterohemorrhagic *E*. *coli* (EHEC): LEE, STX+, Efa1 adhesin, ToxB

Diffusely adherent *E*. *coli* (DAEC): Afa/Dr. fimbriae

Neonatal meningitis *E*. *coli* (NMEC): K1 capsule, Ibe invasion proteins

*LEE* locus of enterocyte effacement, *TTSS* type three secretion system, *STX* Shiga toxin, *ND* not determined, *NT* not tested, ? probable but not conclusive

The genomes were searched for matches to *E*. *coli* and *Shigella* virulence factor sequences in an effort to place them into one of the six major categories of diarrheagenic *E*. *coli*’s: enterotoxigenic (ETEC), enteroinvasive (EIEC), enteropathogenic (EPEC), enterohemorrhagic (EHEC), enteroaggregative (EAEC), and diffusely adherent *E*. *coli*’s (DAEC) [43]. We also observed patterns consistent with extraintestinal *E*. *coli* (ExPEC) and the K1 capsule expressing pathotype known as neonatal meningitis-associated *E*. *coli* (NMEC) [44]. The results are summarized in Table 1 and Additional file 1: Table S8. As expected, no ETEC strains were discovered. Because the ETEC toxins are plasmid-borne, we conducted a further BLAST search of the complete metagenome of each sample using the heat labile (LT) and heat stable genes (ST) as queries and the genes were not detected. Some strains were reasonably easy to categorize, while others were not easily classified or were marked as non-pathogens. For example, strains *E*. *coli* 678 (b) and *E*. *coli* 6163 (b) appear to be EHEC pathotypes since they encode both the entire locus of enterocyte effacement (LEE) and the Shiga toxin subunits A and B, characteristic of this pathotype, while *E*. *coli* 101, 6163 (a), and 50395 (a) are likely EPEC strains because they carry the LEE but lack the toxin genes [45] (note that the relative abundance of *E*. *coli* 101 is only 1.0%). Strain *E*. *coli* 78 (a) was predicted to be a DAEC pathotype because it encodes the Afa/Dr. fimbriae and lacks the LEE island [46]. The remaining genomes with pathogenic features fall into the ExPEC group. This category is defined as those that have different combinations of a set of virulence factors that include type 1 fimbriae, P fimbriae, S fimbriae, F1C fimbriae, D3 adhesins, K1 capsule, hemolysin HlyCABD, and aerobactin receptor [47]. Genomes that possess two or more of these features are *E*. *coli* 10 (a), 6163 (c), 80129 (b), 80142, and 538 (the last of which is from a healthy traveler). Strain *E*. *coli* 80129 (b) may be a member of the NMEC pathotype since it is predicted to encode the invasion protein IbeA and the K1 capsule [44]. In addition, we noticed that the *E*. *coli* strain within HT sample 538 carries an RTX-type hemolysin gene cluster plus the type 1 fimbriae so it could be classified as ExPEC.

In silico serotyping and multilocus sequence typing (MLST) using the Achtman scheme described in [48] was performed (Table 1, Additional file 1: Tables S9 and S10). Serotyping and MLST typing have historically been used to differentiate *E*. *coli* commensals and pathogens. We were only partially successful in predicting MLST types of the potentially virulent strains. This information classified genomes *E*. *coli* 10 (a), 147, 6165 (a), and 50076 (a) as MLST Type ST-10; 10 (a) and 50076 (a) were predicted to be ExPEC strains. Virulent ST10 strains have been reported in extraintestinal clinical samples such as blood and urine [49], but they are also appearing as dominant extended spectrum beta-lactamase producing *E*. *coli* strains in feces in some populations [50, 51]. The other strains that could be typed (*E*. *coli* 101, 156, 521, 528, 538, 50395, and 80152) were rare STs, so their significance in virulence is unknown.

We classified *E*. *coli* strain in sample 78 as DAEC and its predicted serotype O1:H7 is consistent with a known clonal group of avian pathogenic *E*. *coli* (APEC) strains that cause extraintestinal infections [52]. Serotype O145 is usually associated with EHEC but since we did not detect the Shiga toxin gene in the genome of *E*. *coli* 6163 (a), we characterized the strain as an EPEC. The serotype of *E*. *coli* 6163 (b), however, was predicted to be O111:H8, consistent with EHEC strains. We also predicted that strain *E*. *coli* 6168 (b) was serotype O111, but categorized it as a presumptive EPEC since it lacked the toxin gene. *E*. *coli* serotype O166:H15, associated with strain *E*. *coli* 6163 (c), has only once been reported as a cause of diarrhea [53] and all of the isolates identified in that study carried the EAggEC heat-stable enterotoxin (EAST1) gene, which was not found in our strain. A similar finding was reported, also only once [54], in an EAST1 positive 089:H33 strain, like our strain *E*. *coli* 538 (but again lacking the EAST1 gene).

In addition, we observed multiple samples that had smaller clusters of contigs spatially separated from the *E*. *coli* chromosome cluster in the scatter plots, but which were taxonomically assigned to *E*. *coli*. We recovered 17 such “islands” from 14 samples (Additional file 1: Table S8), including 2 very large ones: *E*. *coli* 678 island (550 kb, relative abundance 3.5%) and *E*. *coli* 50076 island 1 (1.36 Mb, relative abundance 9.0%). Thirteen of the islands were from TD samples; only one was from a HT sample. The islands contained few to no *E*. *coli* core genes, but many TD islands carried predicted virulence genes (Additional file 1: Tables S7 and S8). Their annotations usually related them to phage or plasmids. Except for a few cases such as an obvious *E*. *coli* plasmid in sample 6168, which is 99% identical to an 18 kb region of the 120 kb unnamed multiple antibiotic resistance plasmid of the Shiga toxin-producing *E*. *coli* reference strain 2009C-3133 [55], it is unknown whether these islands are autonomous self-replicating elements or merely parts of the main *E*. *coli* chromosome with distinct *k*-mer signatures.

All of the assembled *E*. *coli* genomes carry the *ampC* gene that is predicted to encode a Class C beta-lactamase. One, *E*. *coli* 156 (a), has an integron element with genes encoding predicted chloramphenicol and streptomycin resistance. All genomes have potential multidrug transport protein genes but it is challenging to predict their roles in antibiotic resistance.

### Mixed infections with other Enterobacteriaceae species

Among Enterobacteriaceae members other than *E*. *coli*, we observed samples that contained *E*. *coli* plus various combinations of *Enterobacter*, *Klebsiella*, and *Citrobacter* species, which are not common intestinal pathogens (for example see Fig. 3f). Two TD samples, 76 and 78, had high total relative abundances of *Enterobacter*, *Klebsiella*, and *Citrobacter* (23% in sample 76 and 5.4% in sample 78). Of interest, the relative abundance of the three genera in these samples had similar relative ratios: 36:25:16 in sample 76 and 46:26:18 in sample 78. Sample 10 contains *Klebsiella pneumoniae* and *Enterobacter cloacae* in addition to *E*. *coli*, and 80152 contains only *E*. *coli* and *Enterobacter* sp. We also observed scaffolds of the three genera in sample 156 but none of these were present at > 1% relative abundance so they could not be confidently binned. Sample 78 also had the gram-positive opportunistic pathogens *Enterococcus faecium* and *Enterococcus faecalis* present at relatively high levels (14.5 and 4.4%, respectively), but these could not be confidently separated for genome level assembly.

Bins representing the Enterobacteriaceae organisms were isolated. Since they share similar *k*-mer signatures, they formed large “clouds” of contigs in the scatter plots (e.g., see Fig. 3f); this increased the challenge of separating them. Therefore, we relied mainly on coverage and taxonomic assignment to guide binning, which yielded suboptimal results. Of interest, there were at least two genomes each of *Enterobacter*, *Klebsiella*, and *Citrobacter* in sample 76 (Additional file 1: Tables S7 and S9). The relative abundance ratios of the major vs. minor bins were 5.1 (*Enterobacter*), 3.9 (*Klebsiella*), and 16.2 (*Citrobacter*). Based on the high contamination score of the *Enterobacter* bin (46.35%, Additional file 1: Table S7), we believe that there was more than one *Enterobacter* genome in sample 78; however, they could not be confidently separated. Note that samples 76, 156, and 80152 had non-pathogenic *E*. *coli* present at greater than 20% relative abundance (Additional file 1: Table S8). Samples 10 and 78 had lower relative abundances of *E*. *coli* (6.3 and 17.6%, respectively) and theses were predicted to be ExPEC and DAEC pathotypes, respectively. Reads for two other important pathogenic Enterobacteriaceae members, *Salmonella* and *Yersinia*, were not discovered at the genome level in any samples.

We also examined the Enterobacteriaceae genomes for the presence of potential virulence factors and antibiotic resistance genes (Additional file 1: Table S11). No compelling features such as toxin genes were observed, although some potential colonization factors, such as type I fimbriae (*K*. *pneumoniae*) and aerobactin receptor genes, were annotated. The *K*. *pneumoniae* strains in samples 10 and 76 both carried the gene encoding the extended spectrum beta-lactamase, SHV-1, and in samples 76 and 78, the *Enterobacter* genomes also contributed genes encoding predicted resistance to chloramphenicol. Several genomes also carried the *ampC* beta-lactamase gene. These genera have been observed together in fecal samples during a hospital outbreak [56] and are occasionally found in the preterm infant gut [57], but it is difficult to understand how they would be acquired in the context of travelers’ diarrhea. Also, with the exception of sample 76 (total abundance 22.2%), they are all present at very low abundance (ca. ≤ 2% relative abundance per taxon).

These non-diarrheal Enterobacteriaceae plus the enterococci observed in sample 78 are reminiscent of the facultative anaerobes that were described by David et al. following *Vibrio cholerae* infection [58]. In a metagenomic study, they reported the ordered succession of microbial communities following cholera diarrhea. They defined three stages of succession, early, mid, and late-stage, which were characterized by distinct microbial communities. The early-stage community is characterized by blooms of Enterobacteriaceae, enterococci, and streptococci capable of growth in the presence of oxygen and with elevated carbohydrate metabolism. Note that samples 76, 78, and 10 mapped to the left of the metabolic profile in Fig. 4 indicating a high relative proportion of carbohydrate metabolism genes so these may represent opportunists of succession and not potential pathogens. This may also be the case of the *E*. *coli* strains in samples 160, 50076, 678, 6163, 50070, 3, 101, and 147 that lack predicted virulence factors.

### “Dark matter” cellular organisms and a potentially pathogenic new TM7 strain

The expansion of sequenced microbial genomes has been accompanied by the appearance of a tremendous volume of “dark matters”: microbes that remain unknown or under-characterized due to challenges in sample collection, isolation, cultivation, and sequencing [59]. Phylogenetic analyses have placed novel organisms in proximity to known taxonomic groups—thereby expanding the “tree of life”—but have also revealed striking clustering patterns of a large number of deep branches, known as the candidate phyla radiation (CPR), which includes microbes that are substantially different from microbes that have been previously characterized [60, 61].

**Fig. 6:**
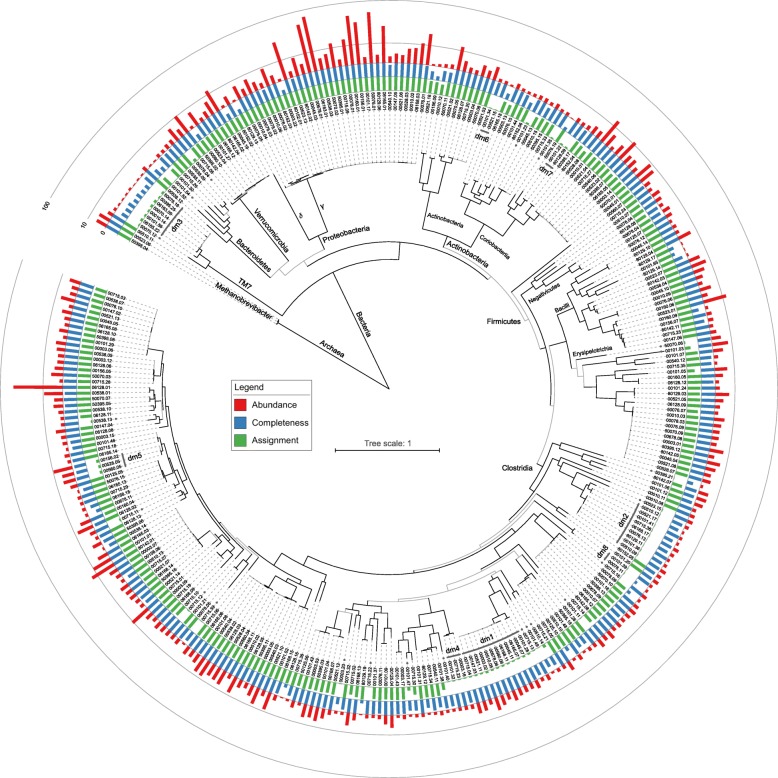
Phylogenetic tree of 320 bins representing cellular organisms. Taxon labels are sample ID dot bin ID (see Additional file 1: Table S7). Black and gray lines represent branches with ≥ and < 75 out of 100 bootstrap support, respectively. Branch labels are taxonomic groups to which all child taxa except for unidentified organisms belong. The circular bar plots represent relative abundance (red, square root scale), completeness as a cellular organism (blue, linear scale), and proportion of reads mapped to the reference genome database (green, linear scale). All three plots are in a 0 to 100% range. Unidentified organisms (assignment < 40%) are indicated by gray lines (clusters) and dots (singletons) around the circle

We extracted 320 bins that contained at least 10 of the 56 single-copy marker genes universally present in cellular organisms, and reconstructed a phylogenetic tree based these genes (Fig. 6 and Additional file 2: Figure S8). The genomes could be categorized by their taxonomy as (1) known and cultivated organisms with well-defined classification (e.g., *E*. *coli*), (2) organisms previously known only from metagenomes (e.g., bacterium LF-3) [62], and (3) dark matter organisms, which are those composed of contigs with low mapping rate to the entire reference sequence database (Additional file 1: Table S7).

Using a criterion of taxonomic assignment rate < 40% (see Additional file 3: Supplemental text), we identified 62 bins that represent dark matter cellular organisms; these include 8 phylogenetic clusters (groups dm1 to dm8) and 22 singletons (Fig. 6, Table 2 and Additional file 1: Table S7). The majority of the dark matter genomes, including five of the eight phylogenetic clusters, are members of the Clostridiales order. Despite being nested within known phyla or classes, multiple dark matter lineages are phylogenetically distant (as evident by long branch lengths) from their closest known sister lineages, suggesting that they represent novel organism groups at high taxonomic ranks. There were cases where two separable members of the same group co-exist within the same sample (Additional file 1: Table S7). The high occurrence rate of certain dark matter groups (e.g., dm1 and dm2) indicates that they may be frequent dwellers of the human gut. A high abundance of certain dark matter organisms was observed in one or more samples (e.g., dm5 ranges 3.6–9.3% in three samples). Several dark matter groups were found only in the diarrheal samples but not in the HTs. These are dm3 (TM7-like), dm4 (unclassified), and dm7 (unclassified by read mapping, but phylogenetically clustered with *Coriobacteriia*).

**Table 2:**
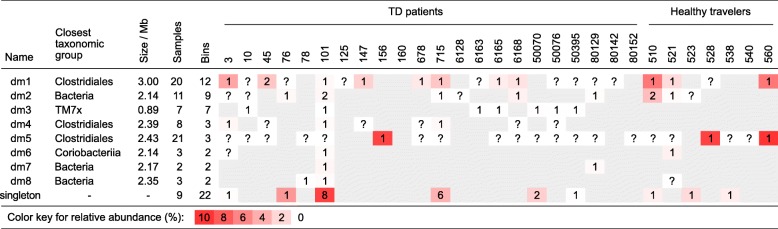
Putative cellular “dark matter” genomes identified in the metagenomes. Eight dark matter (dm) groups representing monophyletic, closely related genomes are listed, along with 22 singleton genomes that are also included in the phylogenetic tree (Fig. 6). The size of a group is calculated as the maximum size of its member bins. Numeric cell values represent the number of bins isolated per sample. Question marks indicate that there is clear evidence (clusters of contigs with high sequence similarity with other member genomes) that at least one genome is present in this sample. However, it was not isolated as bin(s) or included in the phylogenetic analysis because its relative abundance or completeness is low, or because its member contigs are mixed with those from other genomes in the plot, making it difficult to separate. The background color depth is proportional to the total relative abundance of the genome(s)

Seven TD samples contained dm3 group genomes (10, 101, 6163, 6165, 50395, 50070, and 50076) that we were able to classify as members of the enigmatic candidate phylum *Saccharibacteria* (a.k.a. TM7) [63]. All of these samples carried strains that clustered with the reference strain TM7x, originally isolated from the oral cavity [64]. Sample 50070 contained an additional, novel strain that we were able to separate and name as TM7z (Fig. 6 and Additional file 2: Figure S9, inset). Phylogenomic analysis revealed that its nearest phylogenetic neighbor is Candidatus *Saccharimonas aalborgensis* (Sab), which was isolated by metagenomic assembly from an activated sludge bioreactor sample [65] (Additional file 2: Figure S9). The TM7z genome is smaller than that of Sab (755 kb vs. 1.01 Mb) but it encodes several potential virulence features, including a predicted RTX family adenylate cyclase and its associated transport proteins, and *Listeria*-like internalin proteins. Unlike Sab, the TM7z genome has a limited metabolic and biosynthetic repertoire, suggesting that like TM7x, it must have an epibiotic lifestyle. TM7x can be co-cultured with *Actinomyces odontolyticus* [64]*.* We speculate that TM7z may share this dependence as we observed a distinct and high-abundance cluster of *Actinomyces* contigs in sample 50070 (Additional files 1 and 4: Table S7 and File S4). Confirmation of the pathogenic potential of TM7z will require isolation of the organism and further mechanistic studies.

In addition, we observed bins matching multiple co-abundance gene groups (CAG) organisms, which were originally identified based on the combination of a large set (396) of human stool metagenomes [62]. Our phylogenetic tree (Fig. 6) contains 34 CAG species, 12 of which form clusters; the remaining 22 are singletons. Several taxa were observed repeatedly, often in TD samples. Firmicutes bacterium CAG:41 was detected in 12 of 22 TD samples (nine included in the phylogeny), but in none of the controls (one-tailed Fisher’s exact test *p* value = 0.012, same below). *Blautia* sp. CAG:37 was found in 15 TD samples and two of seven controls (*p* value = 0.080). It was the highest in sample 147 (5.1%), and also high in samples 3, 45, and 715 (> 2%). None of these samples appear to contain pathogenic *E*. *coli* or other Enterobacteriaceae strains (see above). The dark matter group dm1 was also high (> 2%) in these four samples when compared to the other TD samples.

### Putative viral genomes

Viruses are frequent parasites of all three domains of cellular organisms and are common vectors of pathogenicity. Shotgun metagenome sequencing has enabled large-scale discoveries of novel viruses from human-associated [66] and environmental samples [67]. A notable example is crAssphage, a 97 kb phage that was found to be pervasive and highly abundant (1.68%) in healthy human guts [68]. In this study, we confirmed the prevalence of crAssphage in the guts of healthy travelers (but less so in diarrheal guts), and in addition discovered many additional viral genome clusters and singletons, some of which seemed to be related to crAssphage, based on *k*-mer signature and length.

**Fig. 7:**
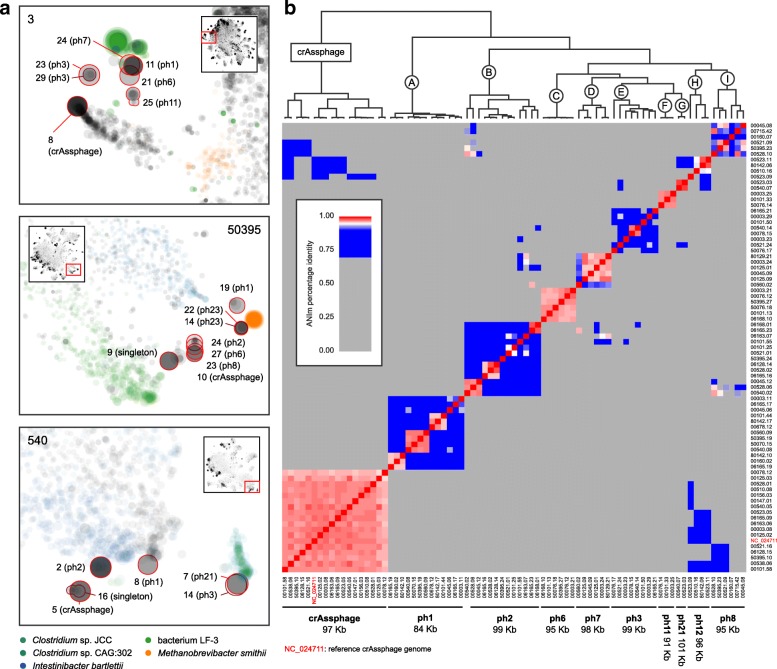
Clustering patterns of crAssphage and “crish” viruses. **a** Examples of the contig co-clustering patterns in the *k*-mer signature-based scatter plot in samples 3, 50395, and 540. The large panels are the zoom-in views of the red boxes in the small panels, which represent the entire microbiomes. The size and opacity of a dot are proportional to the length and coverage of the contig, respectively. Contigs mapped to five representative bacteria in proximity to the viruses are colored. Extracted virus bins are highlighted by red edges and labeled by the bin ID and the virus cluster name. **b** Pairwise average nucleotide identity (ANI) matrix of crAssphage’s and nine clusters of “crish” viruses (assigned by letters A to I). ANI values below 70% are grayed out. The dendrogram shows the hierarchical clustering result based on the ANI matrix. The reference crAssphage genome is included for comparison. Bins that are too fragmented, incomplete, and/or low abundance are not included. Singletons are not included

In the scatter plots of contigs, we identified multiple single, large (dozens to a few hundred kb), high-coverage contigs that are visually distinguishable from the background (Fig. 7). Many of them could be circularized (Additional file 1: Table S2). With a few exceptions, they do not share noticeable sequence similarity with any bacterial reference genome (Additional file 2: Figure S10). Their annotation tables, despite being enriched with “hypothetical proteins,” typically contained virus-related genes. Taken together, these observations suggest that they are viral genomes.

We extracted and curated the putative viral genomes from these contigs and their homologs from all samples. A total of 163 putative viral genomes were obtained, of which 142 could be grouped into 25 clusters based on sequence similarity (ANI ≥ 70% within a cluster); the remaining 21 were singletons (Table 3). The largest cluster was assigned to the crAssphage [68]. Seventeen crAssphage bins were found in 16 samples (sample 125 contains 2), and six were single, circular contigs. The remaining 24 clusters contain two to 15 virus bins each and were found in one up to 13 samples. The genomes range from 18 to 285 kb and are designated ph1 to ph24.

**Table 3:**
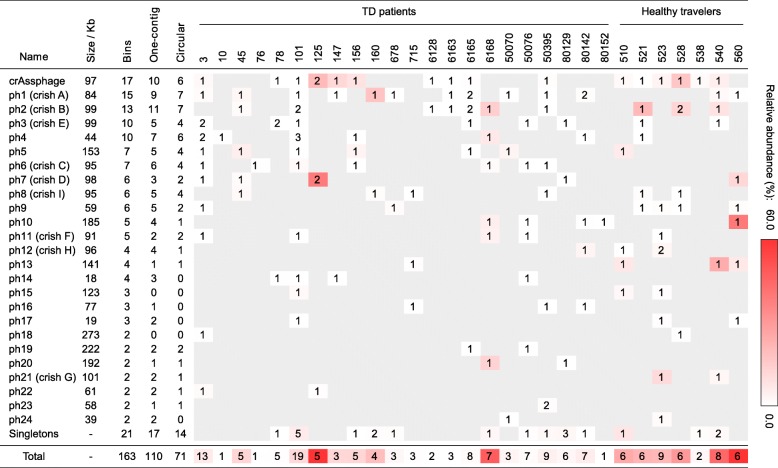
Putative viral genomes isolated from the metagenomes. crAssphage and 24 novel putative virus groups (namely ph1 to ph24, sorted by the number of isolated genomes (bins) from high to low), as well as 21 singleton putative viral bins are listed. Cell values represent the number of bins per sample. The background color depth is proportional to the total relative abundance of the genome(s). The size of a group is calculated as one if there is one or more complete (circular) genomes, using the median of their sizes; two if not, using the maximum size of the bins with least number of contigs

An intriguing observation was that nine clusters and four singletons of these viruses were spatially proximal to crAssphage and to each other in the scatter plots (Fig. 7a). Their sizes fall within a small range (84–106 kb). The ANI plot shows that some within the clusters share high degrees of sequence similarity (Fig. 7b). These new viral genomes may represent a related super group of viruses. We have coined them “crish” phages.

The putative viral genomes constitute large proportions within multiple metagenomes (Table 3); nevertheless, in five samples, we were only able to assemble a single viral genome at less than 1% abundance (10, 76, 538, 6128, and 80152). The highest total viral abundance, 59.2%, was observed in the HT sample 560. This includes a single putative virus (ph10) mapped by 39.1% reads of the entire metagenome. Significantly, the seven healthy controls have an overall higher abundance of putative viruses (29.0 ± 18.1%) than the 22 patients (8.2 ± 13.6%) (one-tailed *t* test *p* value = 0.011).

## Conclusions

The main etiological finding of this work is that diarrheal disease caused by bacterial pathogens might be polymicrobial. This concurs with PCR-based findings that revealed the presence of co-infections in pediatric diarrhea from the Global Enteric Multicenter Study (GEMS) [69] and in travelers’ diarrhea in West Africa [70] and Mexico, Guatemala, and India [9]. Until recently, the prevailing paradigm has been that in most cases of infectious disease, only a single bacterial or viral agent was responsible [71]. All three studies used PCR directed against only one or two virulence genes and/or ribosomal RNA genes to identify pathogens; some of the virulence genes are plasmid-encoded. While the GEMS study was the most quantitative of the three, none were capable of providing the type of genomic detail that we present here.

A limitation is that our pathogen classification methods were often applied to incomplete genomes, so it is likely that we missed annotation of virulence genes or complete pathogenicity islands or, for example, parts of pathways for synthesis of O antigens and flagella. Thus, the analysis provided in Additional file 1: Tables S8 and S11 is only partially predictive of the type of pathogen identified. We recovered only three plasmid bins, two of which carried predicted virulence factor-encoding genes (Additional file 1: Table S8); this underrepresentation may occur because plasmid elements commonly carry repetitive elements that break assemblies. Retrospectively, we also appreciated that the original screens for pathogens and virulence factors were not as robust as that provided by current technologies. We were surprised, however, that our assays did not detect the Shiga toxin genes in *E*. *coli* strains 6163 (a) and 678 (a).

Metagenomic sequencing revealed that some TD samples contained up to three different potentially *E*. *coli* genomes, all with distinct virulence profiles, while other samples carried mixtures of other members of the Enterobacteriaceae with unknown roles in pathogenesis but carrying genes encoding clinically important antimicrobial resistance. We also identified new dark matter genomes, one potentially pathogenic. One must be cautious, however, to state whether the presence of an organism or a virulence gene is indeed the true cause of disease. Future studies are needed to establish whether virulence genes are actually being expressed and whether low abundance organisms with pathogen signatures are contributing to virulence. As mentioned previously, the concept of microbial succession following secretory diarrhea also needs to be considered, particularly when a pathogen is found at a low copy number. In our study, we had limited information about the time of sample collection following onset of disease so it is likely that some were active disease samples and some may have been early-stage recovery samples, as described by David et al. [58].

## Methods

### Study cohort and biological samples

Stool samples were collected from adults who traveled from the USA to Mexico or India between 2005 and 2010 and who developed TD or who remained diarrhea-free (healthy travelers, HT). TD is clinically defined as the passage of three or more unformed stools within a 24-h period accompanied by at least one other enteric symptom, such as abdominal cramps, vomiting, nausea, and fever. The disease is usually self-limiting and resolves within four days [72]. The cohort and sample collection is described in a previous publication and as follows [12]. The subjects were adult males and non-pregnant females who traveled from the USA to Mexico (28) or India (2) and who either developed TD (23) or remained healthy (7, HT) (Additional file 1: Table S1). Subjects lived with host families at the destinations. Exclusion criteria are described in [12], but most importantly subjects were excluded if they had taken antibiotics, had gastroenteritis, or a history of inflammatory bowel disease. Samples were self-collected within 72 h of acute disease onset and were transported to study investigators on site within 30 min of collection, where they were aliquoted and stored at −80 °C. The samples were sent to the University of Texas Health Science Center (UTHSC) within 24 h of collection. Stool samples from healthy, diarrhea-free travelers in Mexico were collected as controls (DuPont, personal communication). No demographic data, except collection site and diarrheal state, were available to us due to de-identification and processing when the samples were originally received at Baylor College of Medicine.

At UTHSC, stool samples were screened for the presence of the following pathogens as described in [73–75]: *Aeromonas* sp., *Campylobacter* sp., enterotoxigenic *E*. *coli* (ETEC), diffusely adherent *E. coli*, *Plesiomonas* sp., *Salmonella* sp., *Shigella* sp., *Vibrio* sp., *Yersinia* sp., *Cryptosporidium* sp., *Entamoeba histolytica*, *Giardia lamblia*, adenovirus, rotavirus, and Norovirus, as previously described [12]. Samples were also screened for the presence of the ETEC, ST, and LT toxins, by PCR, also as previously described [12, 76]. All of the samples used in this study tested negative for all pathogens and toxins tested.

### DNA extraction and BFT PCR assays

Genomic DNA was extracted from 0.5 g of stool per subject. Lysis buffer (5 mL, Perkin Elmer 852) was added to each stool sample and vortexed until homogenous. Homogenized sample (1.2 mL) and Proteinase K enzyme (15 μM, Sigma Aldrich, PN. P2308) enzyme was aliquoted to a 2 mL tube with garnet beads (Mo Bio PN. 12830-50-BT). Bead tubes with 1.2 mL of specimen were then incubated at 65 °C for 10 min and then at 95 °C for 10 min. Tubes were then placed on a Vortex Genie 2 to perform bead beating for 10 min and the sample subsequently spun in an Eppendorf Centrifuge 5424 at maximum speed. Supernatant (700 μL) was then transferred to a deep well block. DNA extractions and purifications were performed using a Chemagic MSM I (Perkin Elmer) following the manufacturer’s protocol. Samples were then further purified using the Onestep Inhibitor Removal kit following manufacturer’s instructions (Zymo Research PN. D6035). DNA samples were then quantified using Quant-iT on an Eppendorf AF2200 plate reader. Samples were assessed for DNA integrity by agarose gel electrophoresis. Only non-degraded samples with high molecular weight DNA were used for sequencing.

DNAs were tested for the presence of the *B*. *fragilis* toxin by PCR using the primers BFTF_169: 5′-GCG ACA CAA CTT AAC GAT GTA TCG G-3′ and BFTR_306: 5′-GGT AGA ATC CTT GTC CCT GCC G-3′ that lie within the coding portion of the gene. PCR was performed in a 25 μL reaction containing 2.5 μL 10X buffer, 2.5 μL 50 mM MgCl_2_, 0.75 μL 10 mM dNTPs, 10 μM each primer, 1 μL template DNA diluted 1:10, and 0.1 μL Platinum *Taq* DNA Polymerase (ThermoFisher Scientific, Waltham, MA). Positive controls were performed using 100 ng enterotoxigenic *B. fragilis* chromosomal DNA (gift of Cynthia Sears, Johns Hopkins University, MD). Additional positive controls were run using the universal 16S rRNA gene primers 27F and 1492R [77] and negative controls contained no template. Cycling conditions were 94 °C for 3 min followed by 30 cycles of 45 s at 94°, 30 s at 52°, and 30 s at 72°, followed by 10 min at 72 °C. Products were analyzed on a 2% agarose gel.

### 16S rRNA gene sequencing and analysis

The dual-index sequencing strategy [78] was used to target 16S rRNA gene variable region 4 (V4, approx. 252–253 bp). Pooled amplicons were sequenced on an Illumina MiSeq sequencer at the J. Craig Venter Institute (JCVI) Sequencing Core to yield a total of 1.0 million bases of 250 nt reads. The sequencing quality and the contamination level were assessed by adding a positive control (HMP mock community version 5, BEI catalog # HM-276D) and a negative control (sterile water). We used mothur 1.35.1 [79] to analyze the 16S rRNA gene sequencing data, following the MiSeq standard operating procedures (www.mothur.org/wiki/MiSeq_SOP). In summary, de-multiplexed merged paired-end reads longer than 275 nt, with ambiguous bases, or with more than eight units of homopolymers were discarded. Sequences were aligned to the SILVA SSU database release 123. Chimeric sequences were identified by UCHIME [80] and discarded. A pairwise distance matrix of the aligned sequences was computed. Operational taxonomic units (OTUs) were inferred based on the 97% sequence identity threshold. Taxonomy was assigned using the native Bayesian classifier [81] to search against the Ribosomal Database Project Release 10 [82]. Sequences assigned as Chloroplast, Mitochondria, Archaea, Eukaryota, or unknown organisms were removed. Alpha diversity was evaluated by calculating the Chao 1 index, the inverse Simpson index, and the Simpson’s Equitability index. Beta diversity was measured using the Yue & Clayton estimator [76]. Based on the resulting distance matrix, principal coordinates analysis (PCoA) was performed to visualize the clustering pattern of microbial communities.

### Shotgun metagenome sequencing

Paired-end DNA libraries with insertion size of 350 bp were prepared using the NexteraXT library preparation kit (Illumina, San Diego, CA). Paired-end sequencing was conducted using an Illumina NextSeq 500 sequencer at JCVI with 150 bp read length. One sample, 50012, was dropped from the WGS sequencing due to inadequate DNA quantity. The remaining 29 samples were divided into two batches: 10 samples were sequenced to yield an average of ten Gb, and the remaining samples were sequenced to three Gb. Reads were pre-processed following the standard protocol in the JCVI Sequencing Core then further processed using Trimmomatic 0.33 [83] with recommended parameters to remove short, low-quality, and adapter-contaminated reads. To eliminate human reads, data were then mapped to the Human Reference Genome Release 107 (Genome Reference Consortium) using Bowtie2 v2.2.5 [84] with default parameters. Mapped human reads were discarded from the pool.

### De novo assembly and binning

Processed paired-end WGS reads were subject to de novo metagenome assembly using IDBA-UD 1.1.1 [32]. Contigs shorter than one kb were dropped from the pool. The quality of assembly was assessed using Quast 2.3 [85] and a series of in-house Python scripts. Original reads were mapped backed to the contigs using Bowtie2, and the read coverage of each contig was calculated using the “genomecov” command implemented in BEDTools v2.24.0 [86]. Circularizable contigs were identified based on the presence of repeated sequences on of both ends using the protocol described in [87].

VizBin v0.9 [35] was used to cluster the contigs based on *k*-mer signature, using default parameters (*k* = 5). Moreover, three automated binners, MaxBin 1.4.5 [88], MetaBat 0.25.4 [89], and Concoct 0.4.0 [90] were executed using default parameters and the resulting binning schemes were mapped to the VizBin outputs (Additional file 2: Figure S5). The scatter plots were visualized using R. Distinct clusters of contigs that likely represent individual genomes (bins) were manually isolated. For multiple closely related genomes that could not be separated by *k*-mer signature, a master bin containing them was first isolated, and its member contigs were then further divided into sub-bins based on the distribution of coverage. The quality of bins was assessed using CheckM 1.0.3, which computes the completeness and contamination of a bin based on the presence of lineage-specific single-copy marker gene sets [36].

### Gene calling and functional annotation

Open reading frames (ORFs) on the assembled contigs were identified and translated into amino acid sequences using Prodigal 2.6.2 [91], with parameters set to target closed ORFs only in metagenomic contigs. SSUs rRNA genes were identified using Metaxa2 v2.0.2 [92]. In addition, the automated annotation pipeline Prokka 1.2 [93] with all optional features enabled generation of NCBI-compatible annotation files.

Several general and specific sequence databases were searched to infer the functional properties of the predicted proteins: Kyoto Encyclopedia of Genes and Genomes (KEGG) (Feb. 2016 release) [94, 95] for general functional annotation and categorization by module and by pathway, Resfams full HMM database v1.2 [96] for antimicrobial resistance genes, VFDB R3 [97] for virulence factors, ACLAME 0.4 [98] for plasmid-related elements, and PHAST (Nov. 2014 release) [99] for virus- and prophage-related sequences.

Proper sequence similarity search tools were chosen to search protein sequences against these databases: NCBI BLASTp 2.2.30+ [100] was used for VFDB, ACLAME, and PHAST, and DIAMOND 0.7.9 [101] was used for KEGG, with search cutoffs set as *E* value ≤ 1e-50 and identity ≥ 50%. HMMER 3.1b2 [102] was used for Resfams, with search cutoffs set as *E* value ≤ 1e-50 and coverage ≥ 80%. Search results were processed using in-house Python scripts to retain up to one hit per query protein per category. The relative abundance of each functional category was calculated as the sum of (ORF length × contig coverage) divided by the sum of (ORF length × contig coverage) of the entire metagenome.

Serotypes of *E*. *coli* strains (Additional file 1: Table S9) were predicted in silico with assembled contigs using a tool called SerotypeFinder 1.1 [103] housed on the Center for Genomic Epidemiology (CGE) server (http://cge.cbs.dtu.dk/services/SerotypeFinder/). Similarly, in silico multilocus sequence typing of *E*. *coli* strains (Additional file 1: Table S10) was performed with assembled contigs using the *adk*, *fumC*, *gyrB*, *icd*, *mdh*, *purA*, and *recA* alleles described by Wirth et al. [48] using the MLST 1.8 MultiLocus Sequence Typing tool [104] on the Center for Genomic Epidemiology server (http://cge.cbs.dtu.dk/services/MLST/).

### Comparative genomic analysis

The sequence similarity between bins was measured by the average nucleotide identity (ANI) [105], as computed by pyani 0.1.3 [106], which calls MUMmer 3.23 [107] to align genomes. *E*. *coli* genomes were aligned to one or more complete reference genomes of related taxonomic groups using the progressive Mauve algorithm [108] as implemented in Mauve 2.4.0 [109]. Genomic regions of interest were aligned and highlighted for cross comparison among samples.

### Phylogenomic reconstruction

The phylogenetic relationships of the 39 recovered *E*. *coli* genomes were inferred as follows:. The amino acid sequences of the CheckM-identified, Enterobacteriaceae-specific marker genes (UID5124) in each genome were extracted. Sequences were discarded if multiple copies of a marker gene were present in a genome. Gene families that contain members from at least 75% of the 24 near-complete *E*. *coli* genomes (i.e., 18) were used for phylogenetic reconstruction. A total of 20 complete reference genomes, including 18 *E*. *coli* strains and two outgroups, were added to the analysis. For each gene family, member sequences were aligned and trimmed using GUIDANCE 2.0.1 [110], which calls MAFFT v7.123b [111] for sequence alignment. Results were subject to manual curation to further improve alignment quality. A total of 1032 marker gene families and 45,660 amino acid sequences (14,567,671 aa) were retained. Sites that are polymorphic within the 57 *E*. *coli* taxa were extracted and merged into a master alignment. This resulted in 14,290 sites. ProtTest 3.4 [112] was used to infer the optimal amino acid substitution model for the master alignment, and that model under both LnL and BIC criteria was both JTT + G. The phylogeny was reconstructed using the maximum likelihood method as implemented in RAxML 8.2.8 [113] with the JTT + G model. One hundred rapid bootstraps were executed to provide nodal support metrics.

The same pipeline was used for the reconstruction of phylogenetic tree of the 320 bins representing cellular organisms, using the 56 universal marker gene families (CheckM UID1) totaling 20,300 sites after alignment and quality trimming. A slightly modified pipeline was used for building the phylogenetic tree of the nine TM7 bins plus 11 reference TM7 genomes. Instead of using CheckM-predicted marker genes (which may be less sensitive for the under-characterized lineage TM7), we inferred orthologous groups (OGs) using OrthoMCL 2.0.9 [114] with default parameters. For each resulting OG, multiple copies from the same genome were excluded. A total of 408 filtered OGs with ≥ ten members were included in the subsequent phylogenetics pipeline.

## Additional files

Additional file 1: Supplemental Text. (XLSX 334 kb)

Additional file 2: Tables S1-S11 and Figure Legends. (PDF 1992 kb)

Additional file 3: Figures S1-S10. (DOCX 148 kb)

Additional file 4: *k*-mer signature-based scatter plots with multiple features visualized for all 29 metagenomic assemblies. (ZIP 33507 kb)

## Abbreviations

ANI: Average nucleotide identity
CAG: Co-abundance gene groups
CPR: Candidate phyla radiation
DAEC: Diffusely adherent *Escherichia coli*
EAEC: Enteroaggregative *E. coli*
EHEC: Enterohemorrhagic *E*. *coli*
EIEC: Enteroinvasive *E*. *coli*
EPEC: Enteropathogenic *E*. *coli*
ETEC: Enterotoxigenic *E*. *coli*
ExPEC: Extraintestinal *E*. *coli*
HT: Healthy traveler (control)
JCVI: J. Craig Venter Institute
LEE: Locus of enterocyte effacement
LT: Heat-labile enterotoxin
MLST: Multilocus sequence typing
NMEC: Neonatal meningitis-associated *E*. *coli*
OTU: Operational taxonomic unit
PCR: Polymerase chain reaction
ST: Heat-stable enterotoxin
TD: Traveler’s diarrhea
WGS: Whole genome shotgun
