# Supplementary material for: Visualization-assisted binning of metagenome assemblies reveals potential new pathogenic profiles in idiopathic travelers’ diarrhea
Source: Microbiome. 2018 Nov 8;6:201. doi: 10.1186/s40168-018-0579-0 (PMC6225641; doi:10.1186/s40168-018-0579-0)
Supplement: Supplementary file 2 — Tables S1-S11 and Figure Legends. (PDF 1992 kb) [file 40168_2018_579_MOESM2_ESM.pdf]

Supplemental Figure Legends

Figure S1. Alpha diversity metrics based on 16S rRNA gene data. Rarefied OTU statistics as represented by Chao 1 index for richness, Inverse Simpson Index for diversity and Simpson's Equitability for evenness are displayed. Samples are sorted by Chao 1 index from low to high. Error bars represent 95% high and low confidence intervals. Blue IDs are healthy traveler controls.

Figure S2. WGS-based taxonomic profiles of prokaryotes. Top 30 prokaryotic genera by average relative abundance are displayed. Color temperature represents the $\log_{10}$ relative abundance of reads mapped to each genus.

Figure S3. WGS-based taxonomic profiles of non-phage viruses. Top 50 most abundant virus species whose scientific name does not contain the substring "phage" are displayed. Color temperature represents the $\log_{10}$ relative abundance of sequences mapped to each species.

Figure S4. Functional profiles of the metagenomes. Identified ORFs were assigned to KEGG pathways, which fall into five major and 33 minor categories. Color temperature represents the proportion of genes (ORFs) in each category, as weighted by (gene length × host contig coverage). The assignment results to four specific features (and databases) are attached at bottom. The proportions were calculated in the same way but the color temperature is normalized within each row so as to highlight the cross-sample comparison. The dendrogram is the hierarchical clustering result based on the KEGG pathway categories only.

Figure S5. Comparison of binning results by multiple tools. As exemplified in sample 80129, the binning results of three automated binners: Concoct, MaxBin and MetaBat, are visualized in overlap with the *k*-mer signature-based scatter plot generated by VizBin. Different combinations of color and shape represent contigs assigned to different bins.

Figure S6. Illustration of the major steps of the metagenomic assembly and binning workflow designed and used in this work. Contigs assembled from each DNA sample are displayed in a scatter plot, with multiple properties indicated by different visual elements. Specifically, the x- and y-coordinates are the dimension reduction result computed from *k*-mer signatures. The area, opacity and color indicate the length, coverage and taxonomic assignment of the contig, respectively. Contigs carrying certain features (*e.g.*, SSU gene, virulence factor)

are selectively highlighted (bordered). Clusters of contigs that may represent individual genomes ("bins") were manually selected from the scatter plot. Closely-related genomes were further separated by observing the distribution pattern of certain metrics, such as coverage (shown in inset). The quality of each candidate bin was assessed in downstream analyses (such as CheckM) and human knowledge, which in turn guided the adjustment of the bins.

Figure S7. Cross comparison of recovered *E. coli* draft genomes. The permutation (order) of genomic regions is visualized by colored blocks as aligned using Mauve [108]. Phylogenetically related genomes are placed in close proximity.

Figure S8. Phylogenetic tree of 320 bins representing cellular organisms. The positions of taxa are identical to those in Fig. 6. Taxonomic descriptions (Table S7) are marked at tips. Branch lengths are drawn to scale. Black and blue lines represent branches with Bootstrap support ≥ and <75 (out of 100), respectively.

Figure S9. Phylogenetic tree of recovered and reference TM7 genomes. TM7x-like strains are in green and the novel TM7z strain is in orange. Bootstrap support values (out of 100) are labeled at nodes. The top left inset shows the two TM7 bins in the scatter plot of contigs in sample 50070.

Figure S10. Distribution of the taxonomic assignment rates of the 163 putative viral genomes. Bin names are omitted. The 17 crAssphage genomes and the three other identified singleton viral genomes are colored. The rest of the bins cannot be assigned to any reference viral genomes. The *y*-axis is scaled to the square root of the actual percentage.

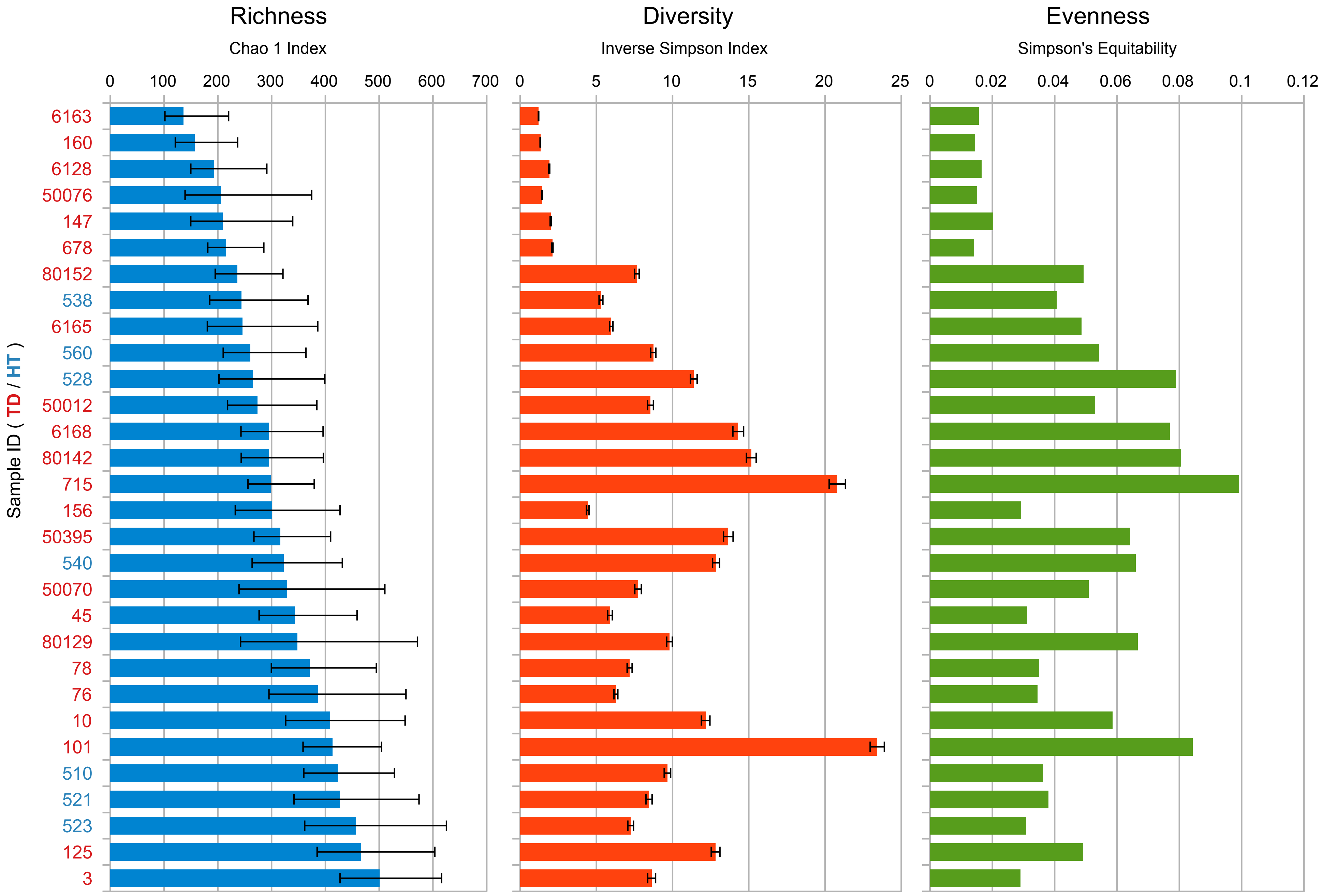
Richness
Chao 1 Index
Diversity
Inverse Simpson Index
Evenness
Simpson's Equitability
Sample ID (TD / HT)
0
100
200
300
400
500
600
700
0
5
10
15
20
25
0
0.02
0.04
0.06
0.08
0.1
0.12
6163
160
6128
50076
147
678
80152
538
6165
560
528
50012
6168
80142
715
156
50395
540
50070
45
80129
78
76
10
101
510
521
523
125
3

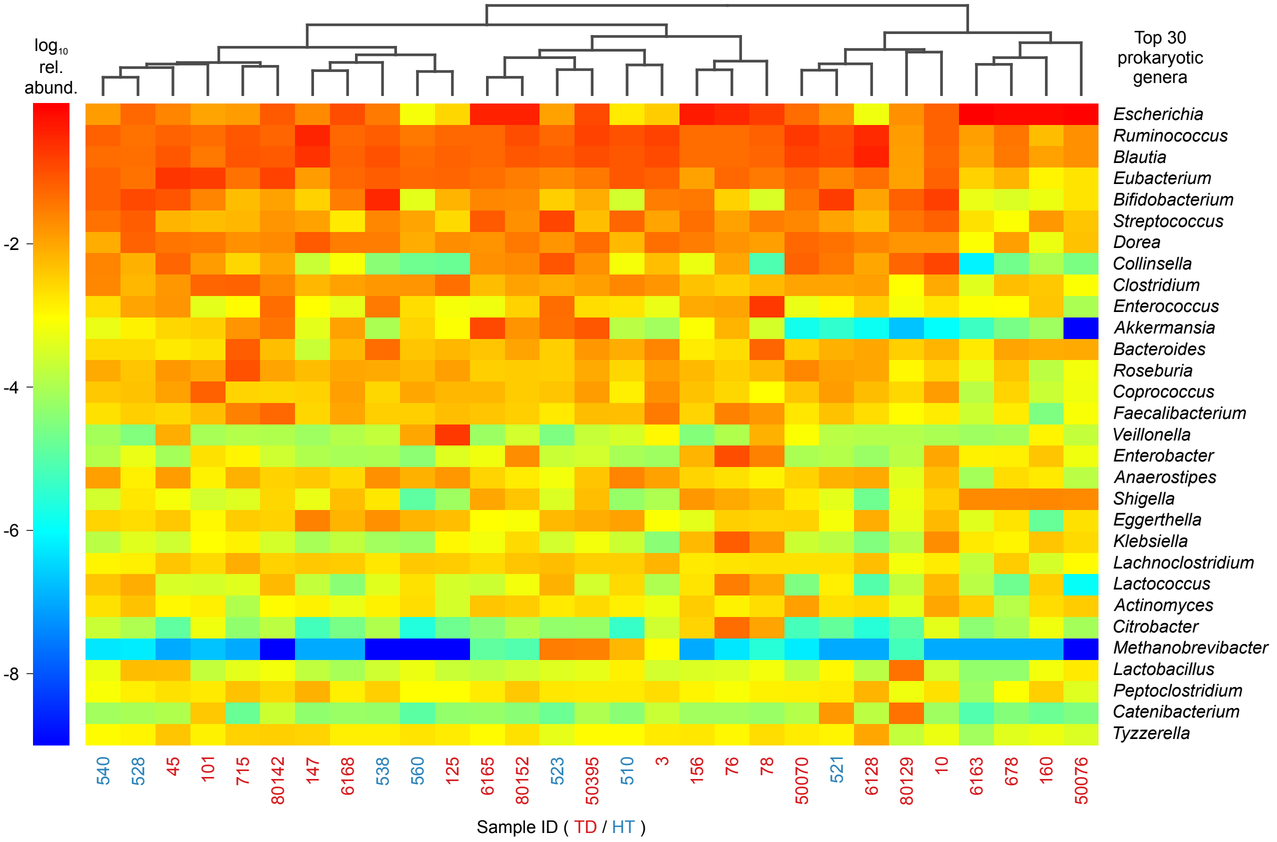

log10 rel. abund.
-2
-4
-6
-8
Top 30 prokaryotic genera
Escherichia
Ruminococcus
Blautia
Eubacterium
Bifidobacterium
Streptococcus
Dorea
Collinsella
Clostridium
Enterococcus
Akkermansia
Bacteroides
Roseburia
Coprococcus
Faecalibacterium
Veillonella
Enterobacter
Anaerostipes
Shigella
Eggerthella
Klebsiella
Lachnoclostridium
Lactococcus
Actinomyces
Citrobacter
Methanobrevibacter
Lactobacillus
Peptoclostridium
Catenibacterium
Tyzzerella
540
528
45
101
715
80142
147
6168
538
560
125
6165
80152
523
50395
510
3
156
76
78
50070
521
6128
80129
10
6163
678
160
50076
Sample ID (TD / HT)

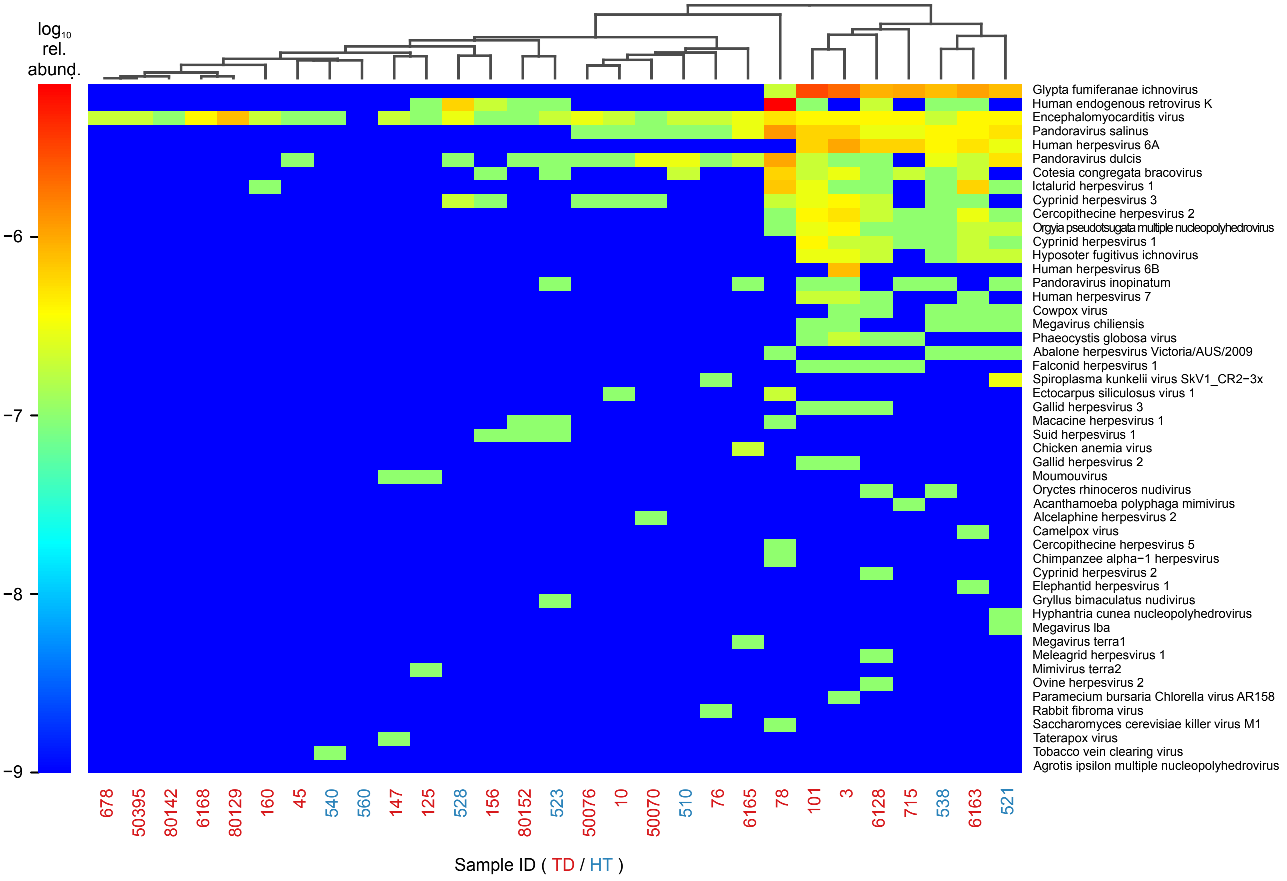

log10 rel. abund.
−6
−7
−8
−9
Glypta fumiferanae ichnovirus
Human endogenous retrovirus K
Encephalomyocarditis virus
Pandoravirus salinus
Human herpesvirus 6A
Pandoravirus dulcis
Cotesia congregata bracovirus
Ictalurid herpesvirus 1
Cyprinid herpesvirus 3
Cercopithecine herpesvirus 2
Orgyia pseudotsugata multiple nucleopolyhedrovirus
Cyprinid herpesvirus 1
Hyposoter fugitivus ichnovirus
Human herpesvirus 6B
Pandoravirus inopinatum
Human herpesvirus 7
Cowpox virus
Megavirus chiliensis
Phaeocystis globosa virus
Abalone herpesvirus Victoria/AUS/2009
Falconid herpesvirus 1
Spiroplasma kunkelii virus SkV1_CR2−3x
Ectocarpus siliculosus virus 1
Gallid herpesvirus 3
Macacine herpesvirus 1
Suid herpesvirus 1
Chicken anemia virus
Gallid herpesvirus 2
Moumouvirus
Oryctes rhinoceros nudivirus
Acanthamoeba polyphaga mimivirus
Alcelaphine herpesvirus 2
Camelpox virus
Cercopithecine herpesvirus 5
Chimpanzee alpha−1 herpesvirus
Cyprinid herpesvirus 2
Elephantid herpesvirus 1
Gryllus bimaculatus nudivirus
Hyphantria cunea nucleopolyhedrovirus
Megavirus lba
Megavirus terra1
Meleagrid herpesvirus 1
Mimivirus terra2
Ovine herpesvirus 2
Paramecium bursaria Chlorella virus AR158
Rabbit fibroma virus
Saccharomyces cerevisiae killer virus M1
Taterapox virus
Tobacco vein clearing virus
Agrotis ipsilon multiple nucleopolyhedrovirus
678
50395
80142
6168
80129
160
45
540
560
147
125
528
156
80152
523
50076
10
50070
510
76
6165
78
101
3
6128
715
538
6163
521
Sample ID (TD / HT)

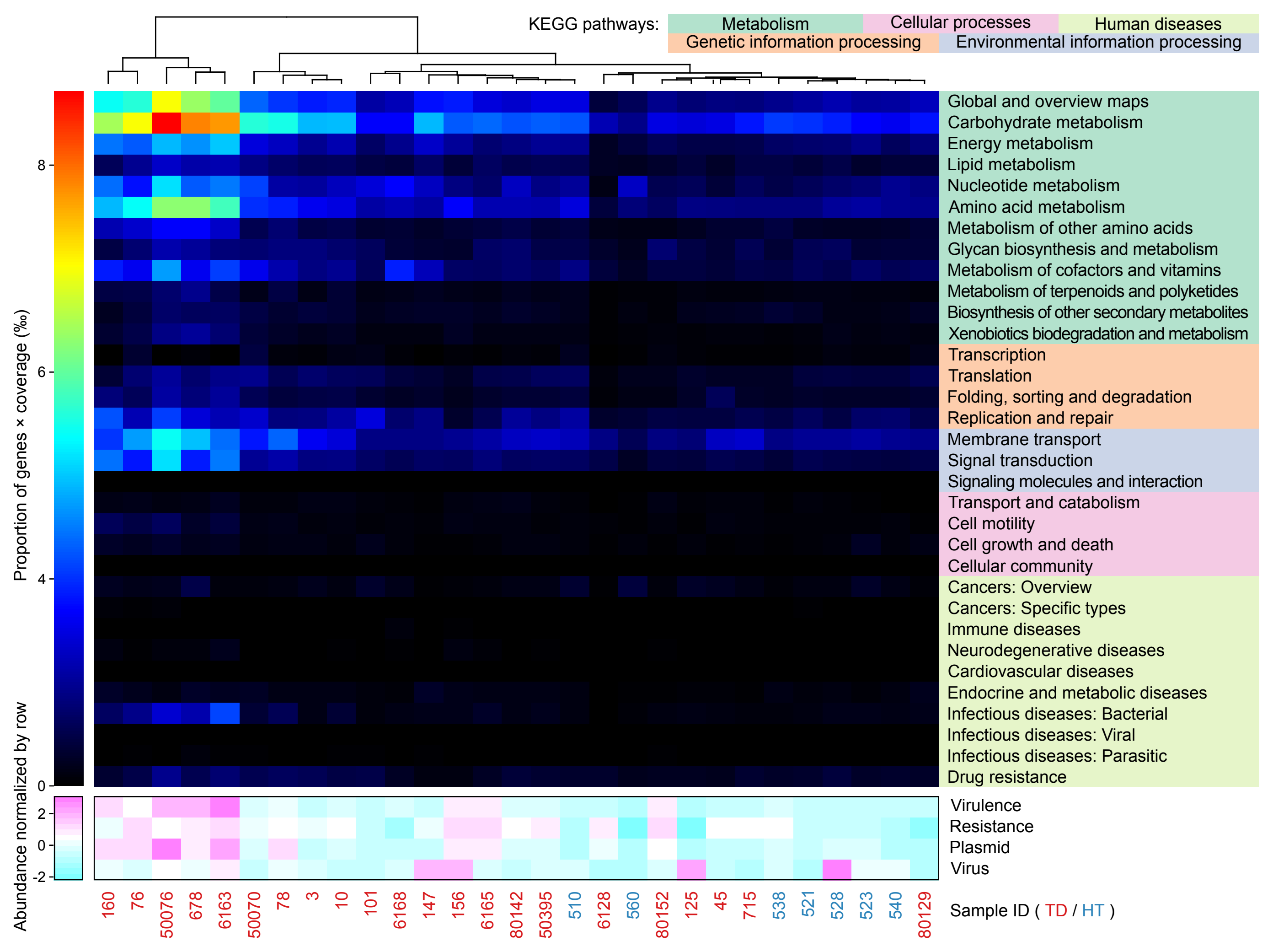

KEGG pathways:
Metabolism
Cellular processes
Human diseases
Genetic information processing
Environmental information processing
Global and overview maps
Carbohydrate metabolism
Energy metabolism
Lipid metabolism
Nucleotide metabolism
Amino acid metabolism
Metabolism of other amino acids
Glycan biosynthesis and metabolism
Metabolism of cofactors and vitamins
Metabolism of terpenoids and polyketides
Biosynthesis of other secondary metabolites
Xenobiotics biodegradation and metabolism
Transcription
Translation
Folding, sorting and degradation
Replication and repair
Membrane transport
Signal transduction
Signaling molecules and interaction
Transport and catabolism
Cell motility
Cell growth and death
Cellular community
Cancers: Overview
Cancers: Specific types
Immune diseases
Neurodegenerative diseases
Cardiovascular diseases
Endocrine and metabolic diseases
Infectious diseases: Bacterial
Infectious diseases: Viral
Infectious diseases: Parasitic
Drug resistance
Virulence
Resistance
Plasmid
Virus
Proportion of genes × coverage (‰)
8
6
4
0
Abundance normalized by row
2
0
-2
160
76
50076
678
6163
50070
78
3
10
101
6168
147
156
6165
80142
50395
510
6128
560
80152
125
45
715
538
521
528
523
540
80129
Sample ID (TD / HT)

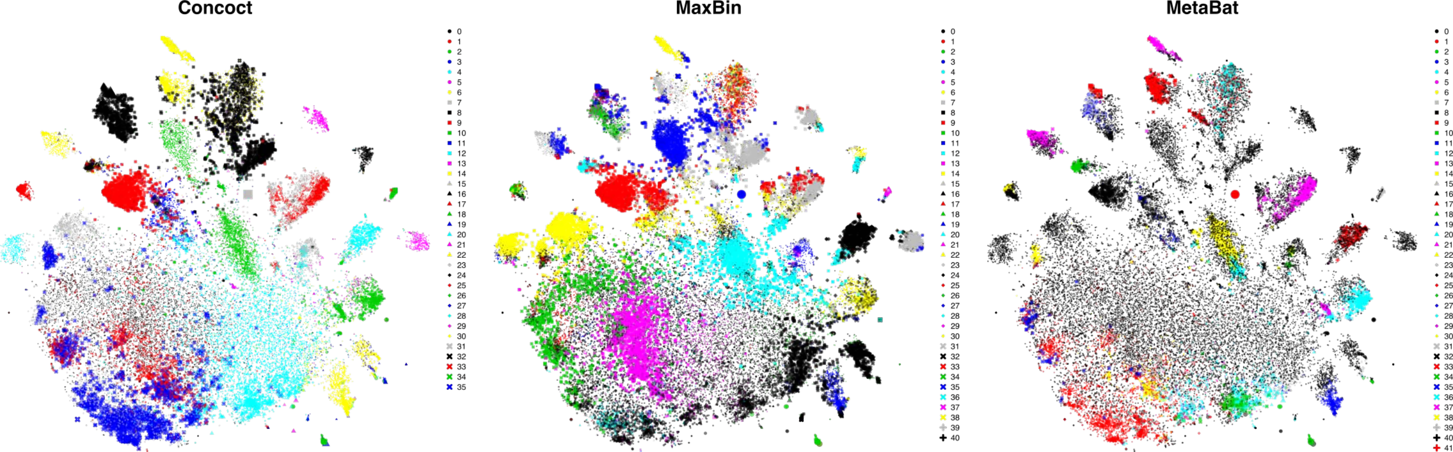
Concoct
0
1
2
3
4
5
6
7
8
9
10
11
12
13
14
15
16
17
18
19
20
21
22
23
24
25
26
27
28
29
30
31
32
33
34
35
MaxBin
0
1
2
3
4
5
6
7
8
9
10
11
12
13
14
15
16
17
18
19
20
21
22
23
24
25
26
27
28
29
30
31
32
33
34
35
36
37
38
39
40
MetaBat
0
1
2
3
4
5
6
7
8
9
10
11
12
13
14
15
16
17
18
19
20
21
22
23
24
25
26
27
28
29
30
31
32
33
34
35
36
37
38
39
40
41

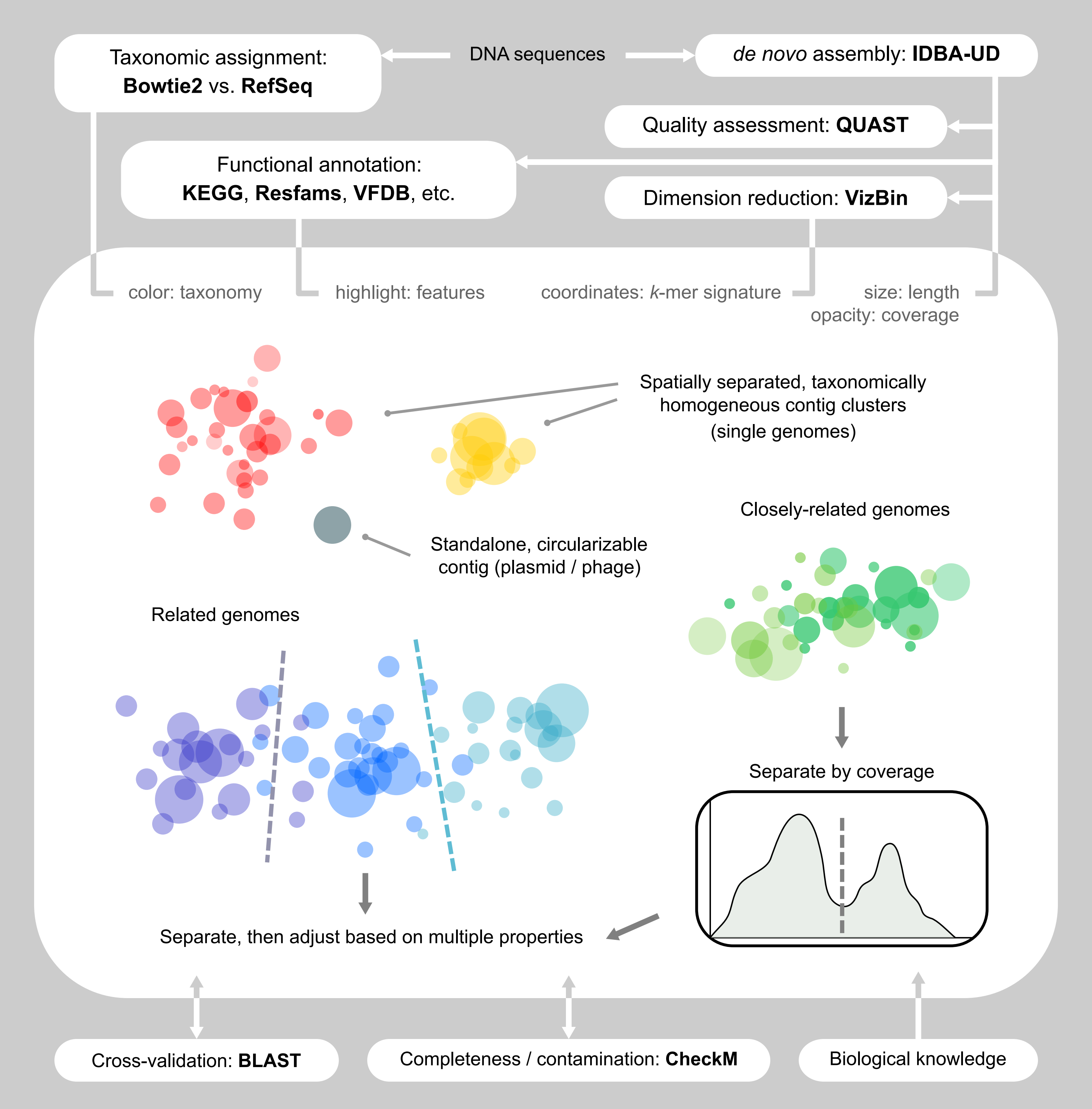

Taxonomic assignment:
Bowtie2 vs. RefSeq
DNA sequences
de novo assembly: IDBA-UD
Quality assessment: QUAST
Functional annotation:
KEGG, Resfams, VFDB, etc.
Dimension reduction: VizBin
color: taxonomy
highlight: features
coordinates: k-mer signature
size: length
opacity: coverage
Spatially separated, taxonomically homogeneous contig clusters (single genomes)
Closely-related genomes
Standalone, circularizable contig (plasmid / phage)
Related genomes
Separate by coverage
Separate, then adjust based on multiple properties
Cross-validation: BLAST
Completeness / contamination: CheckM
Biological knowledge

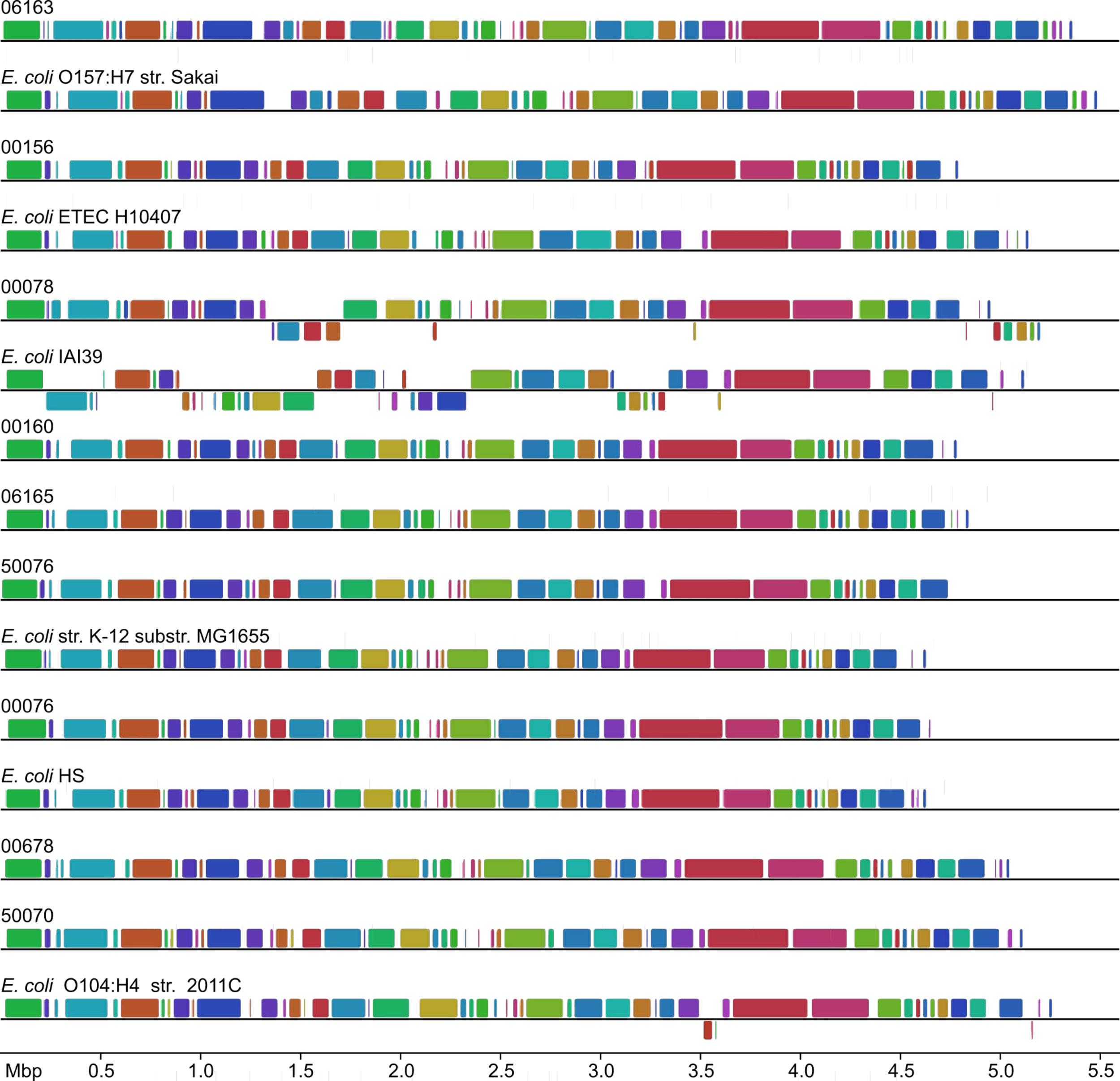
06163
E. coli O157:H7 str. Sakai
00156
E. coli ETEC H10407
00078
E. coli IAI39
00160
06165
50076
E. coli str. K-12 substr. MG1655
00076
E. coli HS
00678
50070
E. coli O104:H4 str. 2011C
Mbp
0.5
1.0
1.5
2.0
2.5
3.0
3.5
4.0
4.5
5.0
5.5

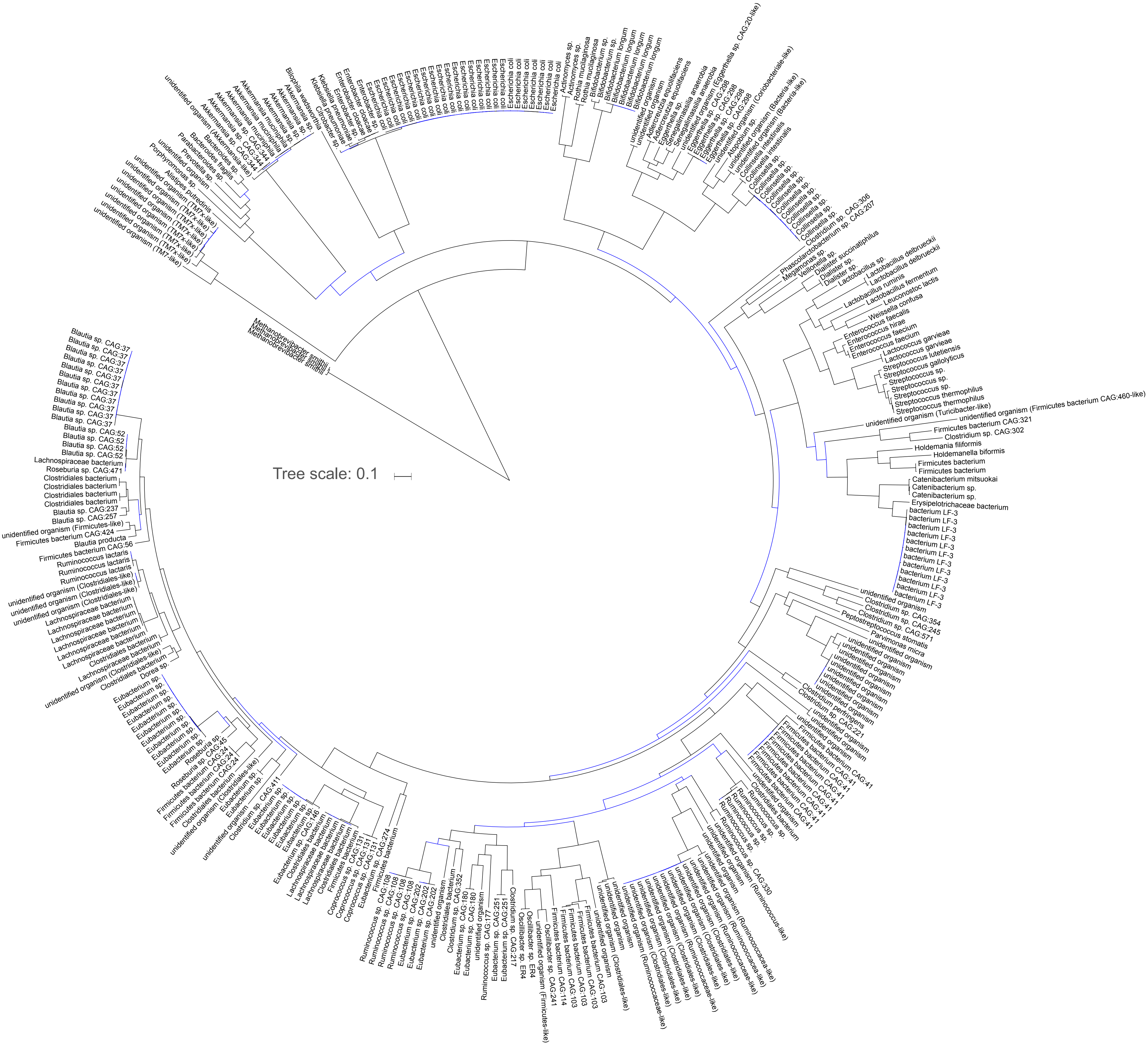

Tree scale: 0.1
Methanobrevibacter smithii
unidentified organism (TM7x-like)
unidentified organism (TM7-like)
Alistipes putredinis
Porphyromonas sp.
unidentified organism
Prevotella sp.
Parabacteroides sp.
Bacteroides fragilis
Bacteroides sp.
unidentified organism (Akkermansia-like)
Akkermansia sp. CAG:344
Akkermansia muciniphila
Akkermansia sp.
Bilophila wadsworthia
Klebsiella pneumoniae
Klebsiella sp.
Citrobacter sp.
Enterobacter sp.
Enterobacter cloacae
Escherichia coli
Actinomyces sp.
Rothia mucilaginosa
Bifidobacterium sp.
Bifidobacterium longum
unidentified organism
Adlercreutzia equolifaciens
Eggerthella sp.
Senegalimassilia anaerobia
unidentified organism (Eggerthella sp. CAG:298-like)
Eggerthella sp. CAG:298
unidentified organism (Coriobacteriales-like)
Atopobium sp.
unidentified organism (Bacteria-like)
Collinsella intestinalis
Collinsella sp.
Clostridium sp. CAG:306
Phascolarctobacterium sp. CAG:207
Phascolarctobacterium succinatiphilus
Megamonas sp.
Veillonella sp.
Dialister succinatiphilus
Dialister sp.
Lactobacillus sp.
Lactobacillus delbrueckii
Lactobacillus ruminis
Lactobacillus fermentum
Leuconostoc lactis
Weissella confusa
Enterococcus faecalis
Enterococcus hirae
Enterococcus faecium
Lactococcus garvieae
Streptococcus lutetiensis
Streptococcus gallolyticus
Streptococcus sp.
Streptococcus thermophilus
unidentified organism (Firmicutes bacterium CAG:460-like)
unidentified organism (Turicibacter-like)
Firmicutes bacterium CAG:321
Clostridium sp. CAG:302
Holdemania filiformis
Holdemanella biformis
Firmicutes bacterium
Catenibacterium mitsuokai
Catenibacterium sp.
Erysipelotrichaceae bacterium
bacterium LF-3
unidentified organism
Clostridium sp. CAG:354
Clostridium sp. CAG:245
Clostridium sp. CAG:571
Peptostreptococcus stomatis
Parvimonas micra
unidentified organism
Clostridium perfringens
Clostridium sp. CAG:221
Firmicutes bacterium CAG:41
Clostridiales bacterium
Ruminococcus sp.
Ruminococcus sp. CAG:330
unidentified organism (Ruminococcus-like)
unidentified organism (Ruminococcaceae-like)
unidentified organism (Clostridiales-like)
unidentified organism
Firmicutes bacterium CAG:103
Firmicutes bacterium CAG:114
Oscillibacter sp. CAG:241
unidentified organism (Firmicutes-like)
Oscillibacter sp. ER4
Clostridium sp. CAG:217
Eubacterium sp. CAG:251
Ruminococcus sp. CAG:177
unidentified organism
Eubacterium sp. CAG:180
Clostridium sp. CAG:352
Clostridiales bacterium
unidentified organism
Eubacterium sp. CAG:202
Ruminococcus sp. CAG:108
Firmicutes bacterium
Eubacterium sp. CAG:274
Coprococcus sp. CAG:131
Firmicutes bacterium
Clostridiales bacterium
Lachnospiraceae bacterium
Eubacterium sp. CAG:146
Eubacterium sp.
Clostridium sp. CAG:411
unidentified organism
Eubacterium sp.
unidentified organism (Clostridiales-like)
Clostridiales bacterium
Firmicutes bacterium CAG:24
Roseburia sp. CAG:45
Roseburia sp.
Eubacterium sp.
Clostridiales bacterium
unidentified organism (Clostridiales-like)
Dorea sp.
Lachnospiraceae bacterium
Clostridiales bacterium
unidentified organism (Clostridiales-like)
Ruminococcus lactaris
Firmicutes bacterium CAG:56
Blautia producta
Firmicutes bacterium CAG:424
unidentified organism (Firmicutes-like)
Blautia sp. CAG:257
Blautia sp. CAG:237
Clostridiales bacterium
Roseburia sp. CAG:471
Lachnospiraceae bacterium
Blautia sp. CAG:52
Blautia sp. CAG:37

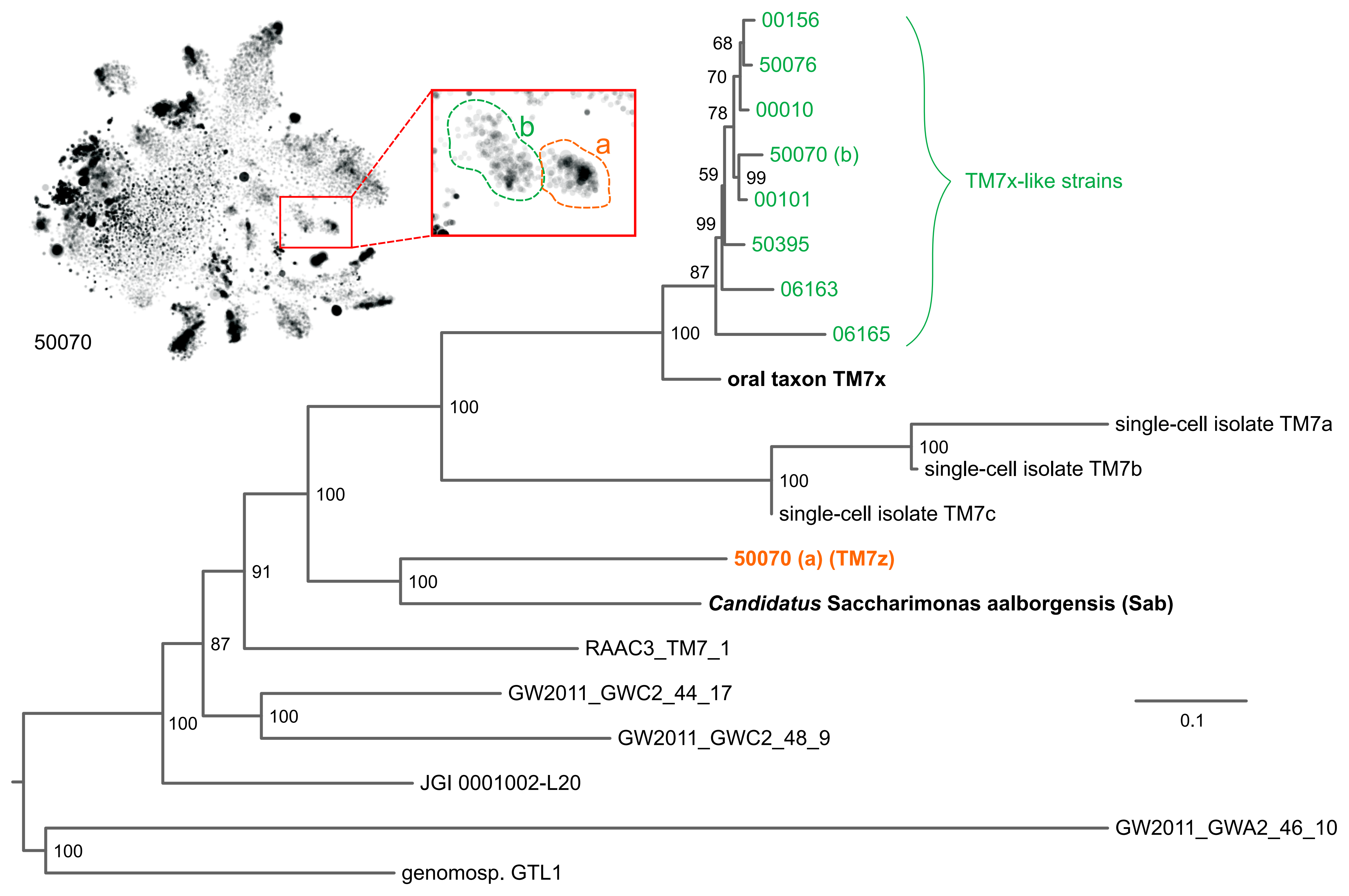

50070
b
a
00156
68
50076
70
00010
78
50070 (b)
59
99
00101
99
50395
87
06163
100
06165
TM7x-like strains
oral taxon TM7x
100
single-cell isolate TM7a
100
single-cell isolate TM7b
100
single-cell isolate TM7c
100
50070 (a) (TM7z)
91
100
Candidatus Saccharimonas aalborgensis (Sab)
87
RAAC3_TM7_1
GW2011_GWC2_44_17
100
100
GW2011_GWC2_48_9
0.1
JGI 0001002-L20
GW2011_GWA2_46_10
100
genomosp. GTL1
