# Supplementary material for: Visualization-assisted binning of metagenome assemblies reveals potential new pathogenic profiles in idiopathic travelers’ diarrhea
Source: Microbiome. 2018 Nov 8;6:201. doi: 10.1186/s40168-018-0579-0 (PMC6225641; doi:10.1186/s40168-018-0579-0)
Supplement: Supplementary file 3 — Figures S1-S10. (DOCX 148 kb) [file 40168_2018_579_MOESM3_ESM.docx]

Supplemental text for manuscript titled “*Visualization-assisted binning of metagenome assemblies reveals potential new pathogenic profiles in idiopathic travelers’ diarrhea*”

**Taxonomic classification of WGS reads, contigs and bins**

WGS sequencing data were classified using Bowtie2 v2.2.5 [1] with default parameters. In order to maximize the taxonomic assignment rate, we built a very large Bowtie2 index database that contains all complete and draft NCBI RefSeq [2] genomes that belong to Bacteria, Archaea, Viruses and Viroids, as of April 2015. Indexing of those genomes took 47.2 days of wall clock time. The database contained 32,852 genomes, representing 48 phyla, 65 classes, 157 orders, 334 families, 1482 genera, and 7668 species. In this manuscript, this protocol is referred to as B2A (Bowtie2 against all). An average mapping ratio of 73.4% was achieved. The result is illustrated in Figs. 1b, S2 and S3.

For comparison, we also classified reads using Kraken 0.10.6 [3] against its “standard” database, built following the program manual in April 2015. This database contains 7,165 bacterial, archaeal and viral genomes). The average mapping ratio was 38.6%. The per-sample mapping ratio is significantly higher by B2A than by Kraken (mean = 2.52, S.D. = 1.37, one-tailed paired *t*-test *p*-value = 1.23e-11) (Table S2).

We cross-compared the taxonomic profiles constructed by different methods (mothur, B2A and Kraken) on different data types (16S rRNA and WGS). At each rank (phylum to genus), profiles were cropped to shared taxonomic terms. They were then normalized to a total count of 1000 per sample. Using mothur 1.35.1 [4], a Bray-Curtis distance matrix was constructed for each profile. The pairwise consistence between distance matrices was assessed by the Mantel test (Table S5). From phylum to family, we observed a consistent trend that B2A and mothur shared the highest congruence, followed by B2A and Kraken, while mothur and Kraken were the least congruent. At genus level the congruence dropped remarkably, a phenomenon also observed in a recent systematic study [5].

Taxonomy assignment of individual contigs or bins was based on the classification of reads mapped to the contig(s). Specifically, at each taxonomic rank, the contig / bin was assigned to a taxon if 75% or more reads were classified under this taxon.

Based on the distribution pattern of taxonomic assignment ratio (Fig. 4), the taxonomic description of bins was determined as follows: Bins with a taxonomic assignment rate above 40% are considered as known organisms. The lowest taxonomic rank with at least 75% recruited reads assigned to it was used to describe the taxonomy of the bin. Bins below 5% were considered as unidentified organisms (“dark matters”). Bins between 5% and 40% were also considered as unidentified organisms, but the taxonomy of the assigned part was appended as “(name)-like” to describe the organism (Table S7).

**References**

1. Langmead B, Salzberg SL: **Fast gapped-read alignment with Bowtie 2.** *Nat Methods* 2012, **9:**357-359.

2. Pruitt KD, Tatusova T, Maglott DR: **NCBI reference sequences (RefSeq): a curated non-redundant sequence database of genomes, transcripts and proteins.** *Nucleic Acids Res* 2007, **35:**D61-65.

3. Wood DE, Salzberg SL: **Kraken: ultrafast metagenomic sequence classification using exact alignments.** *Genome Biol* 2014, **15:**R46.

4. Schloss PD, Westcott SL, Ryabin T, Hall JR, Hartmann M, Hollister EB, Lesniewski RA, Oakley BB, Parks DH, Robinson CJ, et al: **Introducing mothur: open-source, platform-independent, community-supported software for describing and comparing microbial communities.** *Appl Environ Microbiol* 2009, **75:**7537-7541.

5. Sczyrba A, Hofmann P, Belmann P, Koslicki D, Janssen S, Droege J, Gregor I, Majda S, Fiedler J, Dahms E, et al: **Critical assessment of metagenome interpretation − a benchmark of computational metagenomics software.** *bioRxiv* 2017.
