# Supplementary figures and images for: Visualization-assisted binning of metagenome assemblies reveals potential new pathogenic profiles in idiopathic travelers’ diarrhea
Source: Microbiome. 2018 Nov 8;6:201. doi: 10.1186/s40168-018-0579-0 (PMC6225641; doi:10.1186/s40168-018-0579-0)

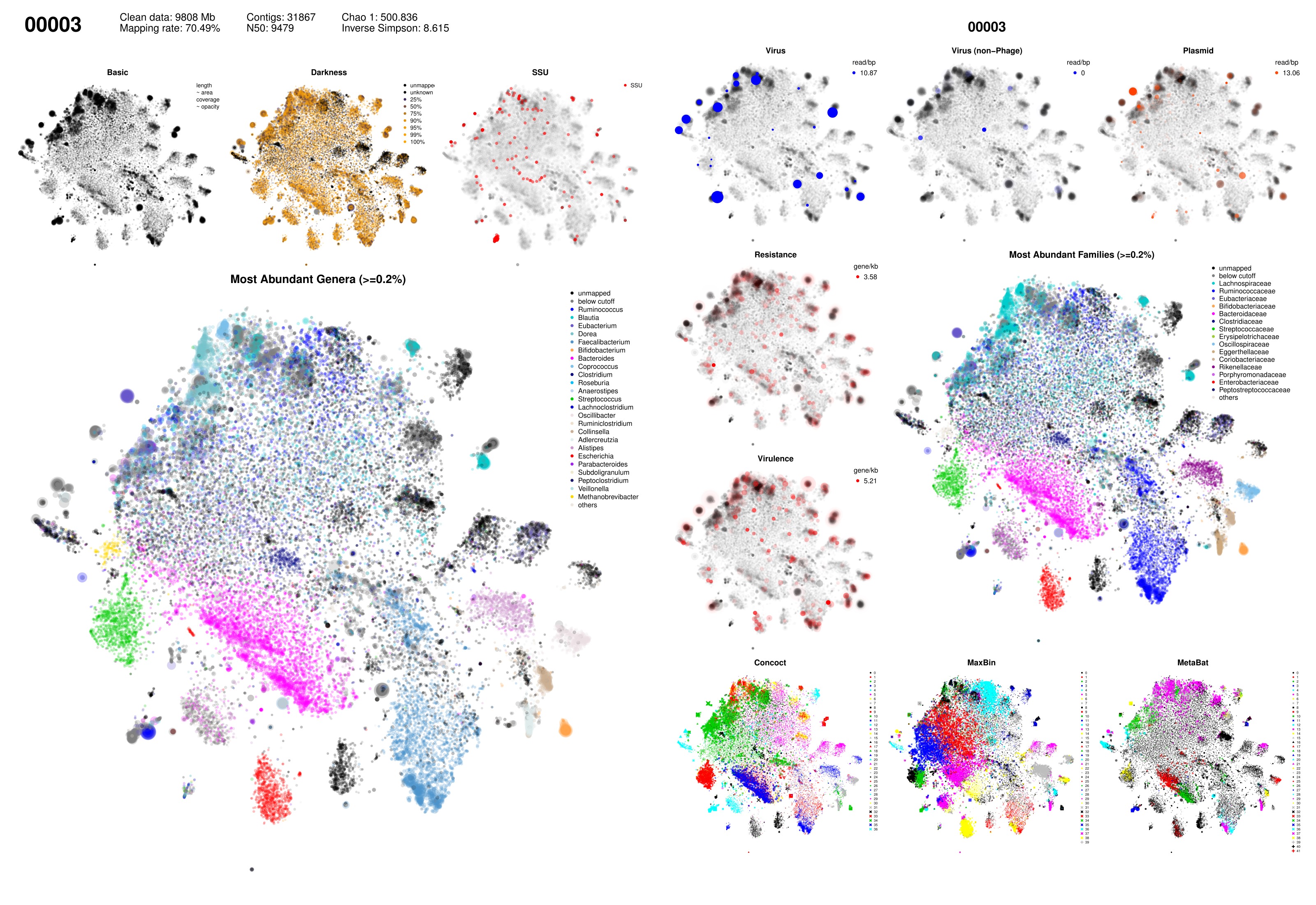

Supplement: Supplementary file 4 — k-mer signature-based scatter plots with multiple features visualized for all 29 metagenomic assemblies. (ZIP 33507 kb) [file 40168_2018_579_MOESM4_ESM.zip › 00003.jpg]

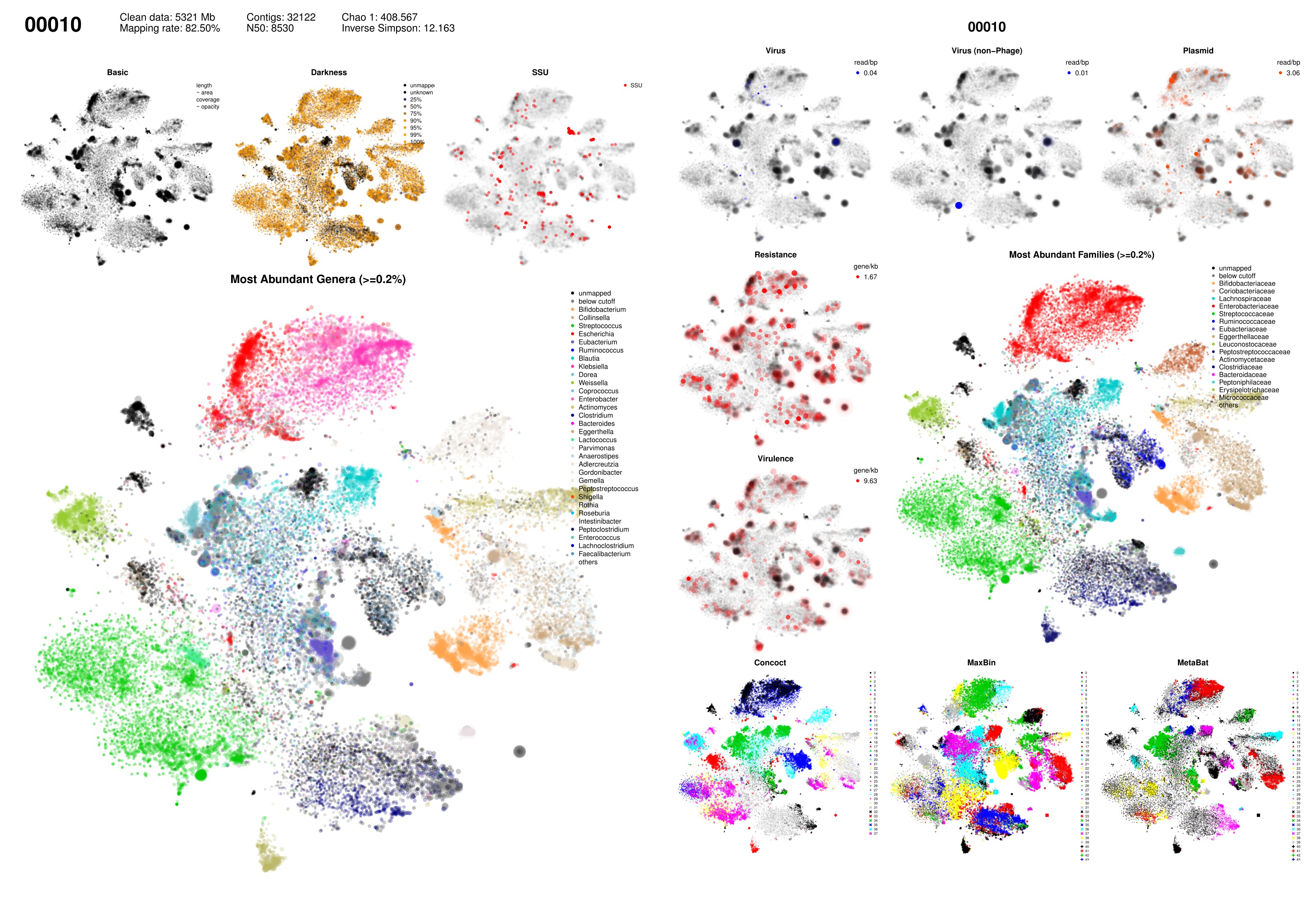

Supplement: Supplementary file 4 — k-mer signature-based scatter plots with multiple features visualized for all 29 metagenomic assemblies. (ZIP 33507 kb) [file 40168_2018_579_MOESM4_ESM.zip › 00010.jpg]

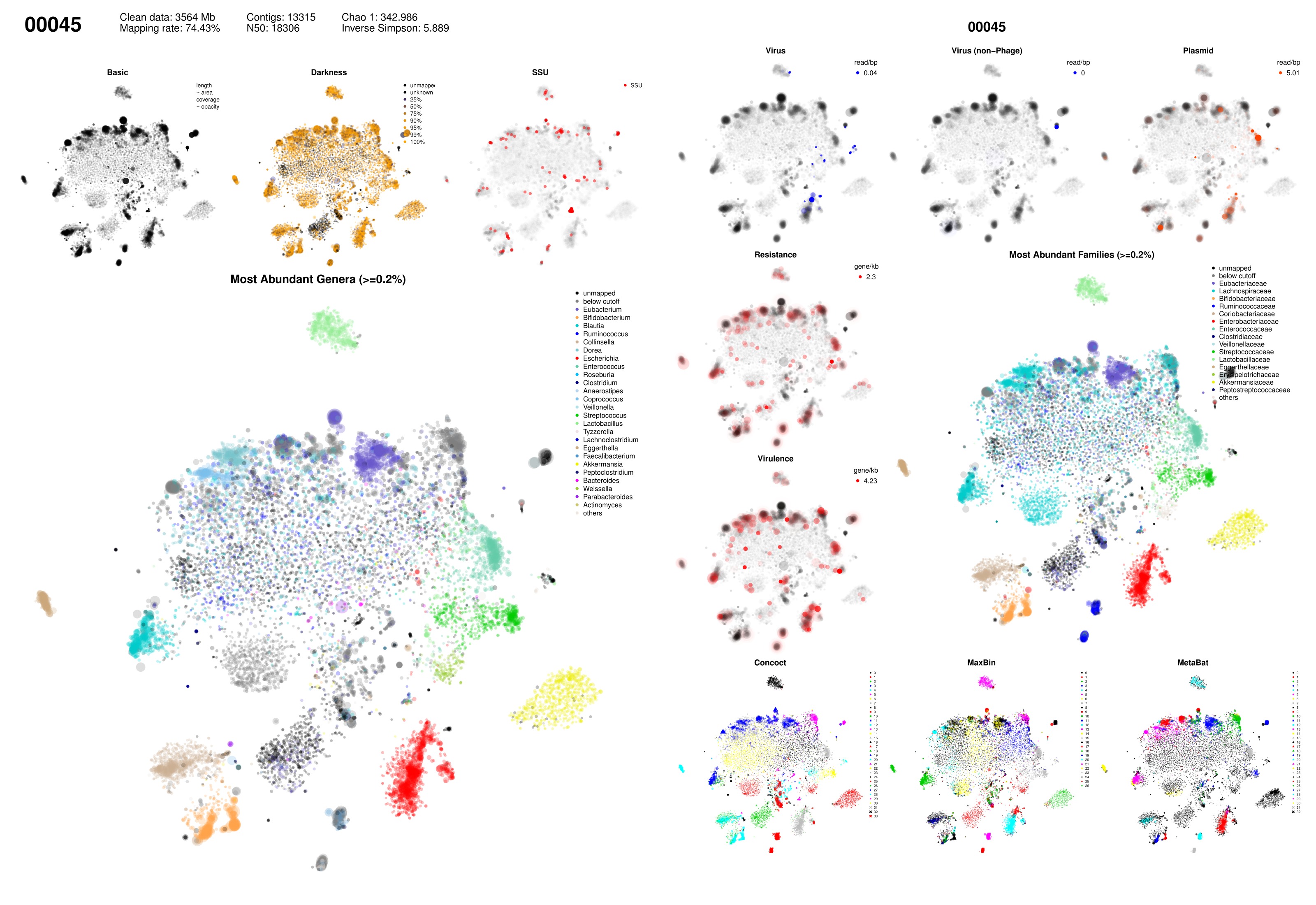

Supplement: Supplementary file 4 — k-mer signature-based scatter plots with multiple features visualized for all 29 metagenomic assemblies. (ZIP 33507 kb) [file 40168_2018_579_MOESM4_ESM.zip › 00045.jpg]

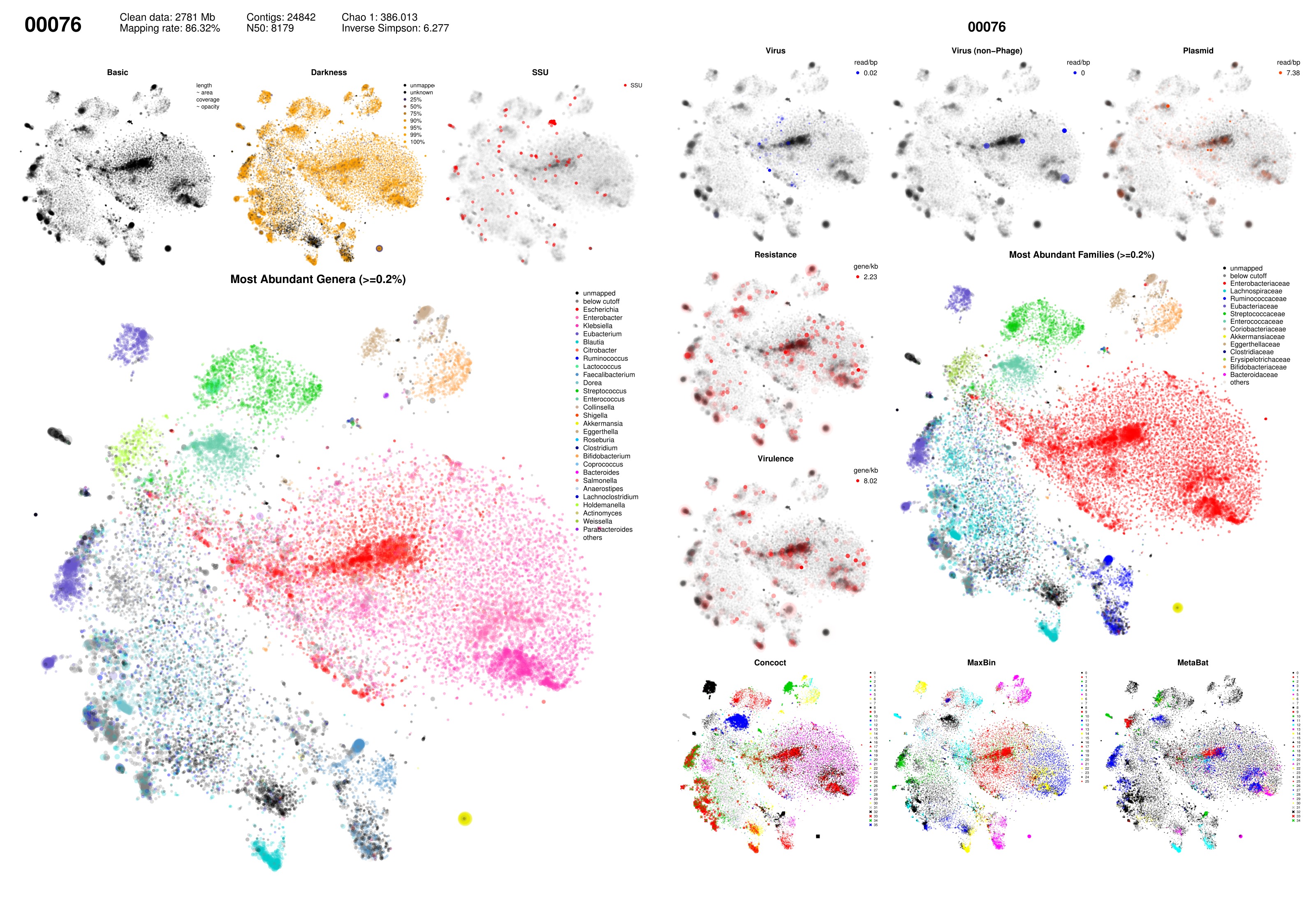

Supplement: Supplementary file 4 — k-mer signature-based scatter plots with multiple features visualized for all 29 metagenomic assemblies. (ZIP 33507 kb) [file 40168_2018_579_MOESM4_ESM.zip › 00076.jpg]

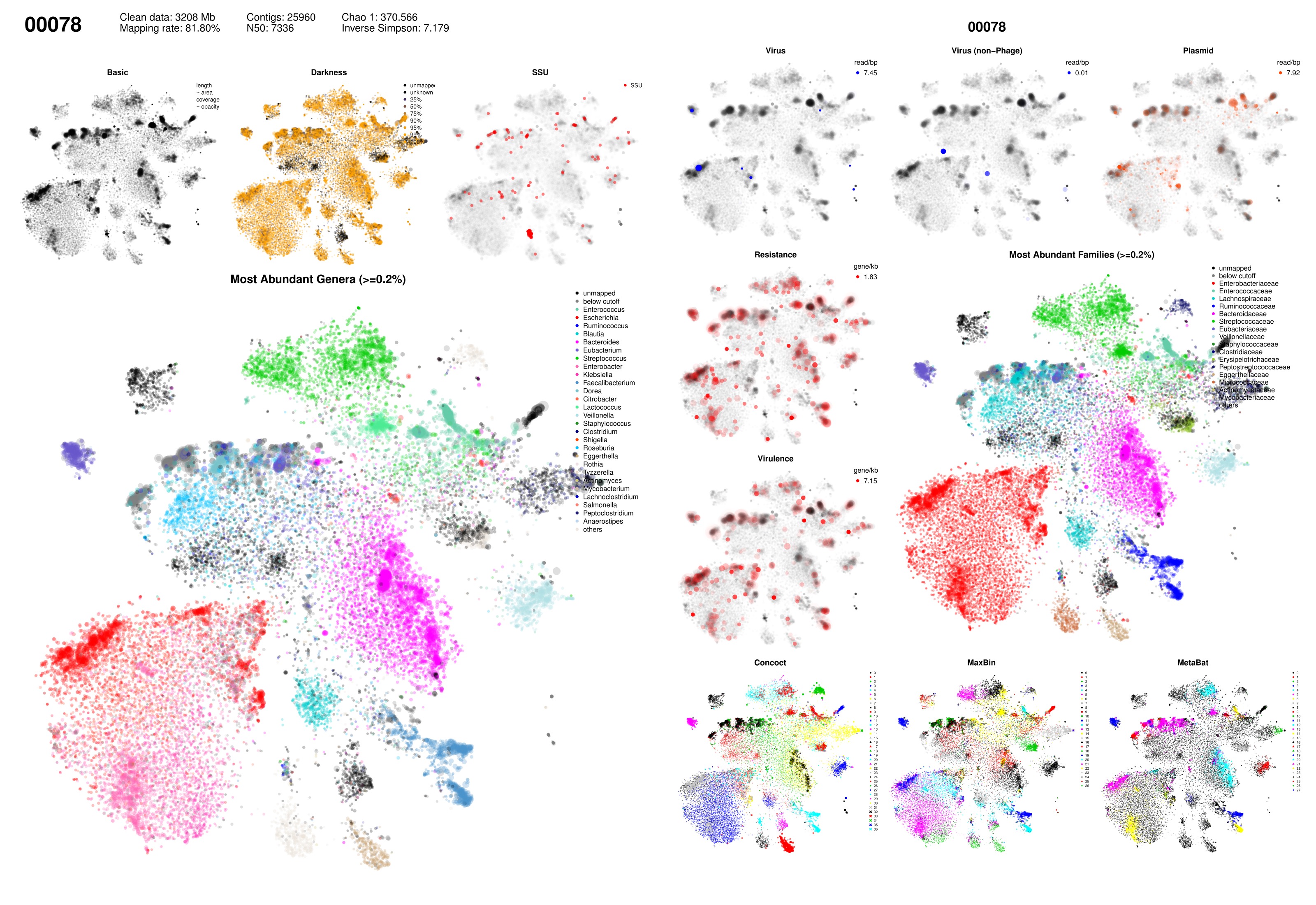

Supplement: Supplementary file 4 — k-mer signature-based scatter plots with multiple features visualized for all 29 metagenomic assemblies. (ZIP 33507 kb) [file 40168_2018_579_MOESM4_ESM.zip › 00078.jpg]

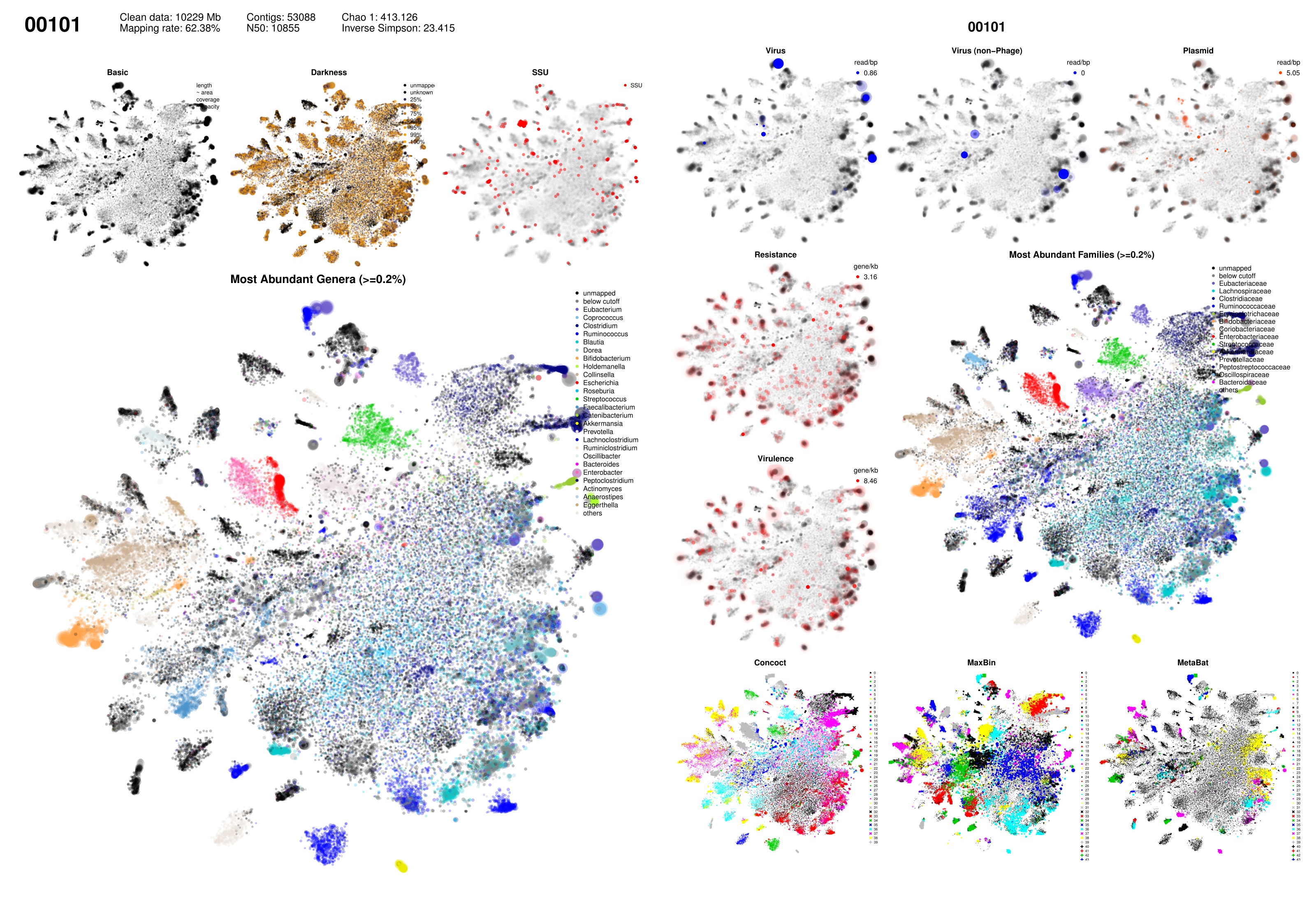

Supplement: Supplementary file 4 — k-mer signature-based scatter plots with multiple features visualized for all 29 metagenomic assemblies. (ZIP 33507 kb) [file 40168_2018_579_MOESM4_ESM.zip › 00101.jpg]

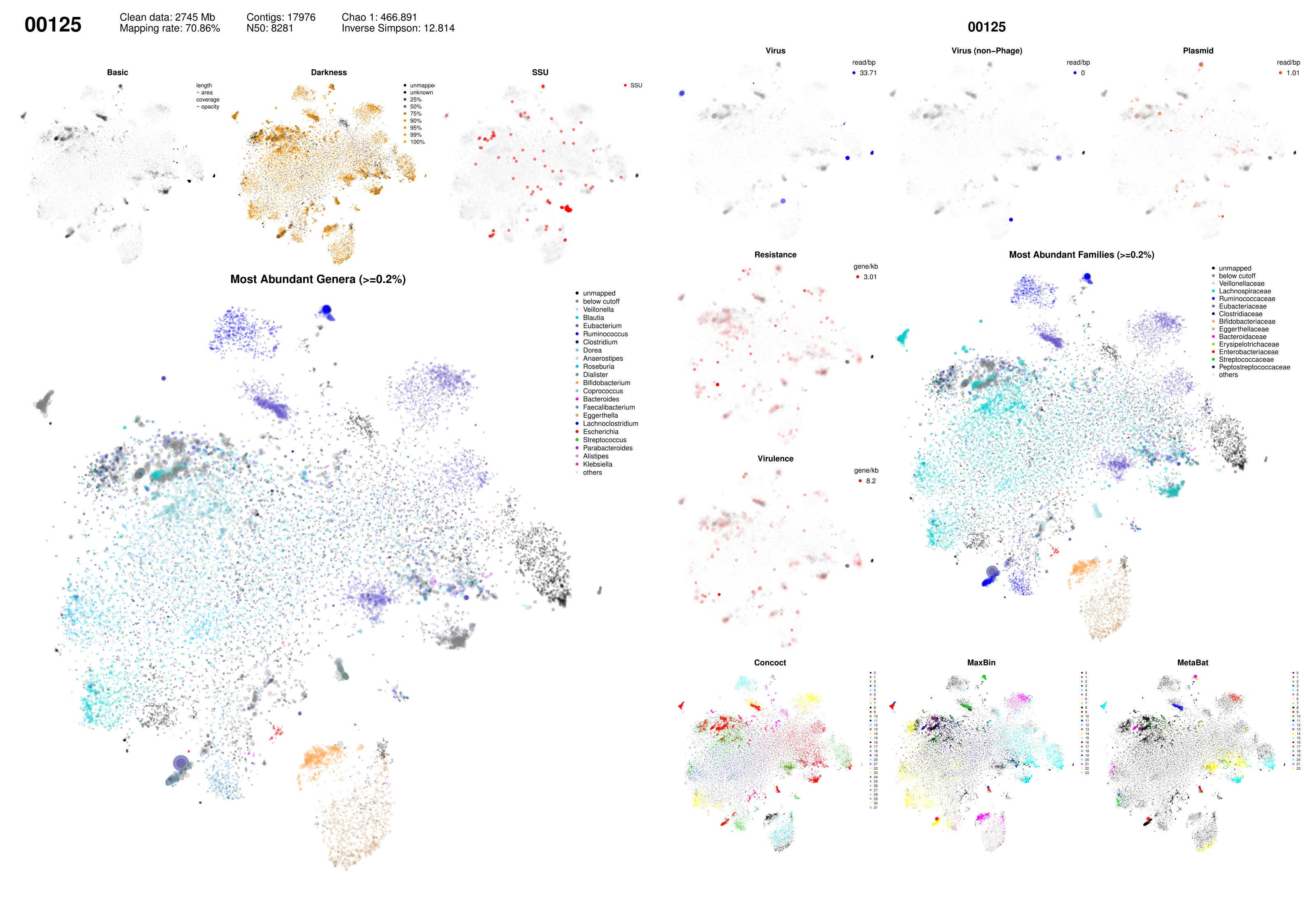

Supplement: Supplementary file 4 — k-mer signature-based scatter plots with multiple features visualized for all 29 metagenomic assemblies. (ZIP 33507 kb) [file 40168_2018_579_MOESM4_ESM.zip › 00125.jpg]

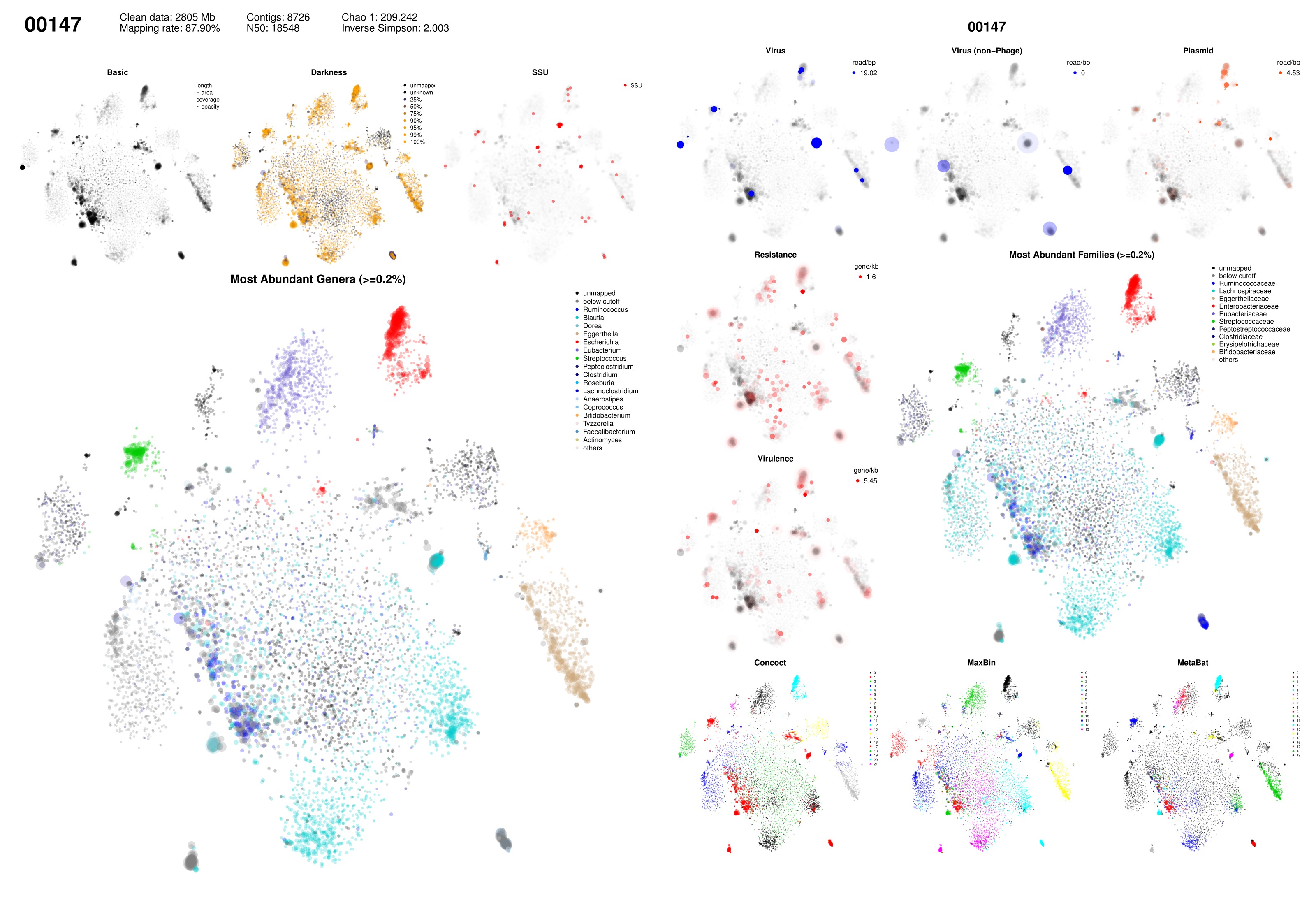

Supplement: Supplementary file 4 — k-mer signature-based scatter plots with multiple features visualized for all 29 metagenomic assemblies. (ZIP 33507 kb) [file 40168_2018_579_MOESM4_ESM.zip › 00147.jpg]

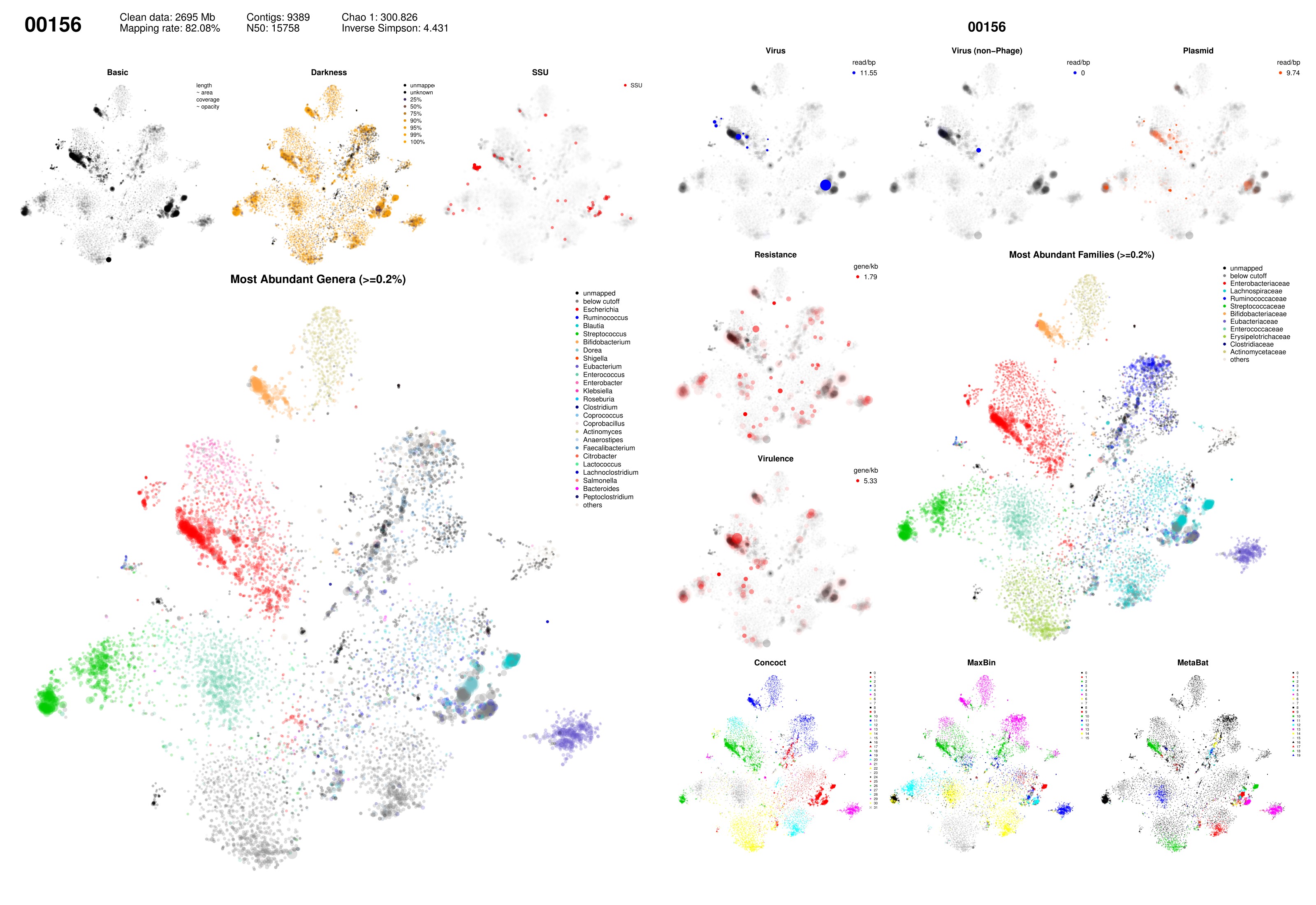

Supplement: Supplementary file 4 — k-mer signature-based scatter plots with multiple features visualized for all 29 metagenomic assemblies. (ZIP 33507 kb) [file 40168_2018_579_MOESM4_ESM.zip › 00156.jpg]

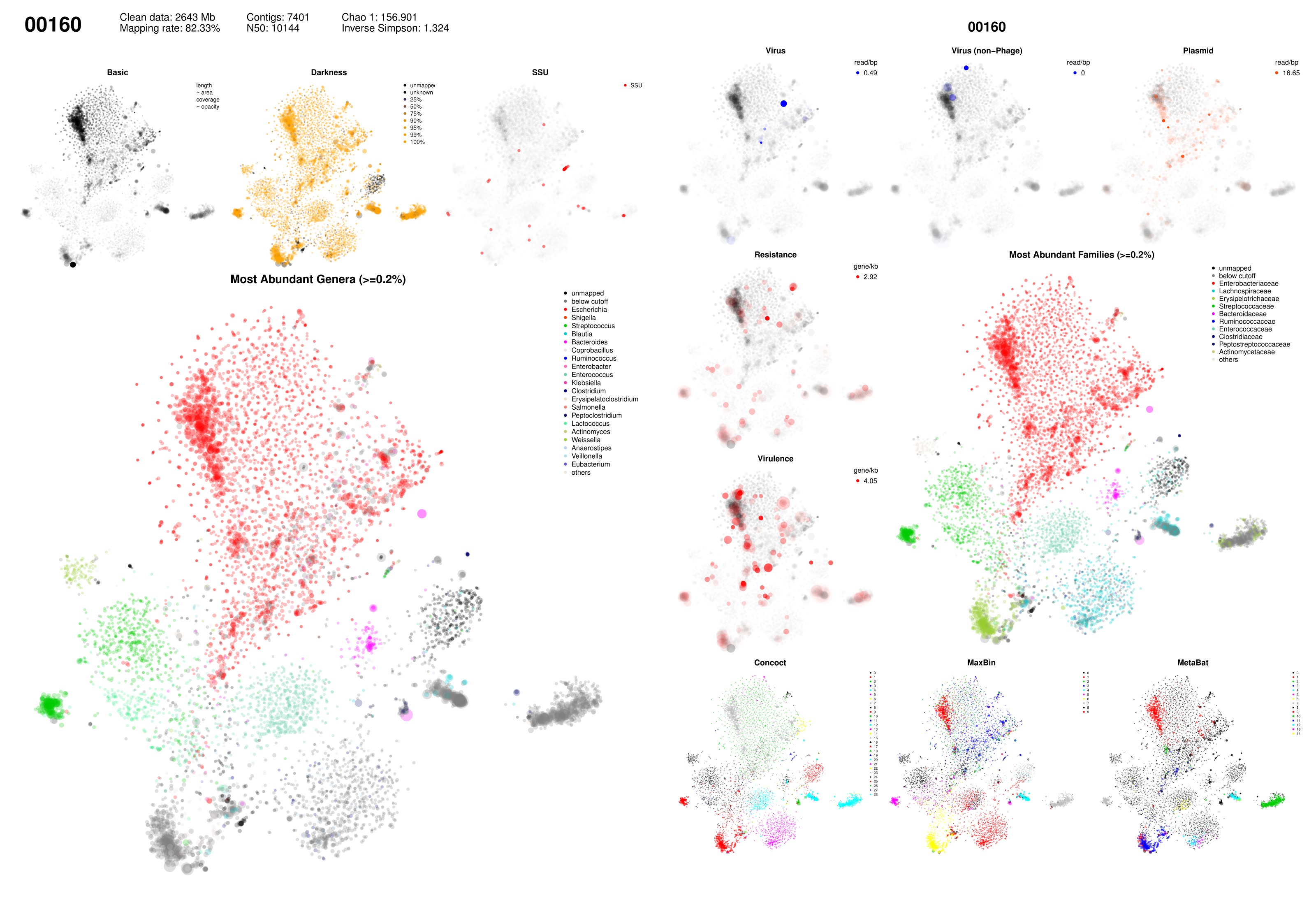

Supplement: Supplementary file 4 — k-mer signature-based scatter plots with multiple features visualized for all 29 metagenomic assemblies. (ZIP 33507 kb) [file 40168_2018_579_MOESM4_ESM.zip › 00160.jpg]

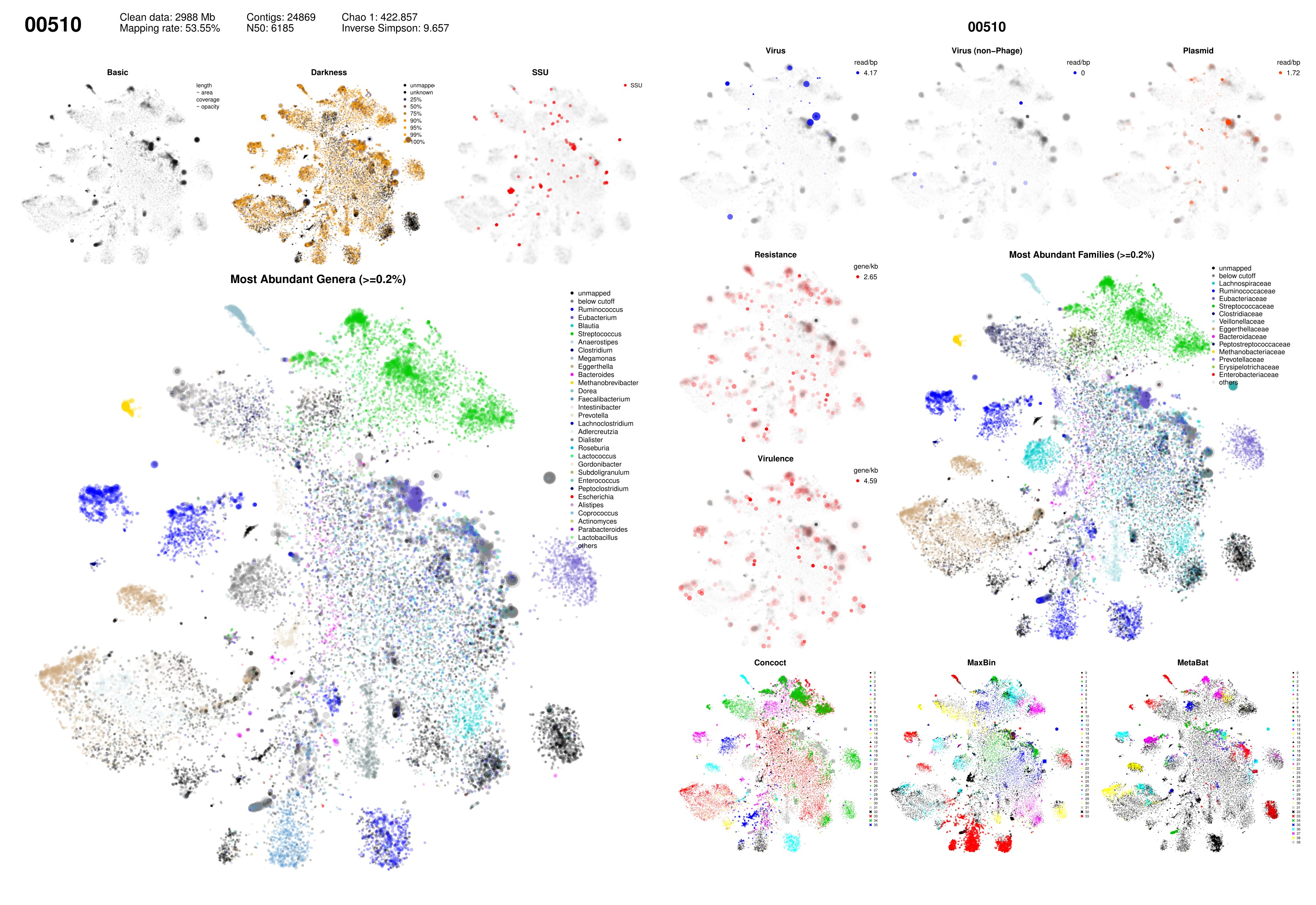

Supplement: Supplementary file 4 — k-mer signature-based scatter plots with multiple features visualized for all 29 metagenomic assemblies. (ZIP 33507 kb) [file 40168_2018_579_MOESM4_ESM.zip › 00510.jpg]

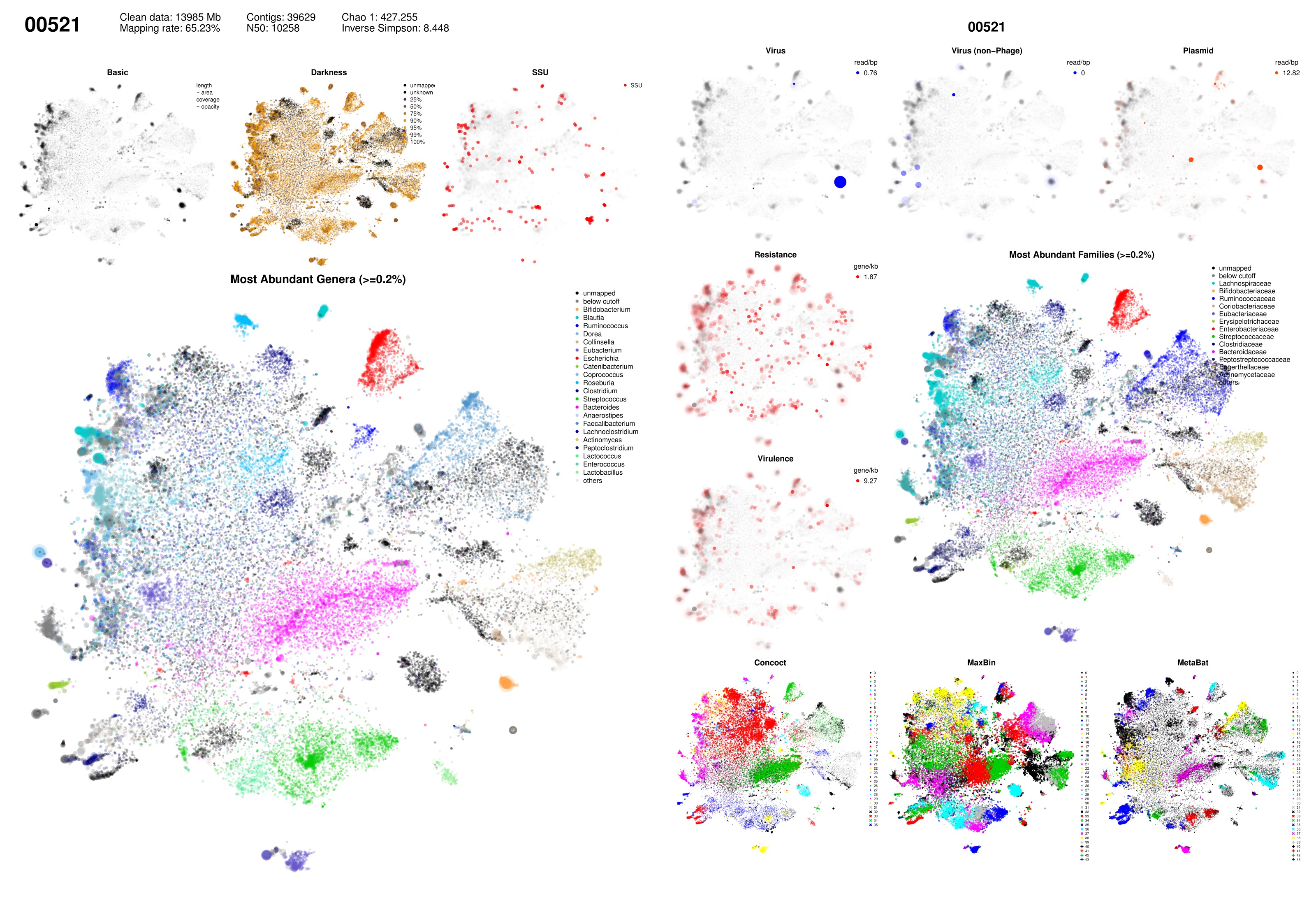

Supplement: Supplementary file 4 — k-mer signature-based scatter plots with multiple features visualized for all 29 metagenomic assemblies. (ZIP 33507 kb) [file 40168_2018_579_MOESM4_ESM.zip › 00521.jpg]

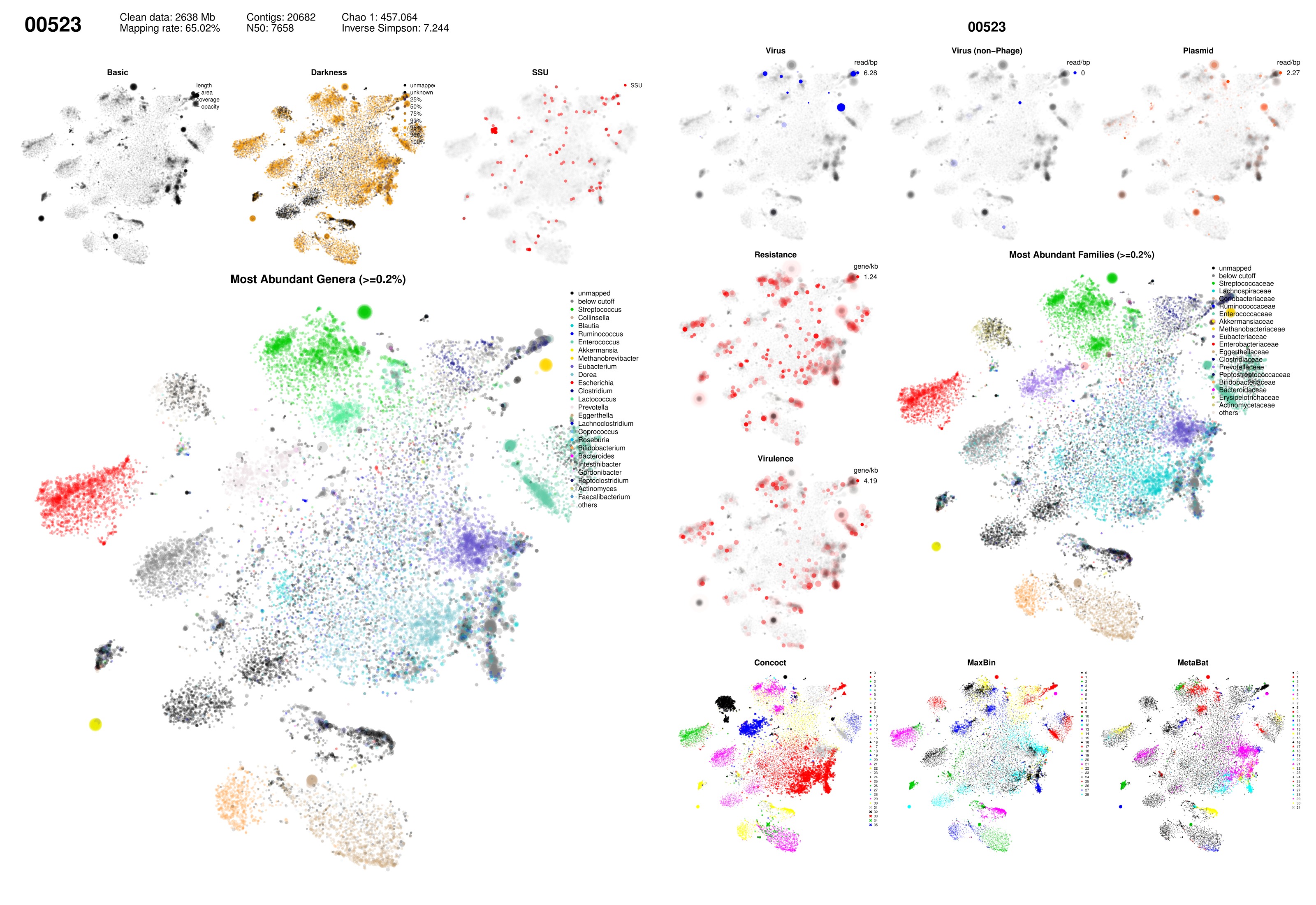

Supplement: Supplementary file 4 — k-mer signature-based scatter plots with multiple features visualized for all 29 metagenomic assemblies. (ZIP 33507 kb) [file 40168_2018_579_MOESM4_ESM.zip › 00523.jpg]

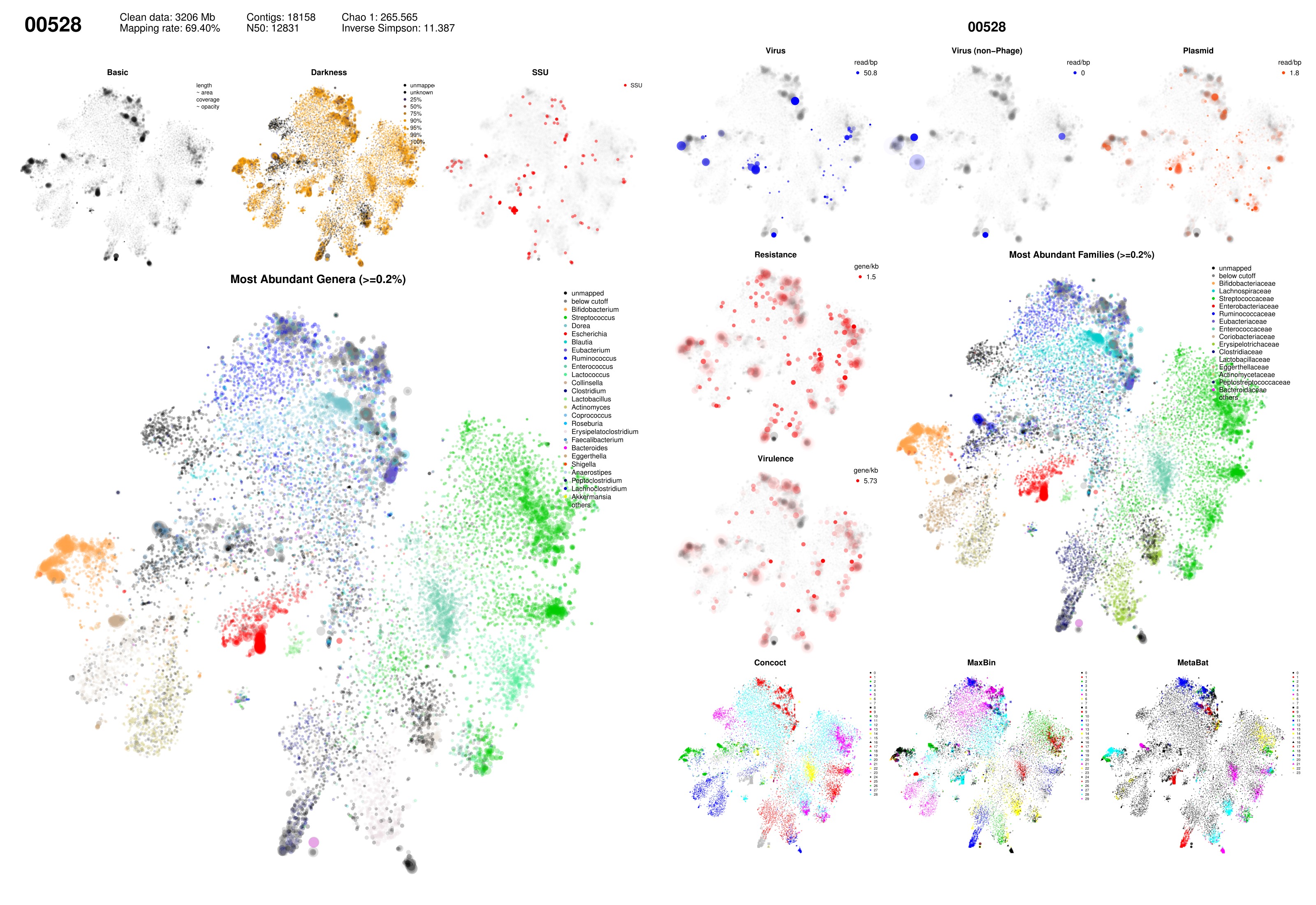

Supplement: Supplementary file 4 — k-mer signature-based scatter plots with multiple features visualized for all 29 metagenomic assemblies. (ZIP 33507 kb) [file 40168_2018_579_MOESM4_ESM.zip › 00528.jpg]

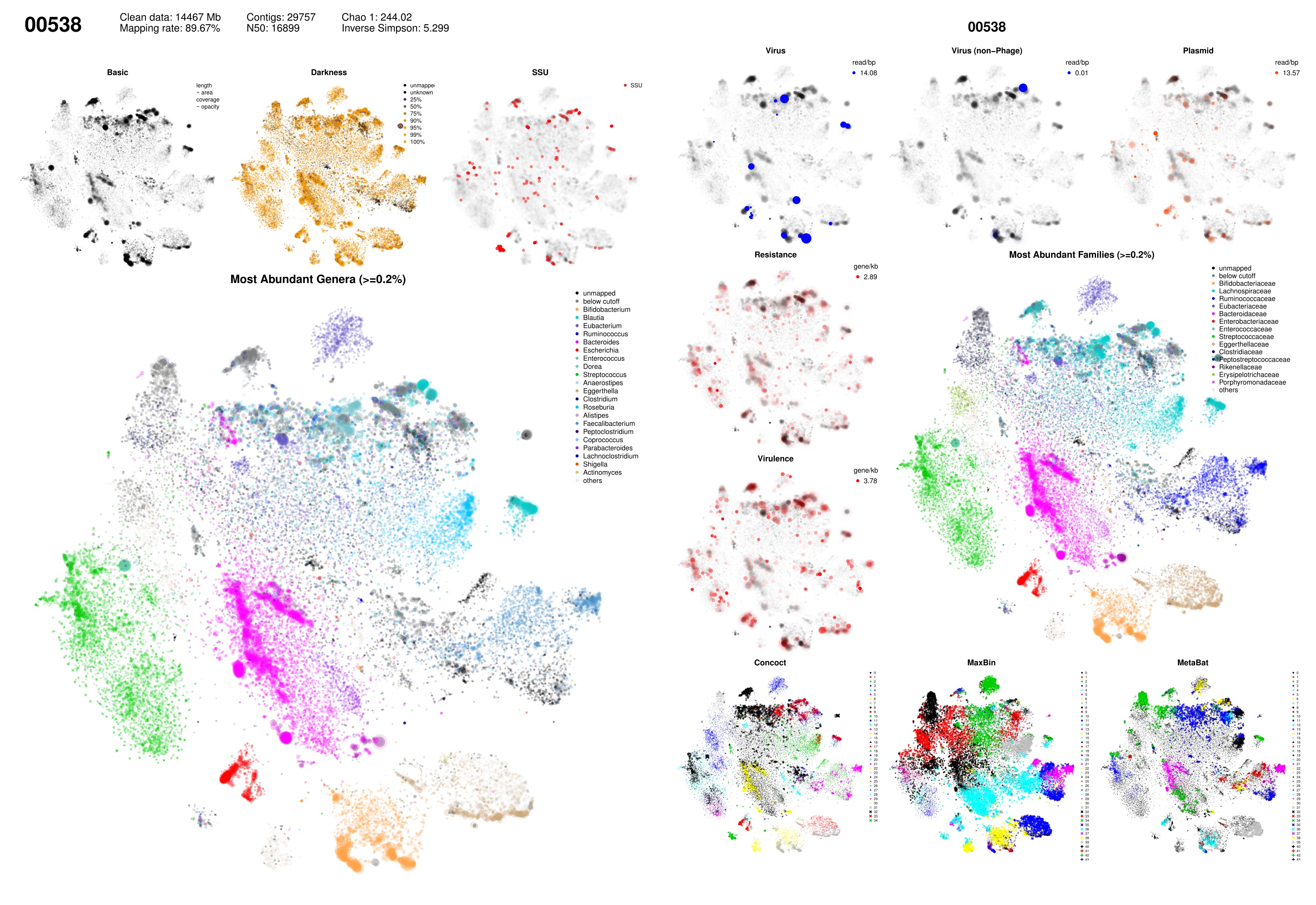

Supplement: Supplementary file 4 — k-mer signature-based scatter plots with multiple features visualized for all 29 metagenomic assemblies. (ZIP 33507 kb) [file 40168_2018_579_MOESM4_ESM.zip › 00538.jpg]

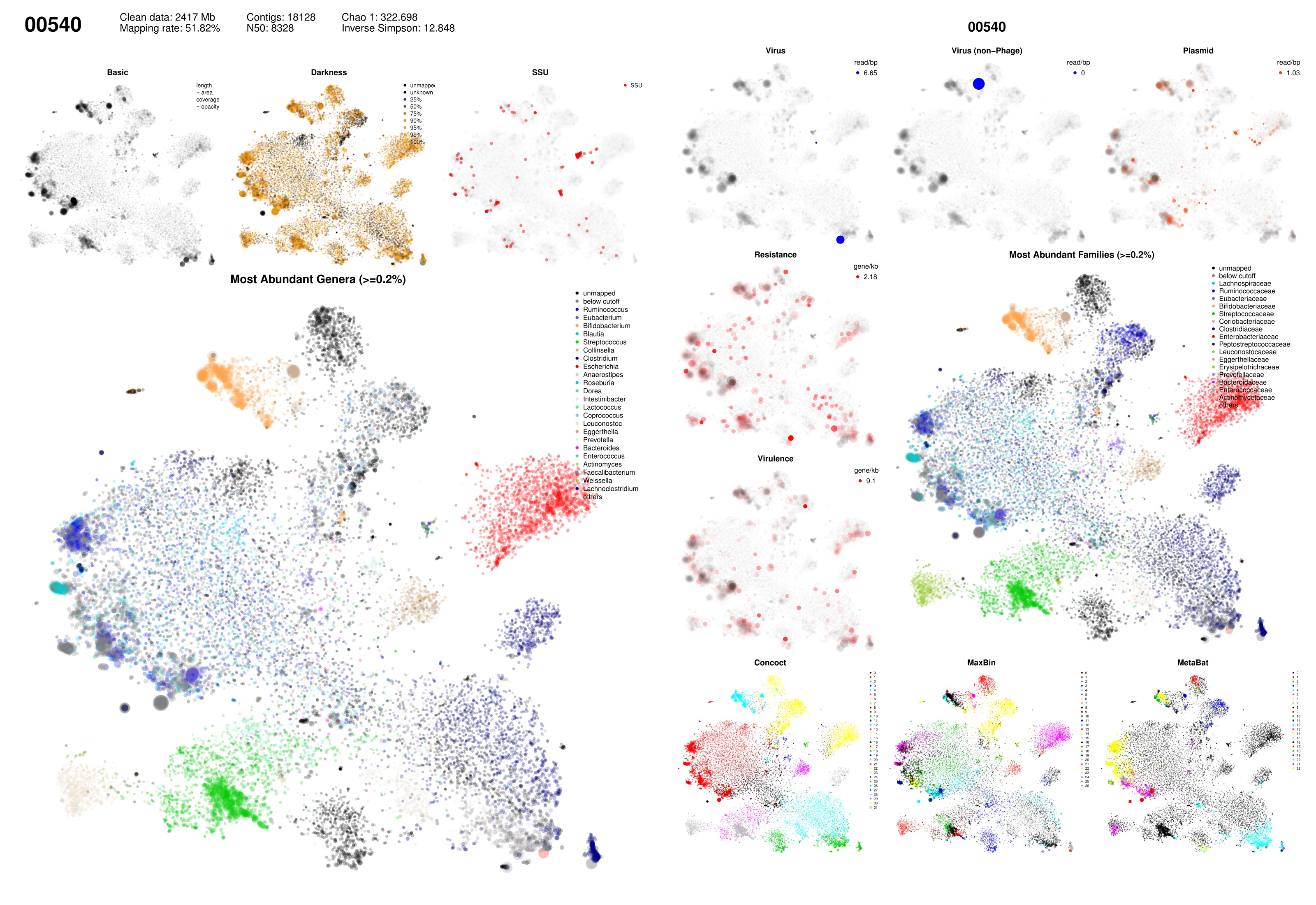

Supplement: Supplementary file 4 — k-mer signature-based scatter plots with multiple features visualized for all 29 metagenomic assemblies. (ZIP 33507 kb) [file 40168_2018_579_MOESM4_ESM.zip › 00540.jpg]

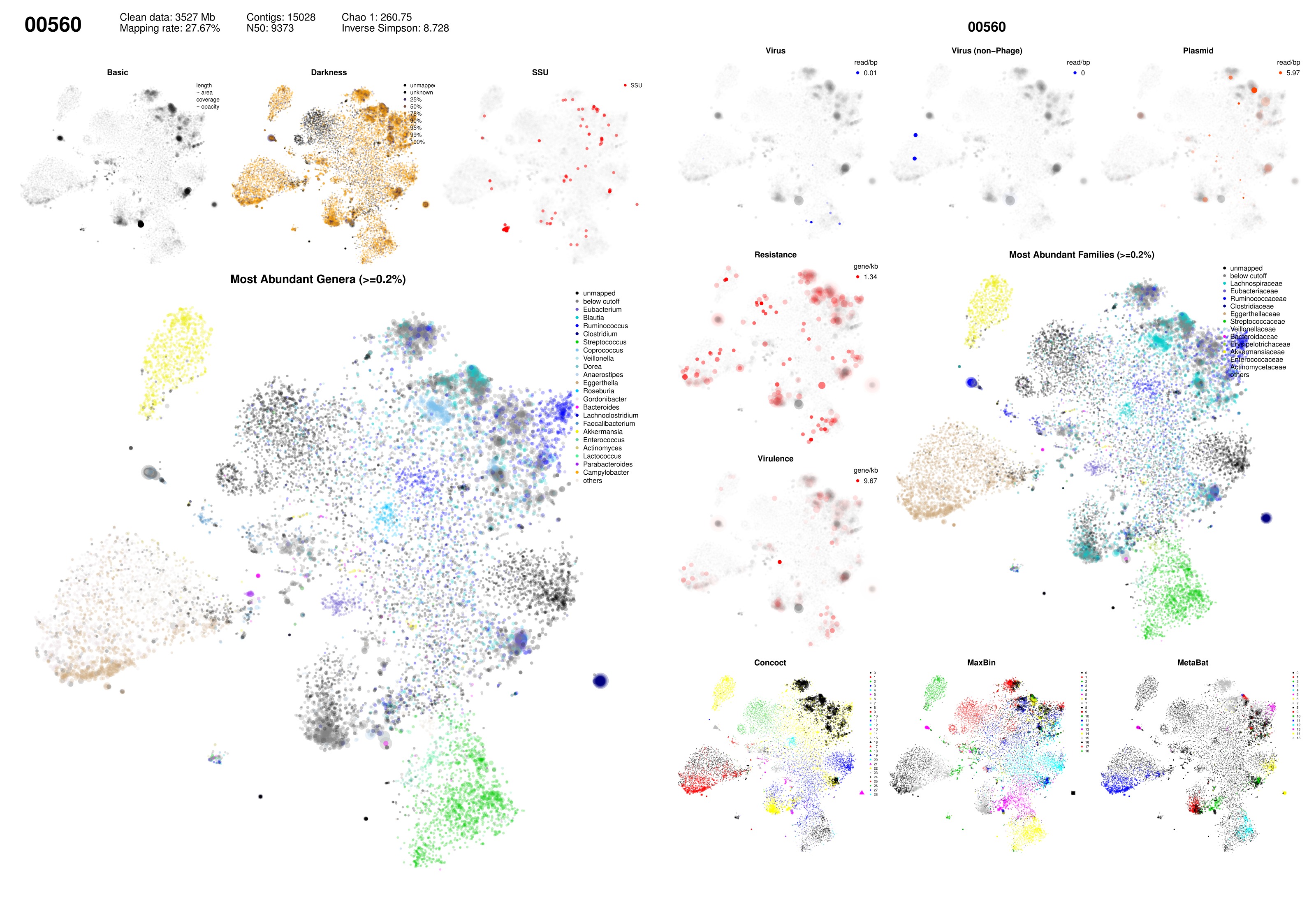

Supplement: Supplementary file 4 — k-mer signature-based scatter plots with multiple features visualized for all 29 metagenomic assemblies. (ZIP 33507 kb) [file 40168_2018_579_MOESM4_ESM.zip › 00560.jpg]

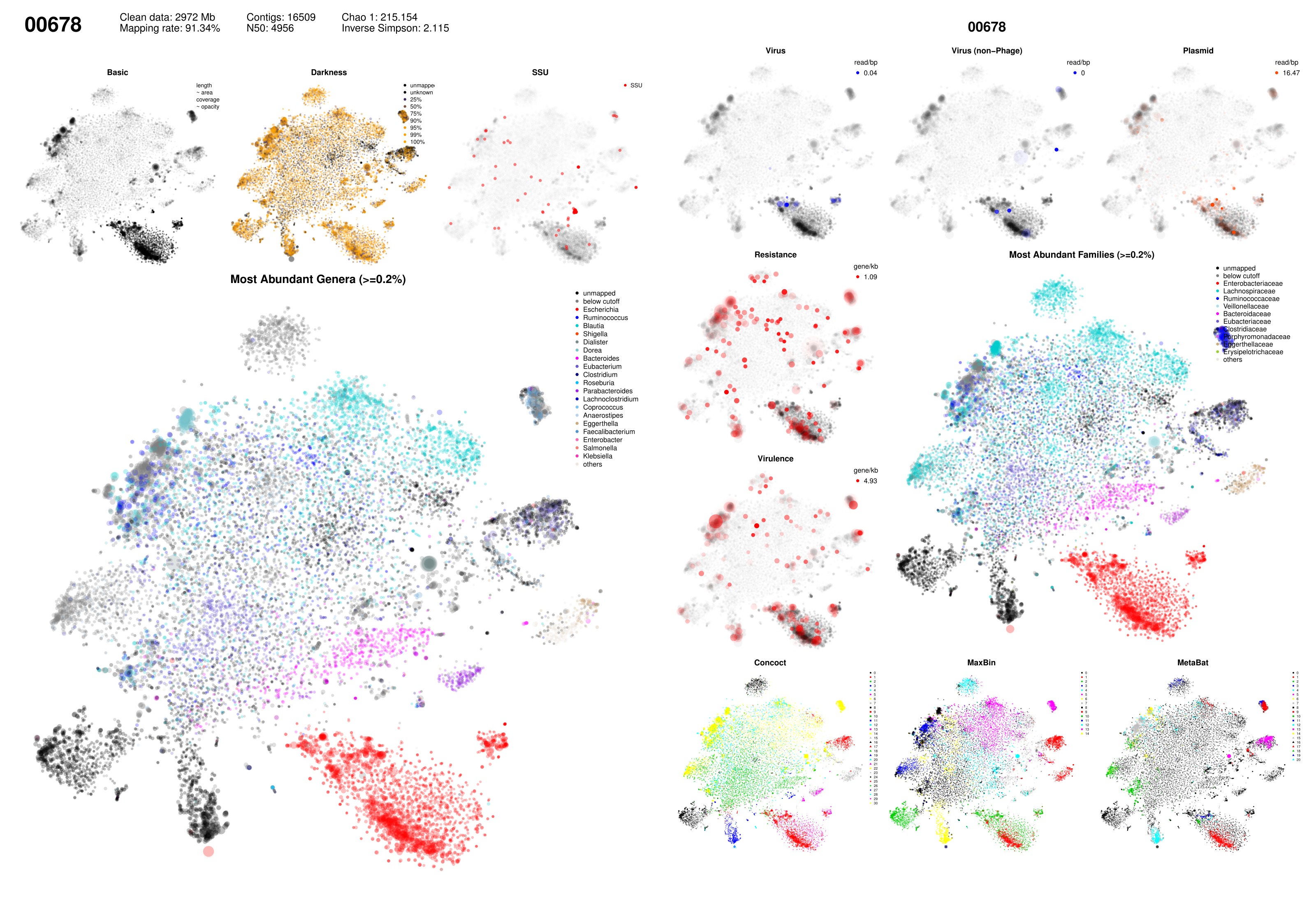

Supplement: Supplementary file 4 — k-mer signature-based scatter plots with multiple features visualized for all 29 metagenomic assemblies. (ZIP 33507 kb) [file 40168_2018_579_MOESM4_ESM.zip › 00678.jpg]

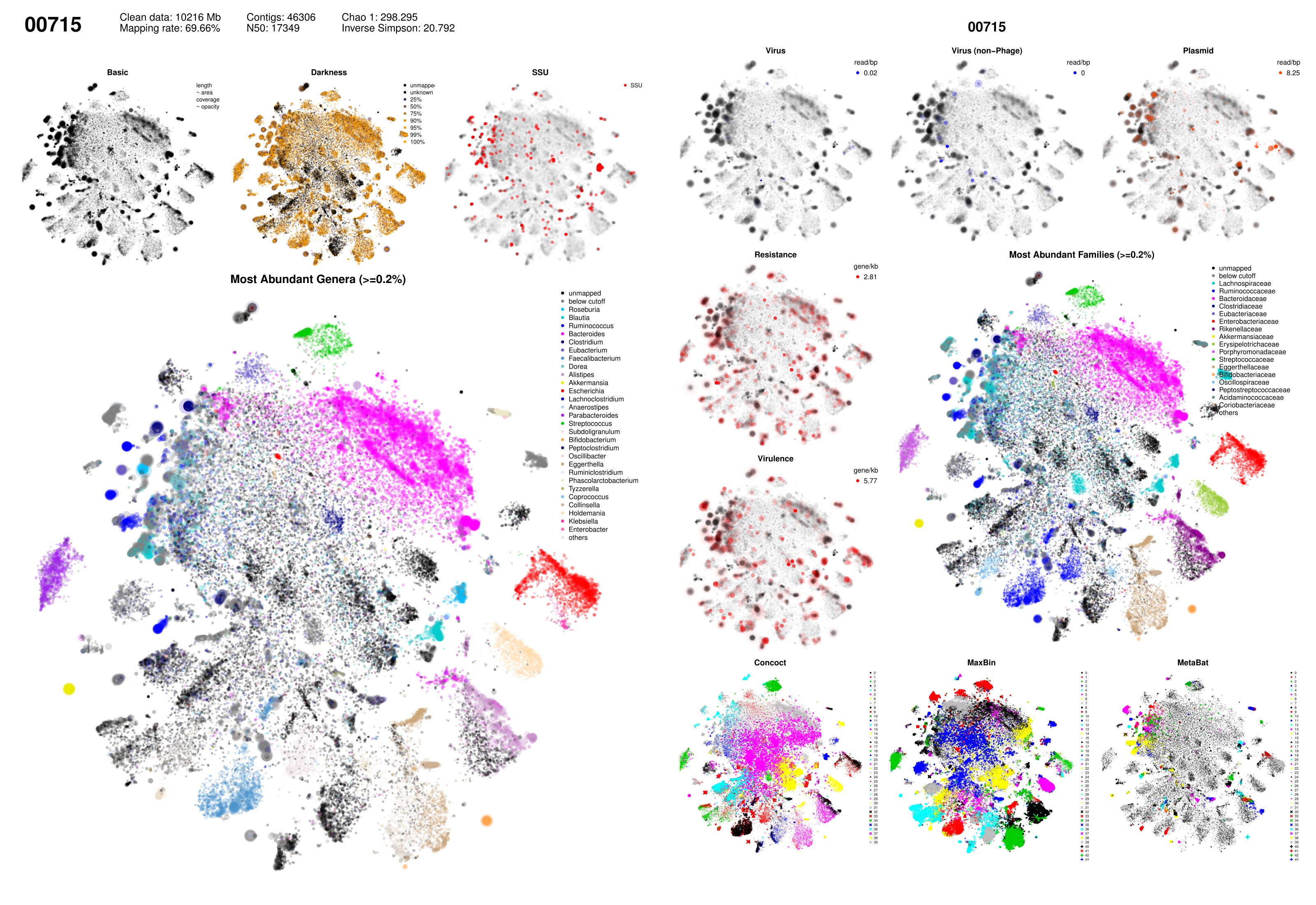

Supplement: Supplementary file 4 — k-mer signature-based scatter plots with multiple features visualized for all 29 metagenomic assemblies. (ZIP 33507 kb) [file 40168_2018_579_MOESM4_ESM.zip › 00715.jpg]

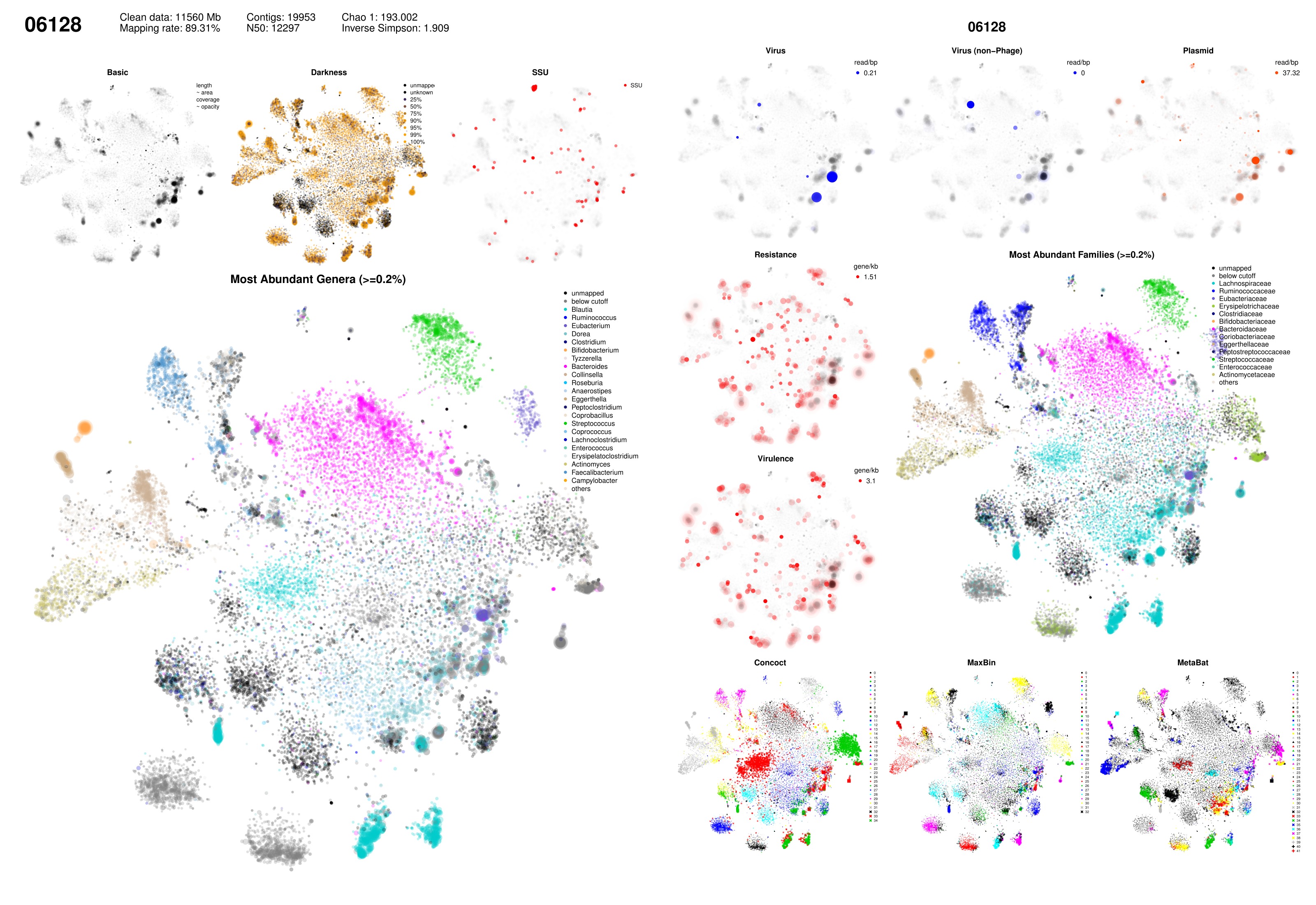

Supplement: Supplementary file 4 — k-mer signature-based scatter plots with multiple features visualized for all 29 metagenomic assemblies. (ZIP 33507 kb) [file 40168_2018_579_MOESM4_ESM.zip › 06128.jpg]

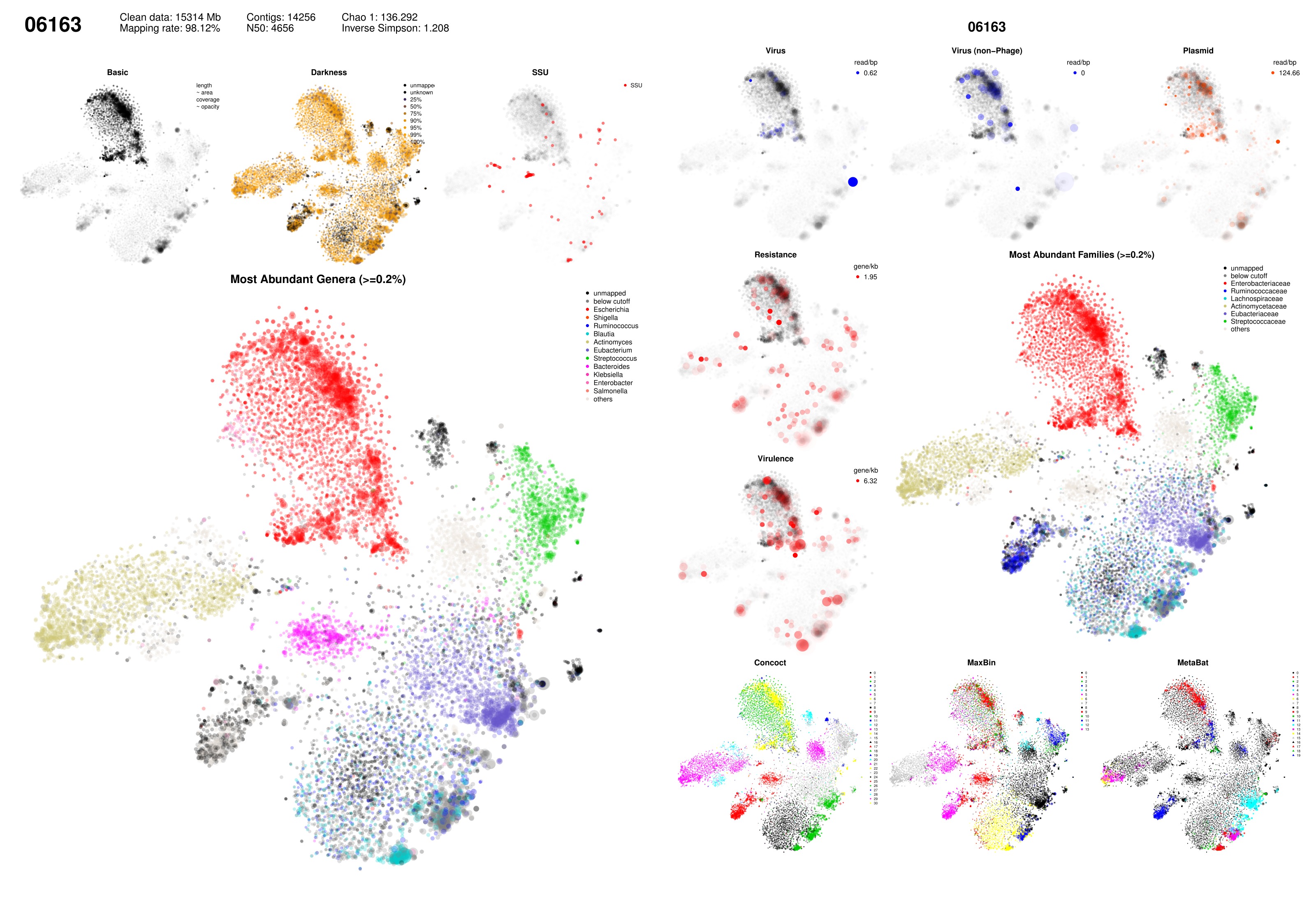

Supplement: Supplementary file 4 — k-mer signature-based scatter plots with multiple features visualized for all 29 metagenomic assemblies. (ZIP 33507 kb) [file 40168_2018_579_MOESM4_ESM.zip › 06163.jpg]

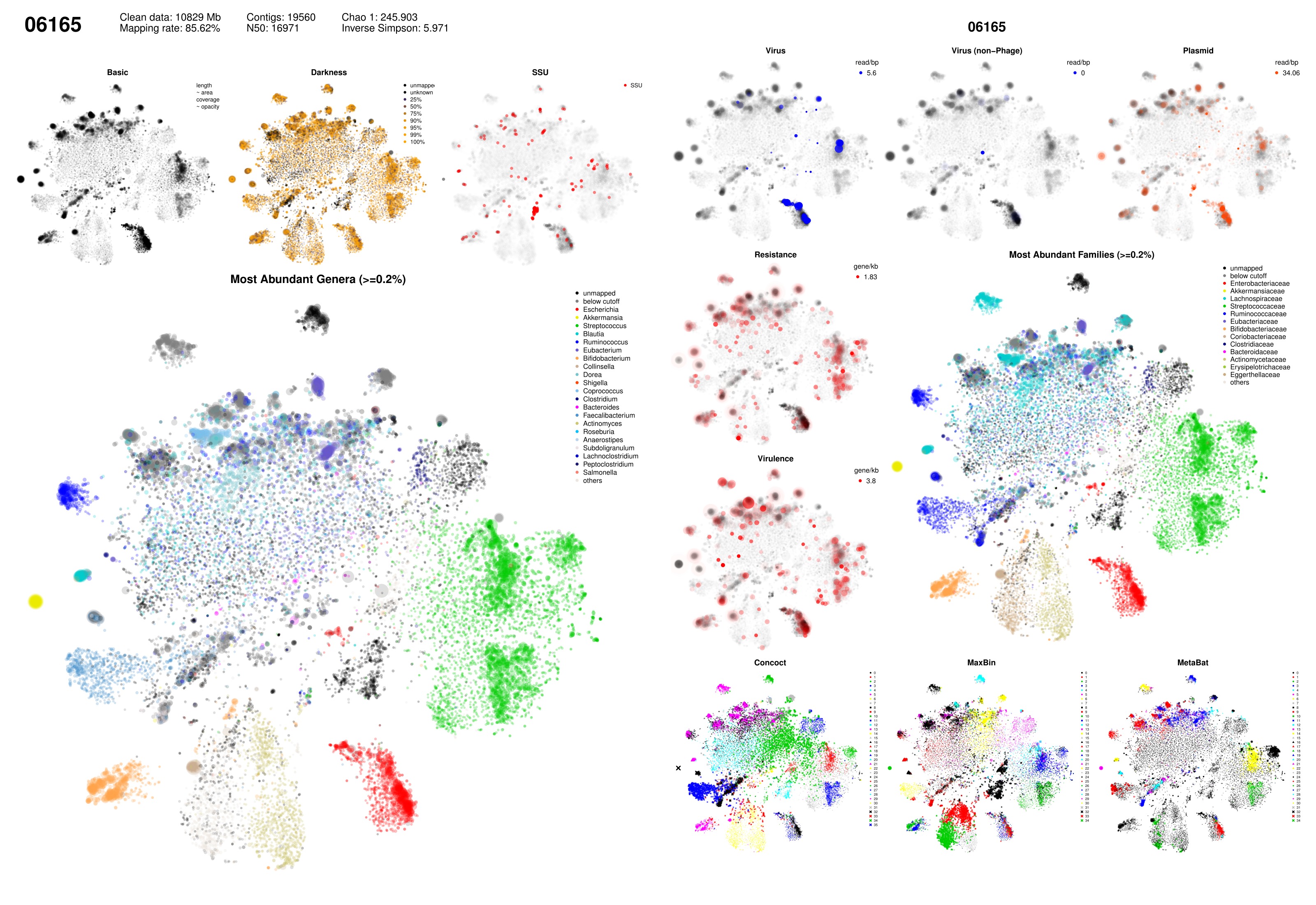

Supplement: Supplementary file 4 — k-mer signature-based scatter plots with multiple features visualized for all 29 metagenomic assemblies. (ZIP 33507 kb) [file 40168_2018_579_MOESM4_ESM.zip › 06165.jpg]

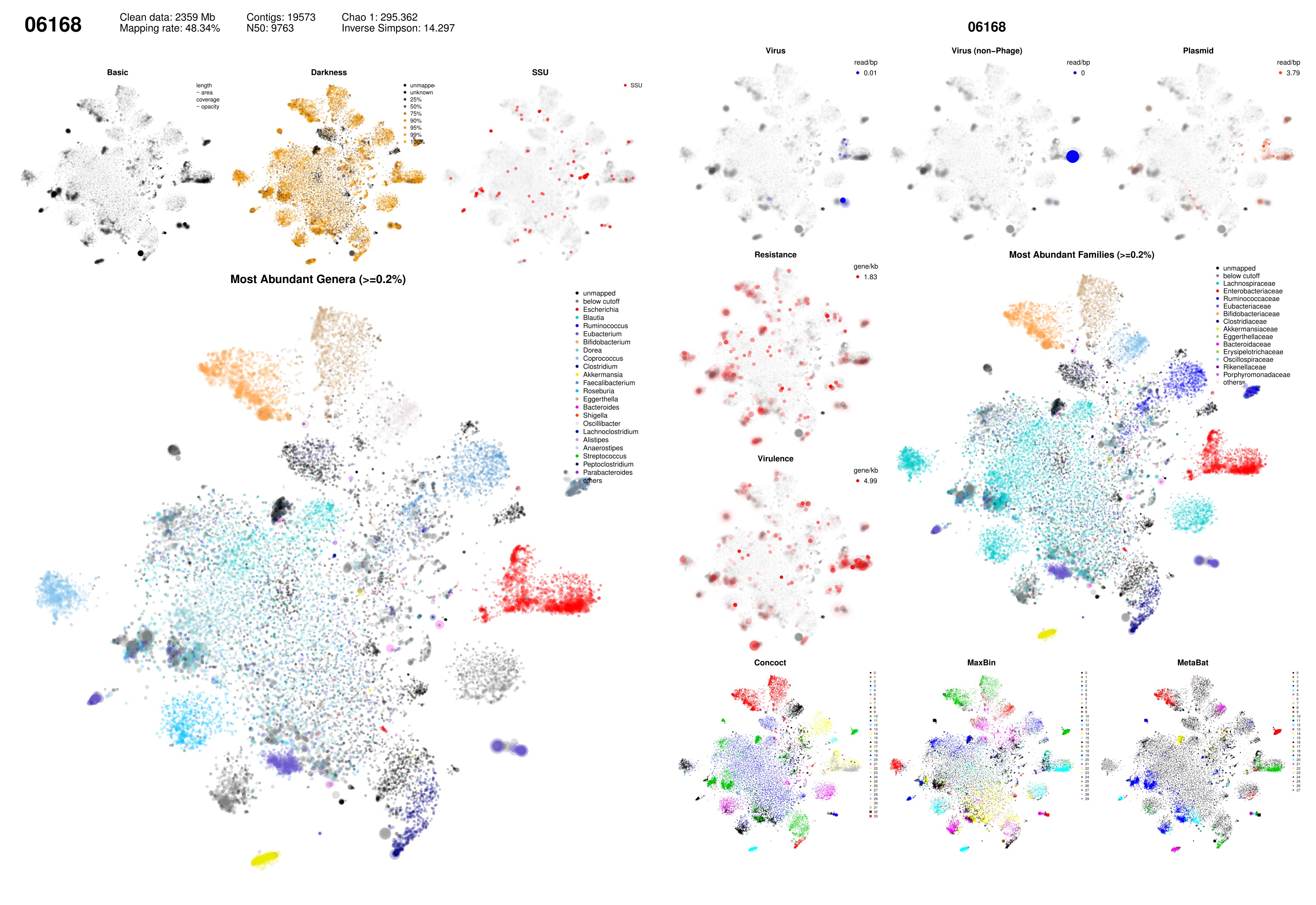

Supplement: Supplementary file 4 — k-mer signature-based scatter plots with multiple features visualized for all 29 metagenomic assemblies. (ZIP 33507 kb) [file 40168_2018_579_MOESM4_ESM.zip › 06168.jpg]

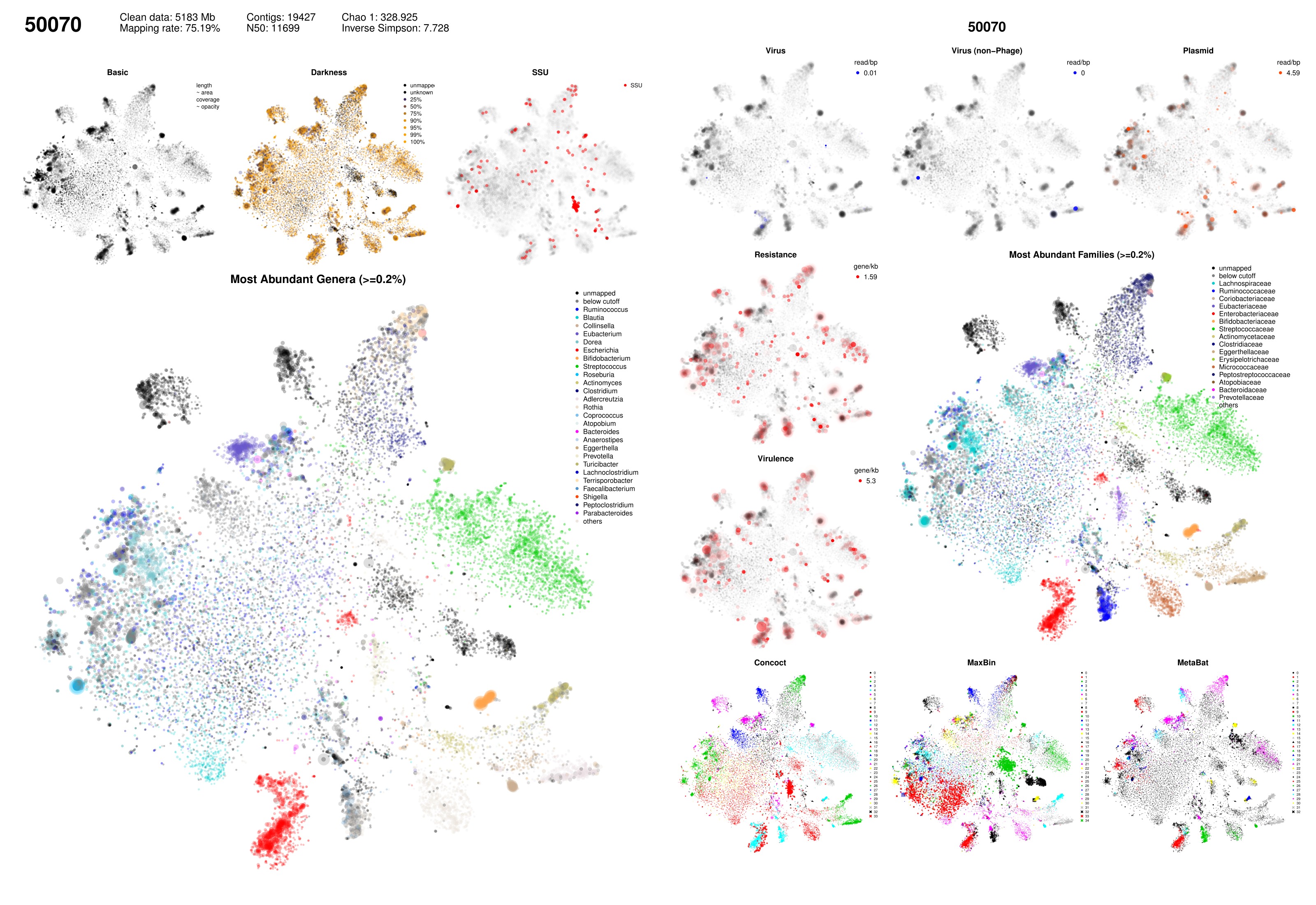

Supplement: Supplementary file 4 — k-mer signature-based scatter plots with multiple features visualized for all 29 metagenomic assemblies. (ZIP 33507 kb) [file 40168_2018_579_MOESM4_ESM.zip › 50070.jpg]

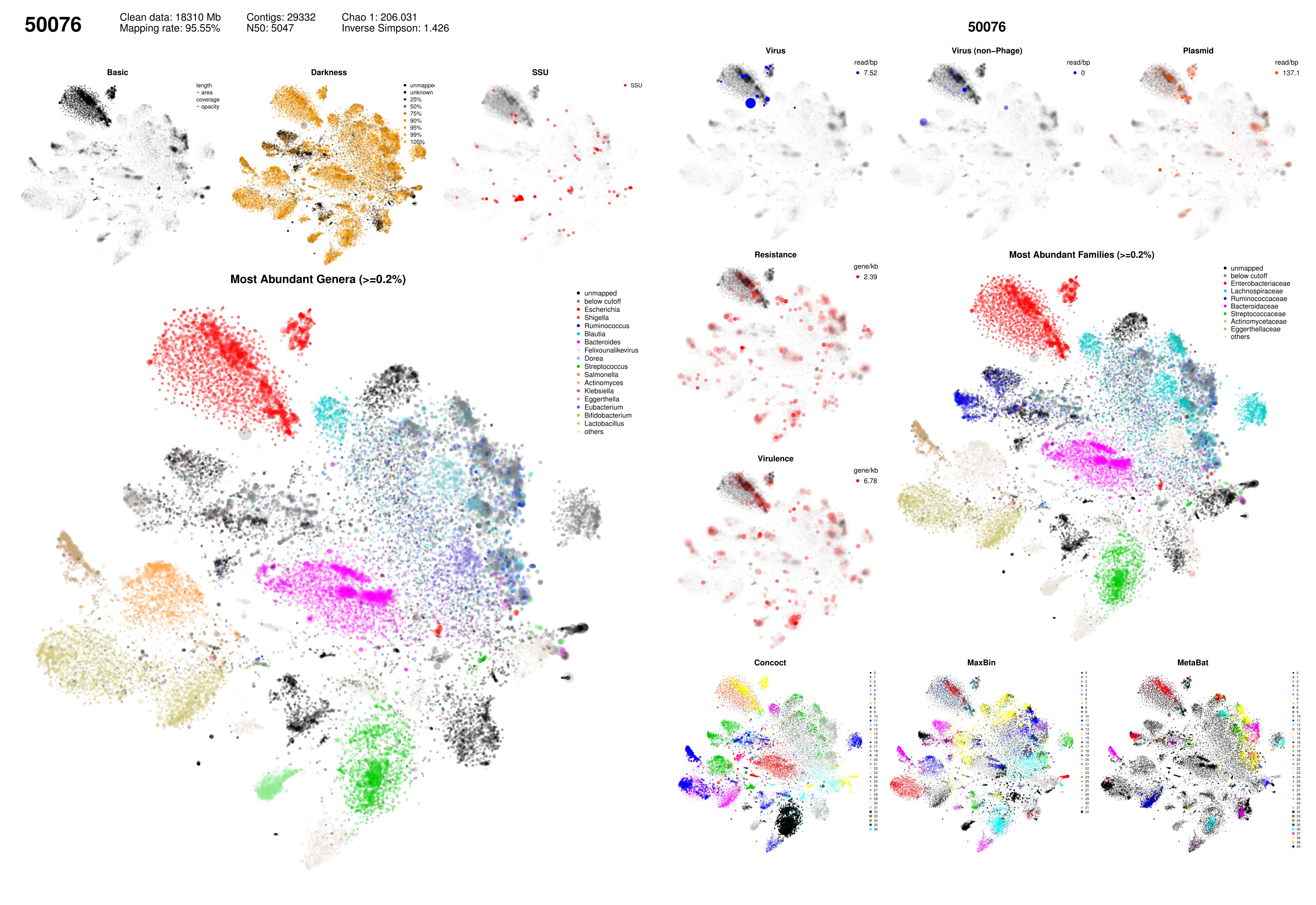

Supplement: Supplementary file 4 — k-mer signature-based scatter plots with multiple features visualized for all 29 metagenomic assemblies. (ZIP 33507 kb) [file 40168_2018_579_MOESM4_ESM.zip › 50076.jpg]

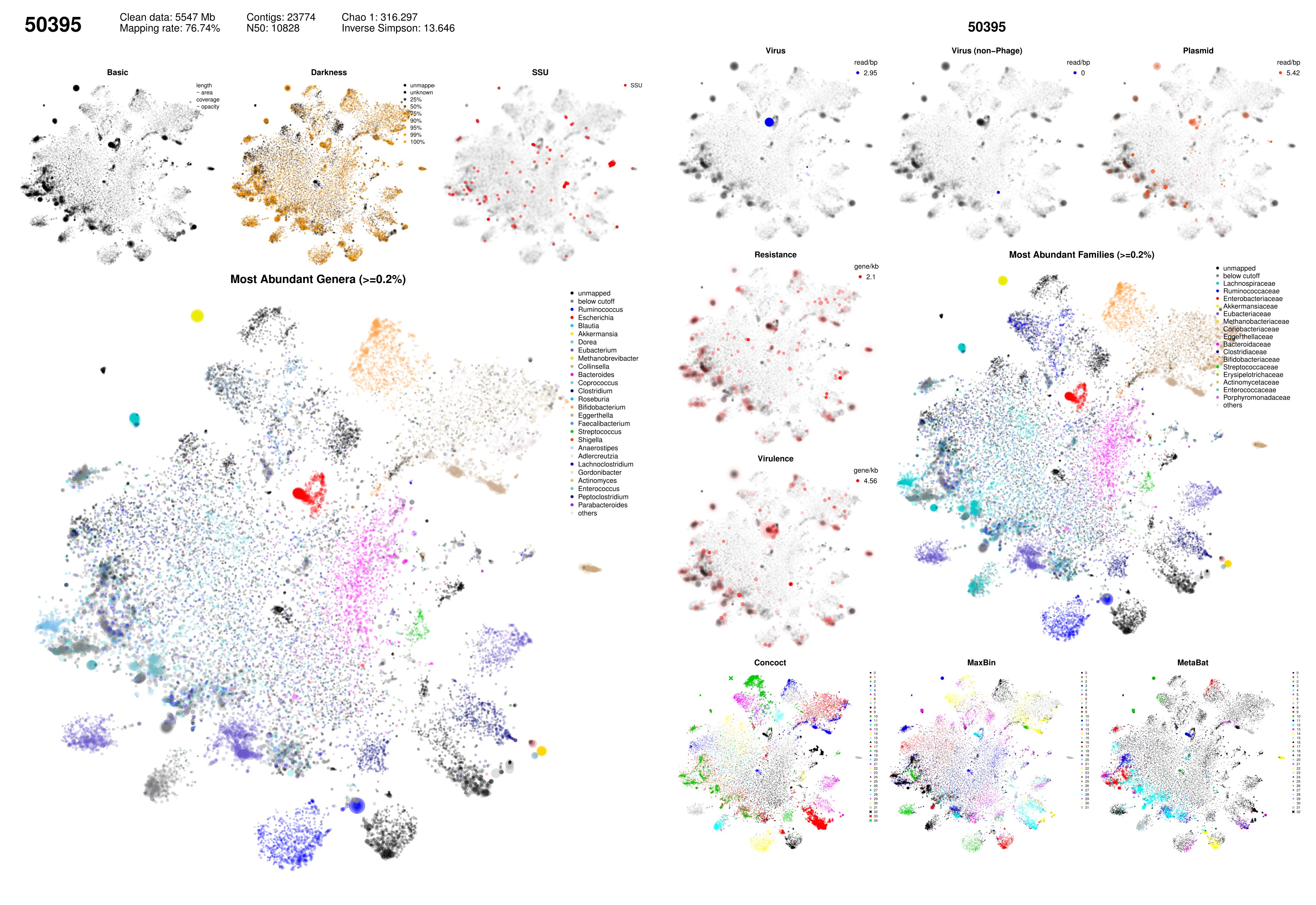

Supplement: Supplementary file 4 — k-mer signature-based scatter plots with multiple features visualized for all 29 metagenomic assemblies. (ZIP 33507 kb) [file 40168_2018_579_MOESM4_ESM.zip › 50395.jpg]

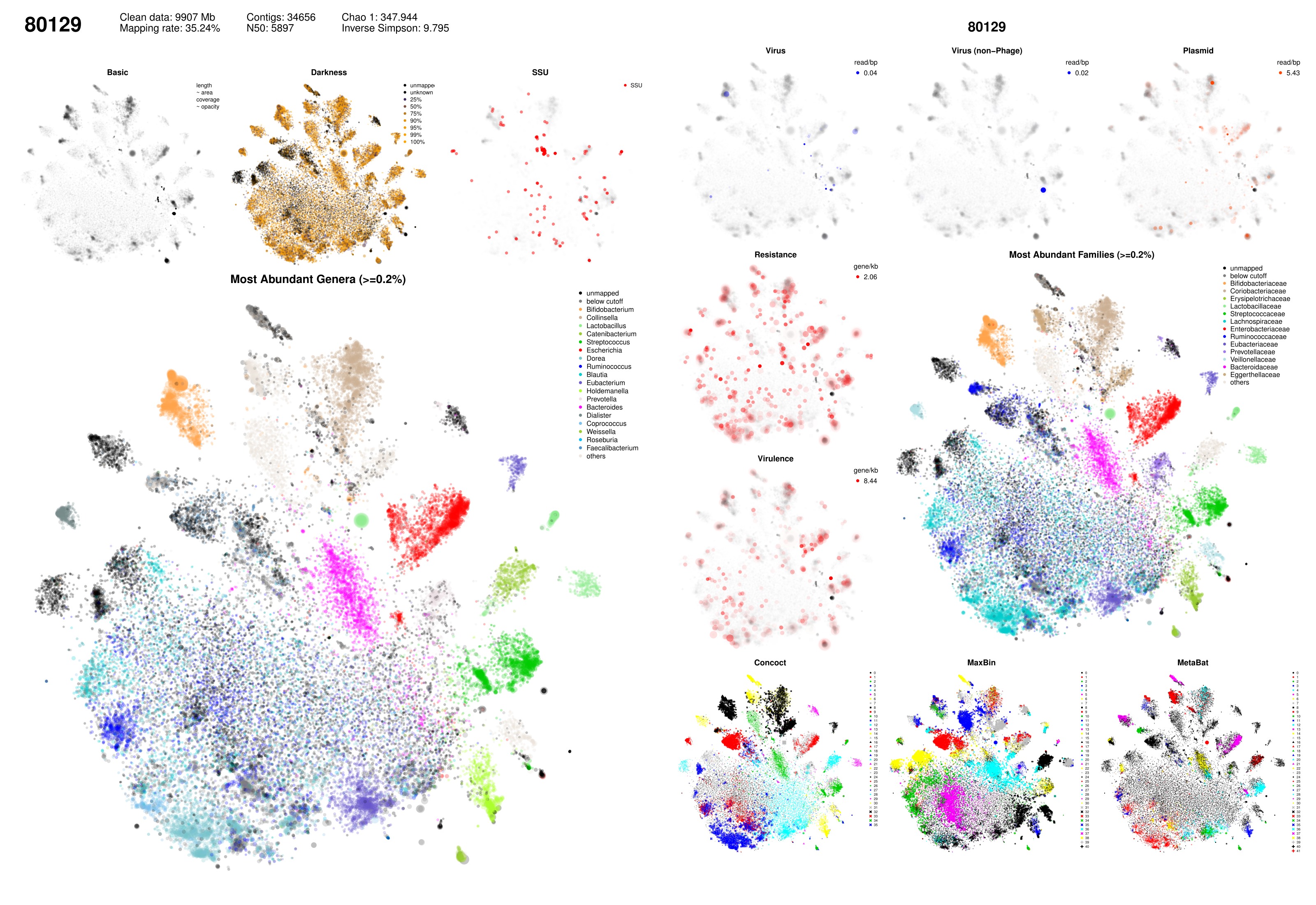

Supplement: Supplementary file 4 — k-mer signature-based scatter plots with multiple features visualized for all 29 metagenomic assemblies. (ZIP 33507 kb) [file 40168_2018_579_MOESM4_ESM.zip › 80129.jpg]

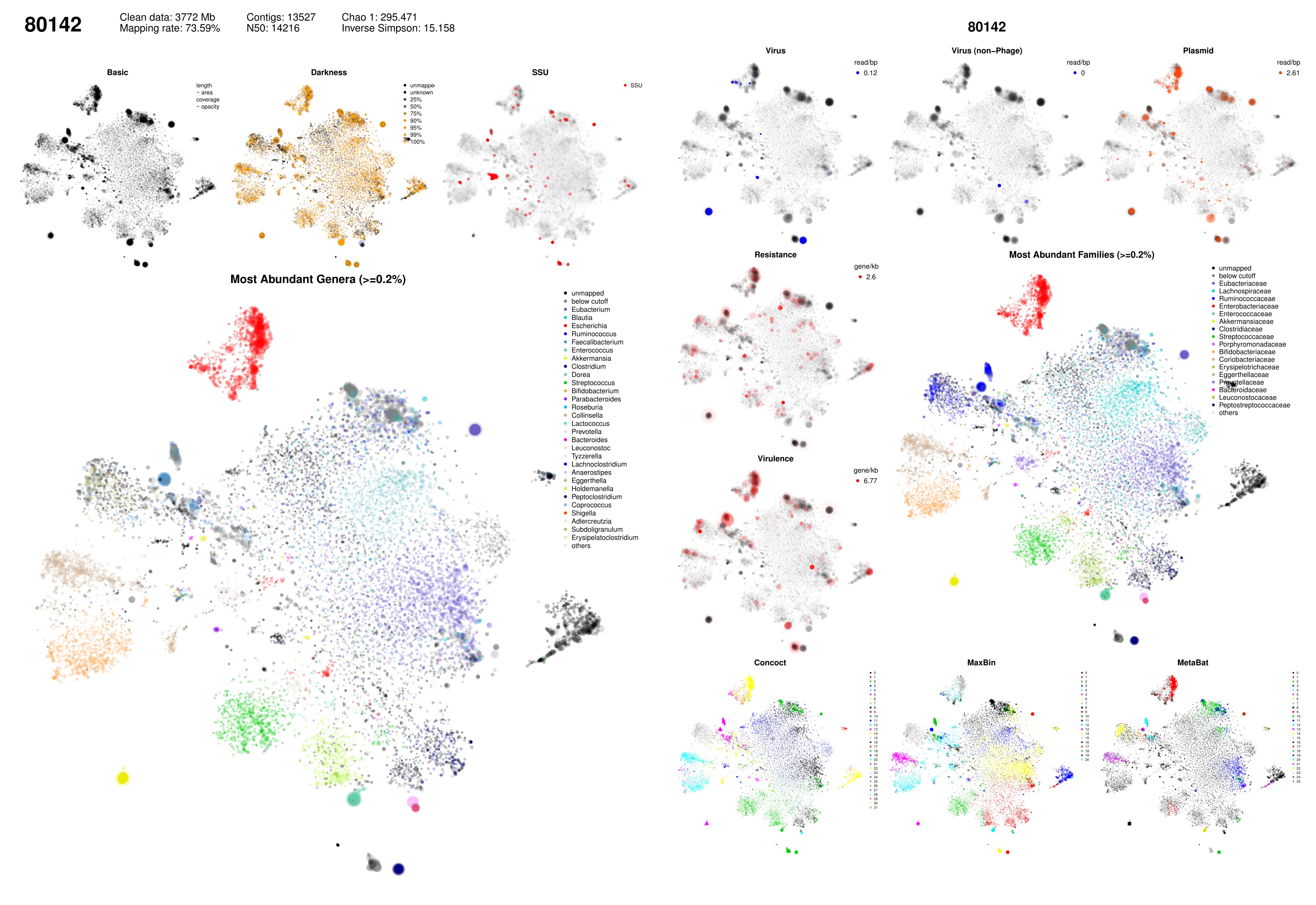

Supplement: Supplementary file 4 — k-mer signature-based scatter plots with multiple features visualized for all 29 metagenomic assemblies. (ZIP 33507 kb) [file 40168_2018_579_MOESM4_ESM.zip › 80142.jpg]

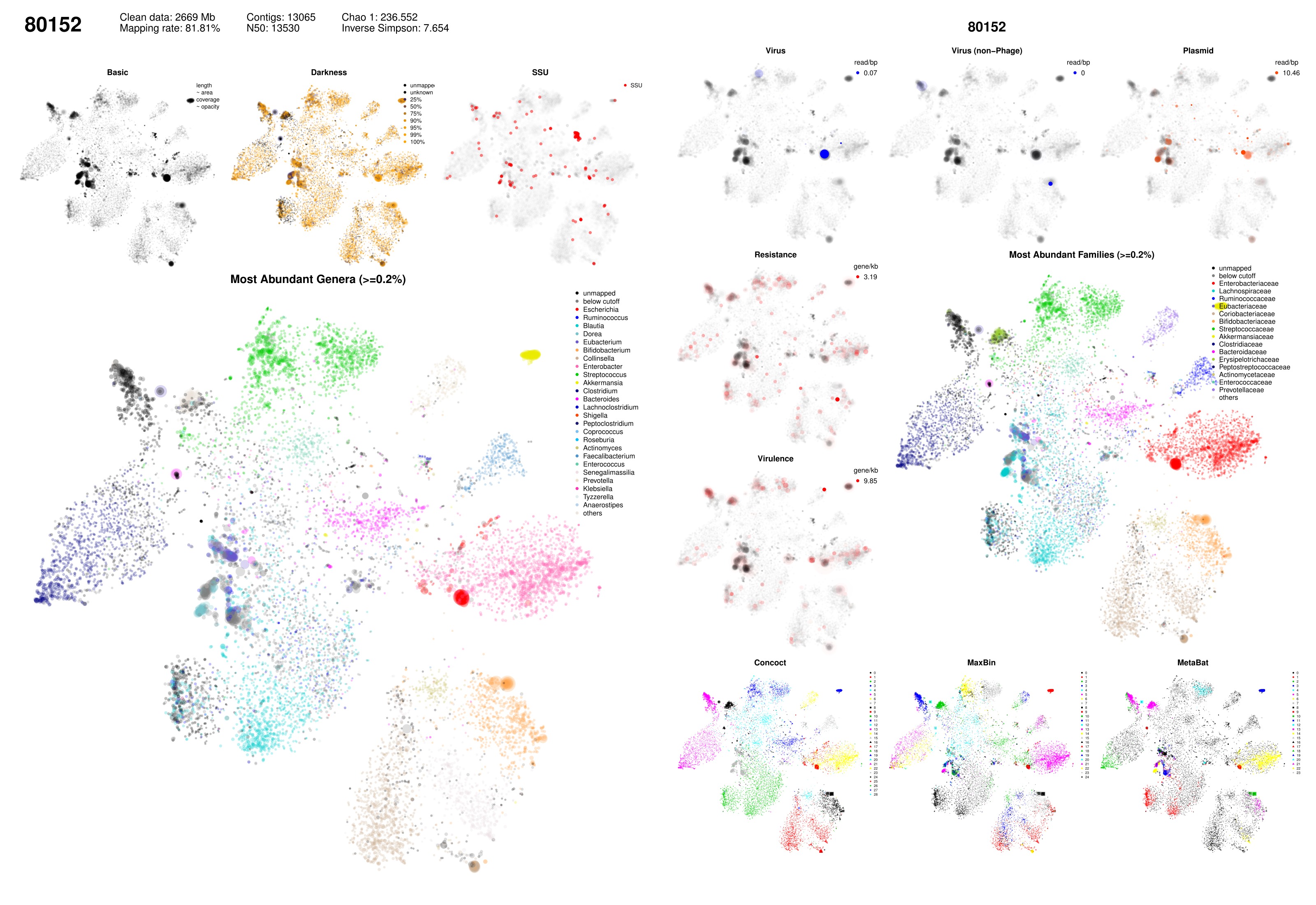

Supplement: Supplementary file 4 — k-mer signature-based scatter plots with multiple features visualized for all 29 metagenomic assemblies. (ZIP 33507 kb) [file 40168_2018_579_MOESM4_ESM.zip › 80152.jpg]
